# Au_76_(SC_6_H_4_‑*p*‑CH_3_)_42_ Square Quantum Platelet: One-Dimensional Growth of Quantum Rods Turns 90 Degrees

**DOI:** 10.1021/jacs.5c14654

**Published:** 2025-11-10

**Authors:** Yitong Wang, Avirup Sardar, Zhongyu Liu, Christopher G. Gianopoulos, Guiying He, Xiaolin Liu, Sihan Chen, Kristin Kirschbaum, Abhrojyoti Mazumder, Mircea Cotlet, De-en Jiang, Rongchao Jin

**Affiliations:** † Department of Chemistry, 6612 Carnegie Mellon University, Pittsburgh, Pennsylvania 15213, United States; ‡ Department of Chemistry and Biochemistry, 7923 University of Toledo, Toledo, Ohio 43606, United States; § Department of Chemical and Biomolecular Engineering, 5718 Vanderbilt University, Nashville, Tennessee 37235, United States; ∥ Center for Functional Nanomaterials, 8099 Brookhaven National Laboratory, Upton, New York 11973, United States

## Abstract

The growth pattern of atomically precise nanoclusters (NCs) is of fundamental interest, and the structural effect on their photoluminescence (PL) is important owing to their PL in the second near-infrared (NIR-II) range (900–1700 nm optoelectronically or 1000–1700 nm biologically) that holds great promise for optoelectronic and biomedical applications. Because of the small energy gaps required for NIR-II emission, the PL performance of NIR-II luminophores is largely limited by nonradiative processes. In this work, we discovered a Au_76_(*p*-MBT)_42_ (**Au**_**76**_) (*p*-MBTH = *p*-methylbenzenethiol) nanocluster featuring a face-centered cubic (fcc) core in a square shape (edge length: 1 nm). This square quantum platelet can be viewed as a side-facet (010) growth of the Au_52_(*p*-MBT)_32_ (**Au**_**52**_) rod, as opposed to the (001) facet growth. We found that **Au**_**76**_ exhibits bright emission centered at 970 nm with a PL quantum yield (PLQY) of 30% in solution under ambient conditions, which can be further enhanced to 40% when the solution is deaerated. X-ray crystallography analysis coupled with time-resolved spectroscopy revealed that the nearly doubled PLQY compared to **Au**_**52**_ (18.3%) was resulted from shorter Au–Au bond lengths in **Au**_**76**_ (average 2.835 Å) than that in **Au**_**52**_ (3.04 Å). This work provides important insights into the design of highly luminescent NCs, which are promising for photovoltaics, photocatalysis, and optoelectronic applications.

## Graphical abstract

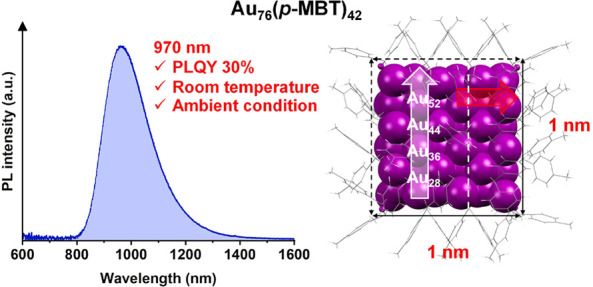

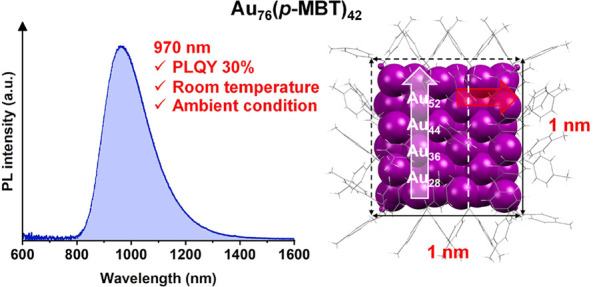

## Introduction

Materials with luminescence in the second near-infrared (NIR-II) region (e.g., 900–1700 nm in optoelectronics, or 1000–1700 nm in bioimaging) are critically needed in many fields, such as deep-tissue biological imaging and optoelectronic devices.^1–3^ Recently, atomically precise gold nanoclusters (Au NCs) have emerged as a new class of NIR-II emissive materials.^4–17^ Such NCs possess several advantages, including high stability, low toxicity, and ease of preparation via wet chemistry. As an atomically precise class of nanomaterials, the shape, size, and structure of NCs play a major role in controlling the photophysical properties of NCs.^18–22^

Structurally, the NCs typically consist of a metal core of high symmetry and a shell of metal-containing complex-like staple motifs.^23–26^ The growth patterns of Au_*n*_(SR)_*m*_ nanoclusters are particularly intriguing,^4–8^ such as the 1D growth of a periodic series of fcc rods, including Au_28_(SR)_20_, Au_36_(SR)_24_, Au_44_(SR)_28_, and Au_52_(SR)_32_ with a uniform increment of 8 gold atoms and 4 thiolate ligands.^4–6^ Longer rods are expected to show strong polarization of electronic transitions;^5,10^ however, there has been no success yet in further elongation of Au_52_(SR)_32_ to longer ones such as Au_60_(SR)_36_, and Au_68_(SR)_40_ as theoretically predicted.^5–8^ Thus, a fundamental question still remains: how would the growth pattern further extend?

With respect to the optical properties of nanoclusters, previous studies have shown that their photoluminescence quantum yield (PLQY) is largely associated with the nonradiative processes arising from the core structure and surface motifs.^27,28^ Structural rigidification can be achieved by either kernel engineering^29,30^ or tailoring the surface ligands.^30–33^ Recently, Shi et al. reported a highly luminescent bimetallic nanocluster of Au_16_Cu_6_(^*t*^Bu-C_6_H_4_C≡C)_18_, which emits at 720 nm with a near-unity PLQY in degassed solvents.^34^ Generally, as the number of gold atoms in the core increases, the energy gap between the highest occupied molecular orbital (HOMO) and the lowest unoccupied molecular orbital (LUMO) shrinks, which results in longer wavelength emission.^35^ However, when the emission wavelength shifts toward the NIR-II region (900–1700 nm), the PLQY drops significantly and becomes largely limited by the nonradiative relaxation per the energy-gap law.^36,37^ Thus, this calls for new designs of NCs.

In this work, we successfully synthesized and crystallized a Au_76_(*p*-MBT)_42_ (abbrev. **Au_76_**) nanocluster. It has an fcc-type core in a square shape (edge length: 1 nm), which can be viewed as a lateral extension of Au_52_(*p*-MBT)_32_ (**Au**_**52**_) along a side facet of **Au**_**52**_.^32^ The **Au**_**76**_ shows NIR-II photoluminescence centered at 970 nm with PLQY of 30% in solution under ambient conditions and 40% in a deaerated solution. The PL lifetime is 690 ns (deaerated) and 410 ns (non-deaerated), which are comparable to other gold fcc NCs protected by aromatic ligands.^37–40^ Femtosecond and nanosecond transient absorption spectroscopy (fs- and ns-TA) revealed that both the singlet (S_1_) and triplet (T_1_) states contribute to the NIR-II emission. We further discuss the growth mechanism and factors for shape-control of thiolate-protected NCs, which can serve as important guidelines for rational design of new NCs with excellent functionality.

## Results and Discussion

The synthesis of **Au**_**76**_ and **Au**_**52**_ NCs is detailed in the Supporting Information. Briefly, [Au­(I)-*p*-MBT]_*x*_ polymers were obtained by reacting HAuCl_4_·4H_2_O with *p*-MBTH thiol in the presence of phenylacetylene (note: the use of phenylacetylene can affect the *x* value of the polymers and lead to a higher yield of the final products). Then, a mixture of NCs was obtained by reducing the polymers with a controlled dosage of NaBH_4_. **Au**_**52**_ was obtained after 1 h of reaction by preparative thin-layer chromatography (TLC).^32^ At the same time, another Au nanocluster with a molecular mass of 11.4 kDa was isolated by TLC (Figure S1A). A prominent absorption band at ∼400 nm and several humps were observed in the optical spectrum (Figure S1B). **Au**_**76**_ was obtained by reacting the 11.4 kDa species with excess *p*-MBTH thiol at 50 °C and subsequent TLC separation. Single crystals of **Au**_**76**_ were obtained by vapor diffusion of ethanol into a toluene/DCM (2:1) solution of the nanocluster in one month, followed by X-ray crystallography analysis (Tables S1–S7). The **Au**_**76**_ crystallizes in the orthorhombic space group *Pna*2_1_ with unit cell parameters *a* = 42.9740(8) Å, *b* = 28.1448(5) Å, and *c* = 32.5794(6) Å. The superlattice adopts a 4H structure (Figure S2).

The structure of **Au**_**76**_ consists of a fcc-type Au_68_ core anchored by four Au_2_(SR)_3_ staple motifs at the triangular {111} facets at four of the eight corners (Figure 1A), which is similar to the thiolate-protected fcc Au NCs. On the other hand, for fcc Au NCs protected by two different kinds of ligands (e.g., phosphine, halides), the {111} facets tend to be occupied by the plain ligands,^41,42^ rather than by Au-SR staples. The other facets are protected solely by bridging thiolates. If one gold atom from each dimeric staple is viewed as merging into the core,^39^ it can be viewed as a 72-atom gold box consisting of four rectangular side facets and two square-shaped top/bottom facets.

**Figure 1:**
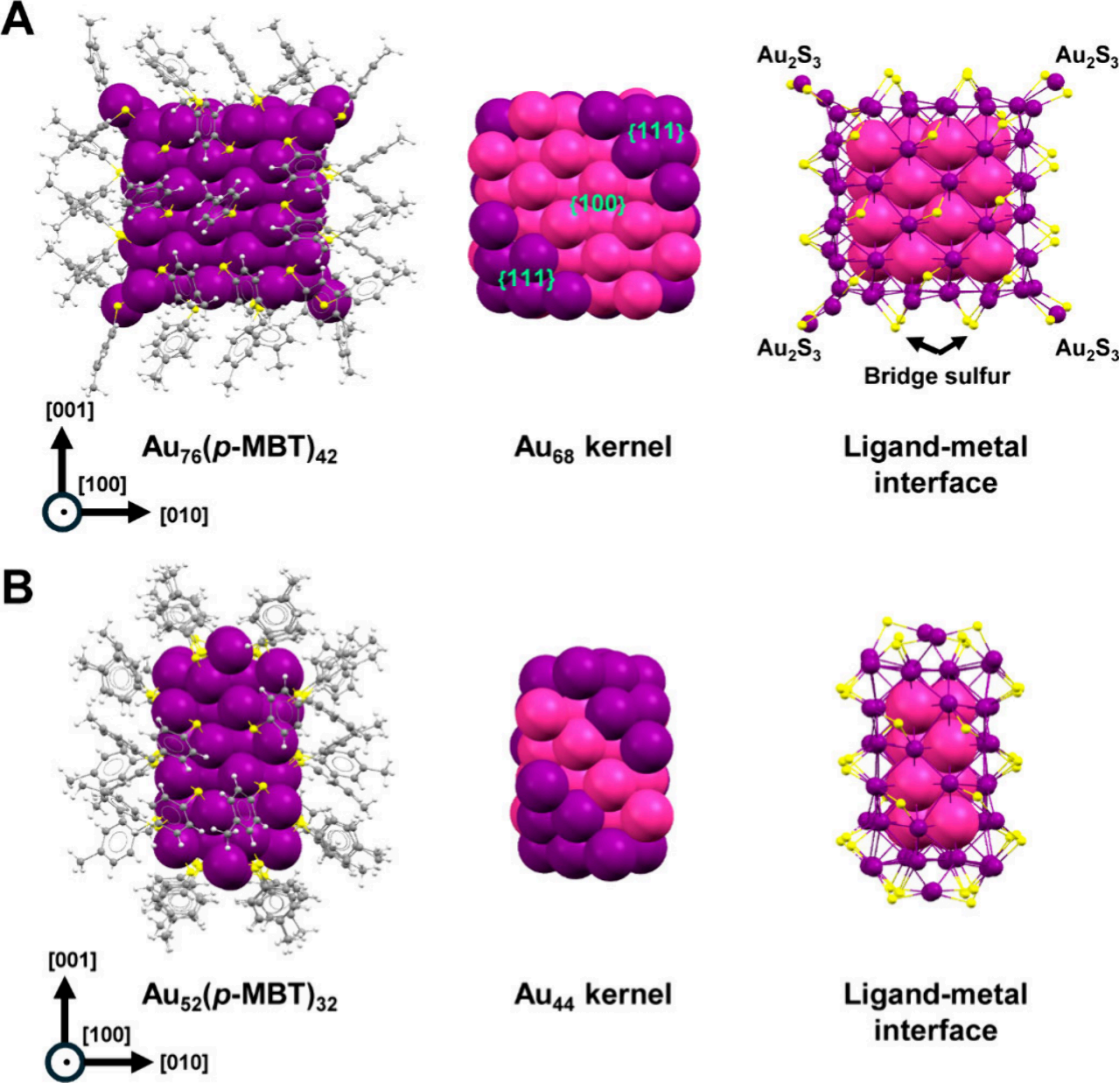
X-ray structures of **Au**_**76**_ (A) and **Au**_**52**_ (B), including the anatomy of their core and surface structures. Color code: purple and pink = Au; yellow = S; gray = C; white = H.

The **Au**_**76**_ NC is charge neutral, indicated by the observation that the cesium-adduct’s charge number equals the number of cesium ions in the adduct (Figure S3). The related structure, **Au**_**52**_, was reported previously by our group and can be viewed as a fcc box with six (001) layers (each = Au_8_) or four (010) layers (each = Au_12_), Figure 1B.^32^ Following a similar fcc pattern, a Au_66_(PET)_38_ (PET = 2-phenylethanethiolate) nanocluster, which adopts five (010) Au_12_ layers or six (001) Au_10_ layers in its core, was reported by Wu’s group.^35^ In **Au**_**76**_, the core is further extended into six (010) Au_12_ layers while maintaining the six (001) Au_10_ layers (Figure 1B), thus, the growth turns from [001] direction to [010], i.e., a 90° turn.

To understand the growth pattern of the {111} facets, the fcc structure can be viewed as an assembly of cuboctahedra via interpenetration. The Au_44_ core in the **Au**_**52**_ is made up of 8 cuboctahedra merged together along the vertical or *z*-dimension (Figure 2A, blue construct), and the further growth of **Au**_**52**_ into **Au**_**76**_ is achieved by incorporating another 8 cuboctahedra along the horizontal *x*-direction. Therefore, **Au**_**76**_ can be described as an interpenetration of two **Au**_**52**_ clusters into a single entity (Figure 2A,B). It has been proposed that the further extension of fcc-type Au nanoclusters protected by aromatic ligands (Au_28_, Au_36_, Au_44_, and Au_52_) should be a one-dimensional (1D) pattern on the {001} facet.^39,43^ However, the further increase in the aspect ratio (AR) of **Au**_**52**_ (AR: 1.67) is not favored by using aromatic thiol ligands. Recently, new classes of rod-like Au NCs were synthesized by using primary thiol or other ligands with a longer alkyl chain.^44,45^ As the thermodynamic stability of nanoclusters is determined by multiple factors, such as the nature of ligands, it is safe to say that the bulkiness of the α-carbon plays a major role in anisotropic growth. We deem that an increase in the α-carbon bulkiness is more favorable for synthesizing 2D Au NCs. On the other hand, using thiol molecules with one or more −CH_2_– units at the α position can enhance the stability of anisotropic nanoclusters, thereby promoting the growth independently along the (001) facet^46^ (Figure 2C).

**Figure 2:**
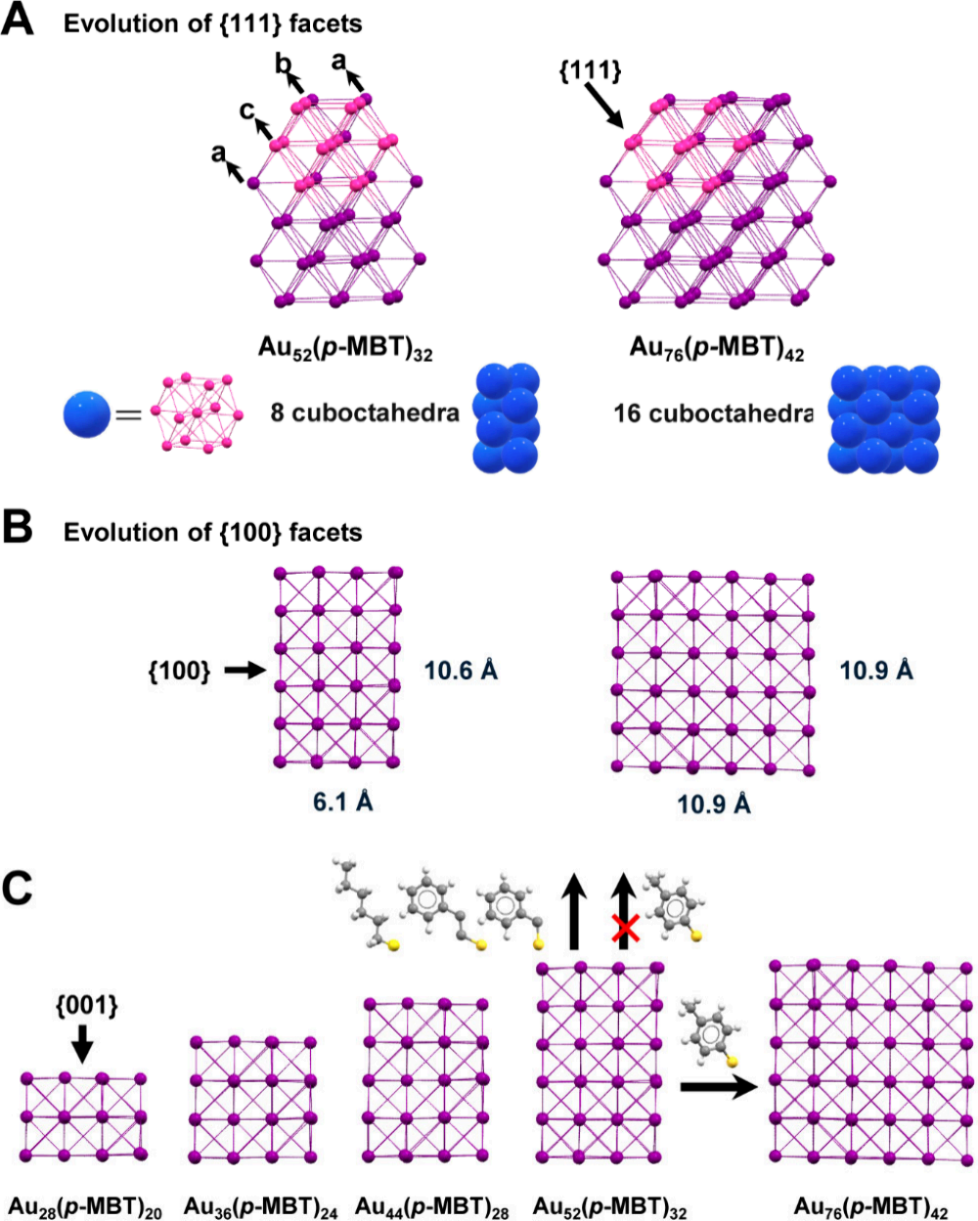
Comparison of the cores of **Au**_**76**_ and **Au**_**52**_. (A) In terms of interpenetrated cuboctahedra. (B) In terms of the fcc view. (C) Switching of fcc [001] growth (the series of Au_28_–Au_36_–Au_44_–Au_52_) to [010] growth (**Au**_**52**_ to **Au**_**76**_). Color code: purple and pink = Au; yellow = S; gray = C; and white = H.

The experimental optical absorption spectrum of **Au**_**76**_ exhibits a pronounced NIR peak at 810 nm and less distinct absorptions at 370 and 470 nm (Figure S4A). The absorption coefficient at the 810 nm peak is determined to be ε_810_ = 9.98 × 10^3^ M^–1^ cm^–1^ (Figure S4B). Time-dependent density functional theory (TDDFT) simulations were performed to analyze the electronic structure. The computed absorption spectrum of **Au**_**76**_ matches the experimental one (Figure 3A). According to the calculated Kohn–Sham (KS) energy level diagram, the computed NIR absorption peak is at 840 nm (denoted as **α**) and contains three individual electronic transitions from the HOMO and the LUMO (Figure 3 and Figure S5A). Among the three transitions, the 828 nm line mainly consists of two components, namely, HOMO → LUMO + 1 (61%) and HOMO → LUMO + 2 (36%); the 839 nm line originates from HOMO → LUMO (96%); and the 842 nm line arises from HOMO → LUMO + 1 (35%) and HOMO → LUMO + 2 (62%). Note: the LUMO and LUMO + 1 are close in energy and can be regarded as degenerate. Additionally, the visualized frontier orbitals suggest that the electron density of the HOMO is primarily localized on the inner core (Au_68_) and is mainly formed by the gold 6sp and 5d atomic orbitals (Figure 3B). On the other hand, the LUMO electron density is delocalized to the staple motifs. The distribution of these election densities and nodes exhibits some features of separated Au_4_ units^7^ that can also be found in other fcc nanoclusters.^5,6,21,47^ The 2D structure of Au_76_ leads to an in-plane-polarized transition dipole (Figure S5B), with its *x*, *y*, and *z* components being 0.8745 (in-plane), 0.0948 (in-plane), and 0.0028 (out-of-plane) in units of debye.

**Figure 3:**
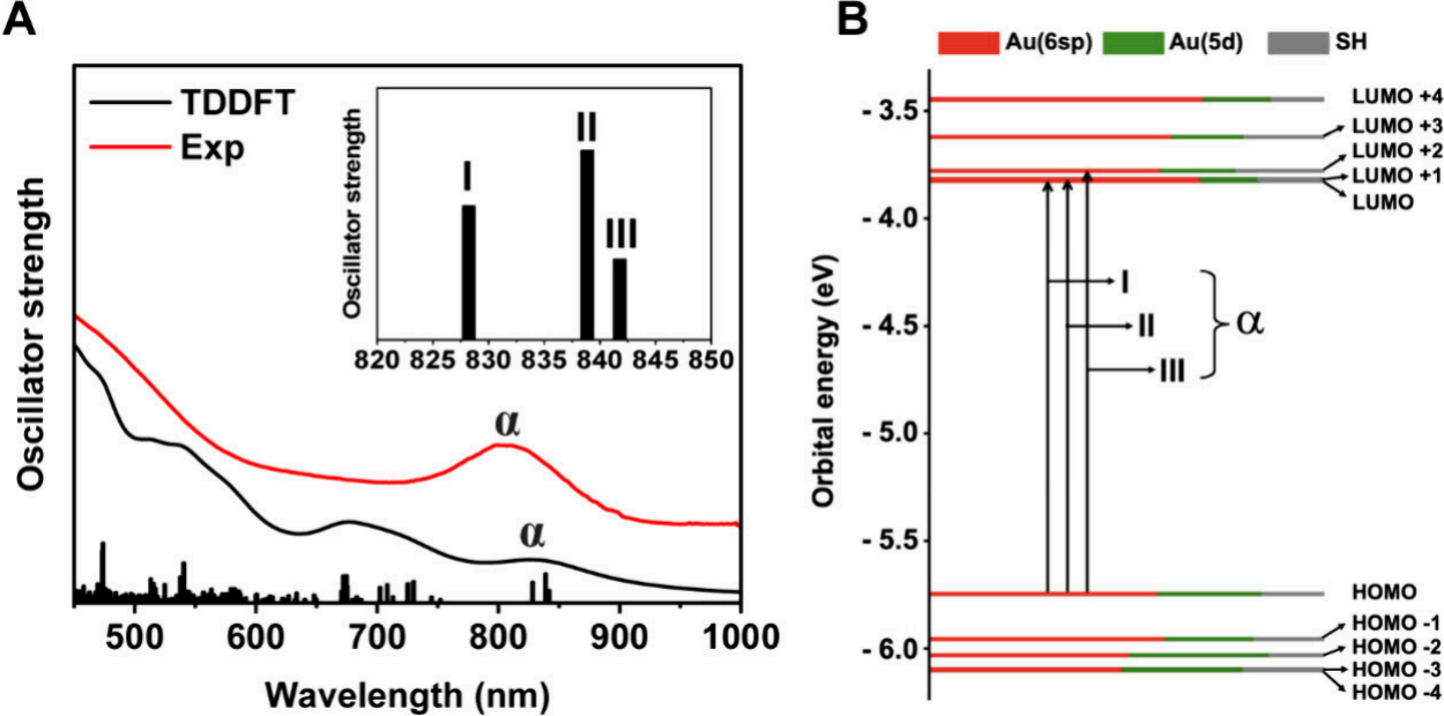
(A) Experimental (red curve) and TDDFT-simulated (black curve) absorption spectra of **Au**_**76**_. (B) Kohn–Sham (KS) orbital energy level diagram of **Au**_**76**_, with all the shown KS orbitals being predominantly composed of Au­(6sp) atomic orbitals.

A dilute non-deaerated toluene solution of **Au**_**76**_ exhibits an intense emission peak at 970 nm with an absolute PLQY of Φ_PL_ = 30.6% (Figure 4A and Figure S6) measured by an integrating sphere. The relative method gives a PLQY of Φ_PL_ = 30% using the rod-like Au_25_ as a reference (∼8% under the same conditions).^28^ The helium-deaerated solution leads to a higher PLQY (40%), indicating the participation of the spin-forbidden triplet state in the emission. Time-resolved PL obtained by time-correlated single-photon counting (TCSPC) showed that the average PL lifetime (τ_ave_) is 690 ns (components τ_1_ = 80.0 ns (14%) and τ_2_ = 790 ns (86%)) in a deaerated solution, which was shortened to 410 ns (components τ_1_ = 69.0 ns (15%) and τ_2_ = 471 ns (85%)) in the oxygen-saturated condition (Figure 4B), thus, τ_1_ is fluorescent (less) and τ_2_ is phosphorescent (dominant). We calculated the radiative decay rate (*k*_r_) by *k*_r_ = Φ_PL_·τ_ave_^–1^ and the nonradiative decay rate (*k*_nr_) by *k*_nr_ = (1 – Φ_PL_)·τ_ave_^–1^).^32,48^ The *k*_nr_ of **Au**_**76**_ is 1.01 × 10^6^ s^–1^, which is smaller than that of **Au**_**52**_ (*k*_nr_ = 1.5 × 10^6^ s^–1^).^32^ On the other hand, the *k*_r_ of **Au**_**76**_ is 4.35 × 10^5^ s^–1^, which is larger than that of **Au**_**52**_ (3.3 × 10^5^ s^–1^). Thus, both factors (i.e., *k*_nr_ and *k*_r_) lead to the enhancement of PL of **Au**_**76**_ compared to that of **Au**_**52**_. As the core of **Au**_**76**_ is similar to that of **Au**_**52**_ except the number of atomic layers, we compared the bond lengths and found that the average Au–Au bond length within the core of **Au**_**76**_ is 2.835 Å, which is shorter than that of **Au**_**52**_ (3.04 Å),^32^ suggesting that the side growth of the core results in a shortening of Au–Au bonds and, photophysically, a suppression of the nonradiative decay in the **Au**_**76**_. Among fcc-type nanoclusters, the lifetimes of Au_36_(*p*-MBT)_24_ and Au_52_(*p*-MBT)_32_ protected by the same thiolate ligand were determined to be 119 and 554 ns,^32,40^ respectively. Furthermore, the lifetimes of Au_28_, Au_36_, Au_44_, and Au_52_ protected by a different ligand, 4-*tert-*butyl-benzenethiolate (TBBT), were determined to be 180, 99, 166, and 187 ns.^38^ Thus, these reported values are all on the order of ∼10^2^ ns, implying that the carrier dynamics in the fcc type of Au NCs are similar.

**Figure 4:**
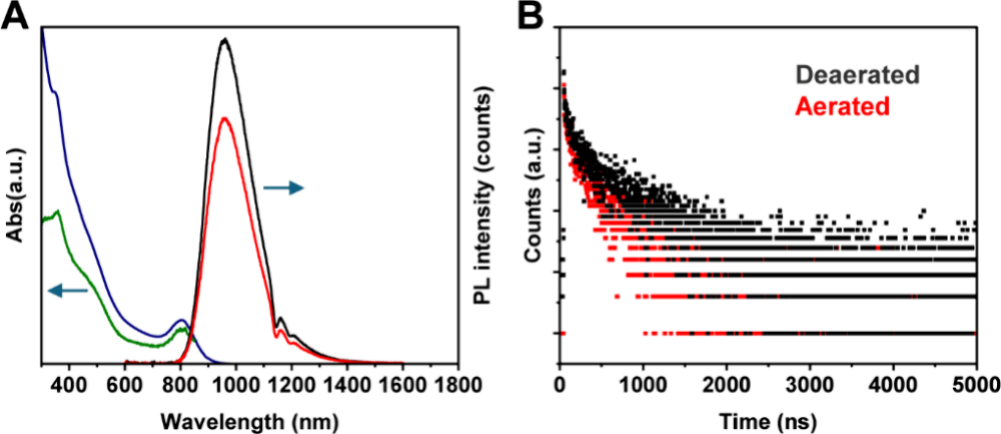
(A) Spectra of optical absorption (blue line), PL excitation (green), and emission of **Au**_**76**_ in deaerated (black) and non-deaerated (red) solution; excitation at 400 nm; optical density (OD) = 0.1. (B) PL decay profiles of **Au**_**76**_.

To elucidate the excited-state dynamics of **Au**_**76**_, we performed femtosecond- and nanosecond-transient absorption (fs- and ns-TA) measurements pumped at 400 nm and probed in the visible and NIR regions. The TA spectra of **Au**_**76**_ in the first 1 ps show very broad excited state absorption (ESA, positive signals) with peaks of 550 and 900 nm, along with ground state bleaching (GSB, negative signals) from 700 to 850 nm; the latter is consistent with the ground state absorption peak (Figure 5A, c.f. Figure 4A). A narrowed ESA (peak at 550 nm) signal and a broad ESA in the NIR region rise with the decay of the initial excited state. The ns-TA results (Figure 5B) show the final excited state with a long lifetime around 750 ns, which is regarded as a triplet state. The femtosecond kinetics of S_1_ state absorption (900 nm) and T_1_ state absorption (1300 nm) show the predecessor–successor process (Figure 5C), indicating a fast intersystem crossing process within several picoseconds. Global analysis with a sequential model (S_1_ → T_1_ → S_0_) was performed for the fs-TA data. The fast ISC process (S_1_ → T_1_) was determined to be 0.7 ps. The evolution-associated difference spectra (EADS) are shown in Figure 5D and Figure S7 for visible and near-infrared regions, respectively, in which the first species is attributed to the S_1_ state and the second one to the T_1_ state. The triplet state from ns-TA is also shown for comparison with the second EADS of fs-TA to confirm the long-lived triplet state without any other excited species.

**Figure 5:**
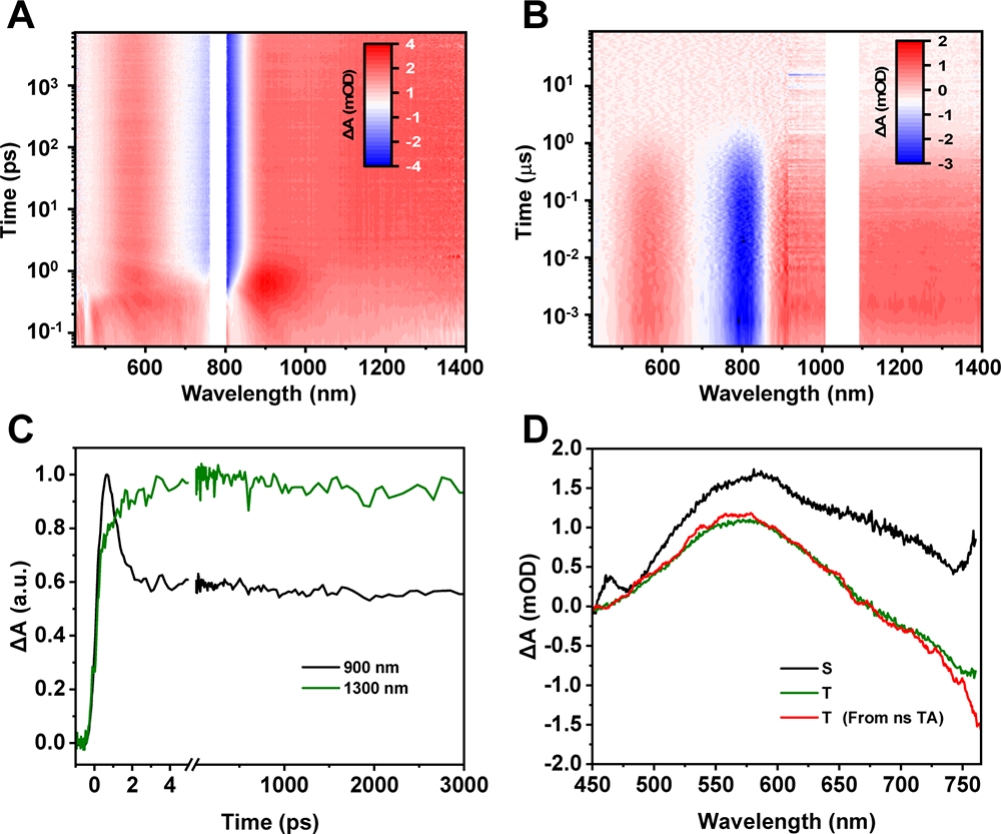
(A) fs-TA data map of **Au**_**76**_. (B) ns-TA data map of **Au**_**76**_. (C) Kinetics of the S_1_ signal at 900 nm and the T_1_ signal at 1300 nm. (D) Global analysis of fs-TA of **Au**_**76**_ (the triplet spectrum from ns-TA (red) is shown here for comparison with the fs-TA global analysis results).

Finally, it is worth noting that **Au**_**76**_ discovered in the current work differs from the Au_76_(SR)_44_ reported previously by Takano et al.^10^ While there has been no success yet in the crystallization of Au_76_(SR)_44_, it was theoretically predicted to be a fcc rod.^5,6,43^

## Conclusion

In summary, this work reports the discovery of a stable **Au**_**76**_ nanocluster with a square quantum platelet structure that can be viewed as a fusion of two **Au**_**52**_ rods (boxes) or a [010] side extension of **Au**_**52**_, as opposed to a [001] growth to longer rods. The sharp turn of 90° in the 1D growth direction is intriguing and is expected to stimulate more interest in future theoretical and experimental pursuits.^4–7,10,49^ We further found that the **Au**_**76**_ nanocluster emits at 970 nm with a solution PLQY of 30% under ambient conditions, which can be attributed to a more compact core (i.e., shorter Au–Au bond lengths) than Au_52_. The time-resolved measurements proved that phosphorescence is the dominate process in this emission. The optical properties of **Au_76_**, together with other reported NCs,^50,51^ may find applications in optoelectronics and photocatalysis.

## Supplementary Material



## Notes

Additional experimental details, materials, and methods, supporting figures S1–S7, and tables S1–S7. (PDF)
