# Supplementary material for: Au76(SC6H4‑p‑CH3)42 Square Quantum Platelet: One-Dimensional Growth of Quantum Rods Turns 90 Degrees
Source: J Am Chem Soc. 2025 Nov 10;147(46):42752–7. doi: 10.1021/jacs.5c14654 (PMC12636021; doi:10.1021/jacs.5c14654)
Supplement: Supplementary file 1 [file ja5c14654_si_001.pdf]

(Supporting Information)

$Au_{76}(SC_6H_4\text{-}p\text{-}CH_3)_{42}$ Square Quantum Platelet: One-Dimensional Growth of Quantum Rods Turns 90 Degrees

Yitong Wang[†], Avirup Sardar[†], Zhongyu Liu[†], Christopher G Gianopoulos[‡], Guiying He[†], Xiaolin Liu[§], Sihan Chen[†], Kristin Kirschbaum[‡], Abhrojyoti Mazumder[†], Mircea Cotlet[‖], De-en Jiang[§], Rongchao Jin[†*]

[†]Department of Chemistry, Carnegie Mellon University, Pittsburgh, Pennsylvania 15213, United States
[‡]Department of Chemistry and Biochemistry, University of Toledo, Toledo, Ohio 43606, United States
[§]Department of Chemical and Biomolecular Engineering, Vanderbilt University, Nashville, Tennessee 37235, United States
[‖]Center for Functional Nanomaterials, Brookhaven National Laboratory, Upton, New York 11973, United States
[*]To whom correspondence should be addressed: rongchao@andrew.cmu.edu

Experimental

Materials and Reagents

Tetrachloroauric (III) acid ($HAuCl_4{\cdot}3H_2O$, 99.999% metal basis, Aldrich), tetraoctylammonium bromide (TOAB, ≥98%, Fluka), 4-methylbenzenethiol (*p*-MBTH, 98%, Aldrich), sodium borohydride ($NaBH_4$, Aldrich). Solvents: methanol (HPLC grade, ≥99.9%, Aldrich), tetrahydrofuran (THF, HPLC grade, ≥99.5%), dichloromethane (DCM, ACS reagent, ≥99.5%, Aldrich), toluene (HPLC grade, ≥99.9%, Aldrich), hexane (ACS grade, ≥99.9%, Aldrich). All chemicals were used without further purification. Nanopure H_2O was prepared with a Barnstead NANOpure Diamond system. Thin-layer chromatography (TLC) plates were from iChromatography (silica gel, 250 µm).

Synthesis

Au_{52}(*p*-MBT)$_{32}$. A vial was charged with $HAuCl_4{\cdot}3H_2O$ (88.58 mg, 0.225 mmol). TOABr (164 mg, 0.3 mmol) was put in THF (15 ml) and added to the vial. Under stirring, the mixture becomes deep orange in 20 minutes. Then, *p*-MBTH (219 mg, 1.5 mmol) was added to the vial. After that, phenylacetylene (54.3 µL, 0.5 mmol) was added. The solution was kept stirring for 60 minutes until the color of the mixture turned to light yellow. Subsequently, $NaBH_4$ (29.6 mg, 0.78 mmol, dissolved in 5 mL of ice-cold nanopure water) was added to the vial all at once. After 60 minutes of reaction, the aqueous phase was removed, and the black organic phase was dried by rotary evaporation. The precipitates were washed with methanol five times. The size-mixed Au_x(*p*-MBT)$_y$ NCs were extracted from the precipitates with a small amount of DCM and dried under N_2 gas. The Au_{52}(*p*-MBT)$_{32}$ and intermediate Au NC was isolated by using PTLC (The PTLC was developed with a mixture of DCM and n-hexane (2:3 (v/v)).
Au_{76}(*p*-MBT)$_{42}$. 5 mg of intermediate Au NC was heated in the presence of excess of *p*-MBT (2.5 mmol added) at 50 °C for 7 hours. The resulting mixture was washed with excess methanol and further purified by PTLC using a mixture of DCM and n-hexane (2:3) as the developing solvent. Block-like Au_{76}(*p*-MBT)$_{42}$ single crystals were obtained by vapor diffusion of hexane into a toluene/DCM (1:1) solution of the nanoclusters at 4 °C in one week.

Steady-State UV-Vis-NIR Absorption Measurements

UV–Vis–NIR spectra of the NCs in solution were collected on a UV-3600 Plus spectrophotometer (Shimadzu, range: 185-3300 nm) at room temperature.

Electrospray Ionization Mass Spectrometry (ESI-MS) Analysis

ESI-MS were recorded on a Waters Q-TOF mass spectrometer equipped with Z-spray source. A dilute dichloromethane solution of the sample was prepared in advance, with CsOAc added for the formation of charged

adducts. The source temperature was kept at 70 °C. The sample solution was directly infused into the chamber at 160 μL/min. The spray voltage was kept at 4.65 kV and the cone voltage at 80 V.

Steady-State and Time-Resolved Photoluminescence and Cryogenic Measurements

Steady-state photoluminescence spectra were measured on an FLS-1000 spectrofluorometer (Edinburgh). PL lifetimes were measured by time-correlated single photon counting (TCSPC) on the same instrument. Visible PL was measured using a photomultiplier (PMT) as the detector. Near-infrared PL was measured using a wide-range InGaAs PMT detector (600-1650 nm) cooled to -80 ℃ with liquid nitrogen.

X-ray Crystallography

A specimen of $C_{294}H_{294}Au_{76}S_{42}$, approximate dimensions 0.015 mm x 0.030 mm x 0.100 mm, was used for the X-ray crystallographic analysis. Data collection was performed on a Bruker Duo diffractometer with a PHOTON II detector and IμS CuKα radiation at 250 K. The X-ray intensity data were measured (λ = 1.54178 Å). The total exposure time was 24.50 hours. The frames were integrated with the Bruker SAINT software package using a narrow-frame algorithm. The integration of the data using an orthorhombic unit cell yielded a total of 286,564 reflections to a maximum θ angle of 50.41° (1.00 Å resolution), of which 41,007 were independent (average redundancy 6.988, completeness = 99.9%, R int = 11.88%, R sig = 10.93%) and 25,320 (61.75%) were greater than 2σ(F 2). The final cell constants of a = 42.9740(8) Å, b = 28.1448(5) Å, c = 32.5794(6) Å, volume = 39404.6(12) Å 3 , are based upon the refinement of the XYZ-centroids of 9,671 reflections above 20 σ(I) with 5.842° < 2θ < 97.70°. Data were corrected for absorption effects using the Multi-Scan method (SADABS). The ratio of minimum to maximum apparent transmission was 0.250. The calculated minimum and maximum transmission coefficients (based on crystal size) are 0.0482 and 0.1925. The structure was solved in the space group Pna2 1 using Intrinsic Phasing followed by Difference-Fourier-Syntheses and then refined using the Bruker SHELXTL Software Package, with Z = 4 for the formula unit, C 294 H 294 Au 76 S 42. All Au and S atoms were refined with anisotropic thermal displacement parameters. Carbon atoms were located from the residual density maps including a disordered ligand (0.55:0.45). Eight S-C bonding distances were subjected to SADI restraints, further six 1,3 S-C distances were restrained to be similar (12 SADI restraints) to avoid tilting of the phenyl group. All 42 phenyl groups were refined as a rigid hexagon (AFIX 66). Eleven methyl carbon atoms were restrained with 22 SADI restraints to avoid tilting and twice FLAT restraints were used for the S-Phenyl moiety of the disordered ligand. Hydrogen atoms were placed in idealized positions and treated with a riding model. Final refinements revealed electron densities in voids. No chemically reasonable model of solvent molecules could be established. Therefore, PLATON SQUEEZE procedure was used, and final refinement was done with "solvent-free" data. PLATON calculated 20% voids in the unit cell. The final anisotropic full-matrix least-squares refinement on F 2 with 1751 variables converged at R_1 = 7.34%, for the observed data and wR2 = 15.31% for all data. The goodness-of-fit was 1.092. The largest peak in the final difference electron density synthesis was 1.177 e - /Å 3 and the largest hole was -1.106 e - /Å 3 with an RMS deviation of 0.211 $e^-/Å^3$. On the basis of the final model, the calculated density was 3.395 g/cm^3 and F(00), 34936 e^- .

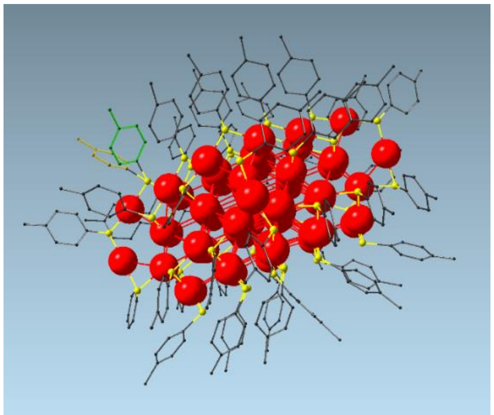

Absolute Quantum Yield Determination

The absolute PLQY of **Au_{76}(*p*-MBT)$_{42}$** in DCM solution at room temperature was determined by an integrating sphere. An example is shown in Figure S6. For such measurements, the regular sample holder is removed, and the integrating sphere is mounted in the FLS1000 spectrometer. The blank spectrum (light blue line in the Figure) was obtained by placing a blank (DCM solvent only) in the integrating sphere and collecting the emission scan (repeat 5 times) from 500 nm to 1600 nm with 525 nm excitation. The sample spectrum (yellow) was measured by replacing the blank with a dilute **Au_{76}(*p*-MBT)$_{42}$** solution in DCM (0.1 OD at 525 nm) and repeating the emission scan (5 times) from 500 nm to 1600 nm. The PLQY is calculated by:

$$\eta = L_{sample} / (E_{blank} - E_{sample})$$

where, E_{blank} is the area under the blue line between 500 and 530 nm, E_{sample} is the area under the yellow line between 500 and 530 nm, L_{sample} is the area under the yellow line between 800 and 1400 nm.

Relative Quantum Yield Determination

The relative quantum yield (Φ_S) of the sample is measured by using the $[Au_{25}(SR)_5(PPh_3)_{10}Cl_2]^{2+}$ rod-like nanocluster as the reference (QY 8%) and then calculated by the following formula:

$$\Phi_S = \Phi_R(I_S/ I_R)(1 - 10^{-AR}/ 1 - 10^{-AS})(n_S/ n_R)^2$$

where, Φ_R is the quantum yield of the reference (standard), I is the integrated PL intensity, A is the absorbance of the solution at the excitation wavelength, n is the refractive index of the solvent, and the subscripts (S and R) represent the sample and the reference, respectively.

Transient Absorption Measurements

Transient absorption measurements were carried out using a broadband pump-probe setup, which is pumped by a 1 kHz Ti:Sapphire laser system (Spitfire, Spectra-Physics). The pump wavelength of 400 nm was generated by a commercial optical parametric amplifier (TOPAS-C, Light Conversion). For femtosecond TA measurements, the supercontinuum probe light is generated by focusing the fundamental pulse (800 nm) into sapphire plate for visible and near IR probe light. The probe light is split into signal and reference beams. The pump-probe delay was controlled by a mechanical delay line. For nanosecond TA measurements, the probe light is generated with a fiber laser (Leukos), the delay times up to microseconds are controlled by an electronic delay configuration. TA measurements were performed in toluene and the optical density of solution was adjusted to ~0.3 OD (2 mm cuvette) at excitation of 400 nm. The polarization of pump and probe pulse was set to magic angle (54.7°) to measure the isotropy signal. The transient absorption spectra were further analyzed using the publicly available program Glotaran based on the statistical fitting package TIMP.

Computational Methods

All calculations were performed using the GAUSSIAN 16 package.[1] The LanL2DZ effective core potential and corresponding basis set[2] on metal atoms and 6-31G(d) on non-metal atoms are used in all calculations. We used the PBE functional[3] in geometry optimization and the B3LYP functional[4-6] in UV-vis spectrum calculations, both with D3 version of Grimme's dispersion with Becke-Johnson damping[7]. Optimized structures are confirmed as true minima by frequency calculations.

Supporting Figures:

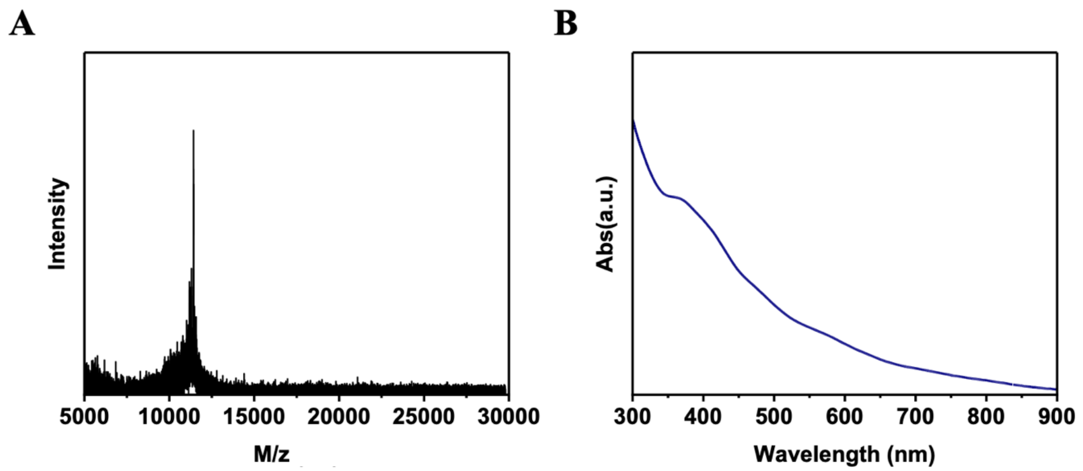

Figure S1. (A) MALDI-MS spectrum of the intermediate Au_x NC prior to conversion to Au_{76}. (B) UV-vis spectrum of this intermediate Au_x NC.

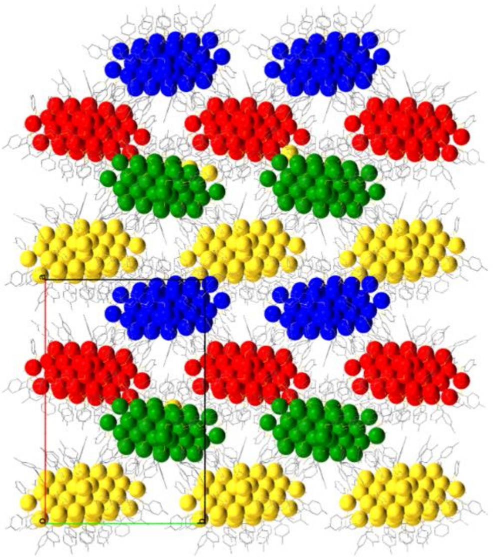

Figure S2. Arrangement of Au_{76} NCs in the superlattice.

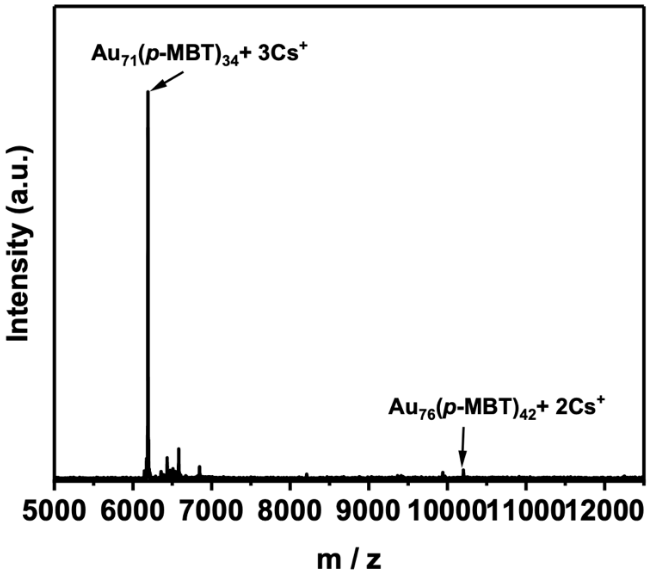

Figure S3. ESI-MS spectrum of Au_{76} in CH_2Cl_2. Note: CsOAc was added to form adducts for detection, with the intact ion observed at *m/z* = 10,203.37 in the form of $[Au_{76}(p\text{-MBT})_{42} + 2Cs]^{2+}$, albeit fragmentation occurs. The *m/z* = 6190.56 $[Au_{71}(p\text{-MBT})_{34} + 3Cs]^{3+}$ is a fragment, i.e., the intact $[Au_{76}(p\text{-MBT})_{42} + 3Cs]^{3+}$ (observed at *m/z* = 6846.92) loses one $Au(SR)_2$ and two $Au_2(p\text{-MBT})_3$ staple motifs.

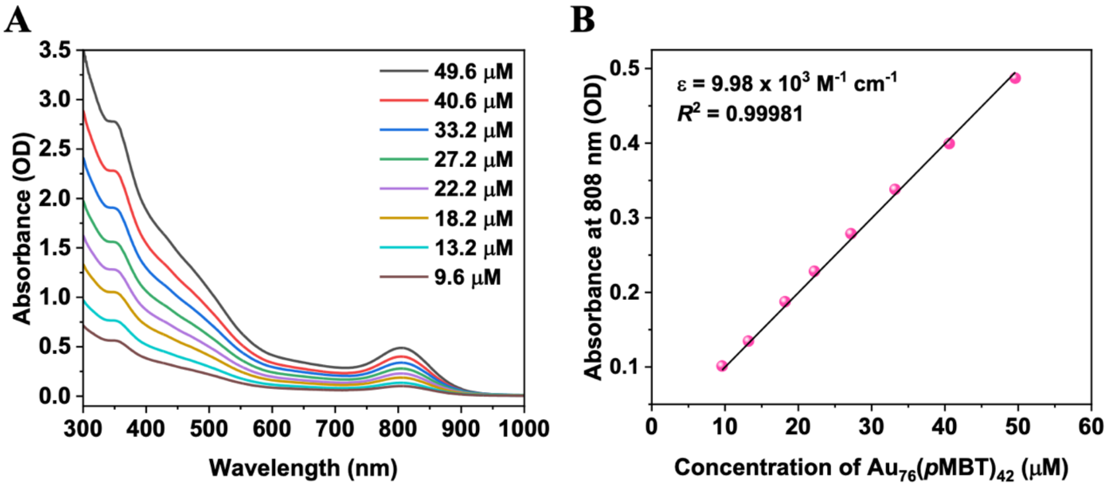

Figure S4. (A) Absorption spectra of Au_{76} with different concentrations in toluene. (B) Absorbance of Au_{76} at 810 nm vs. concentration and the linear fit gives the absorption coefficient.

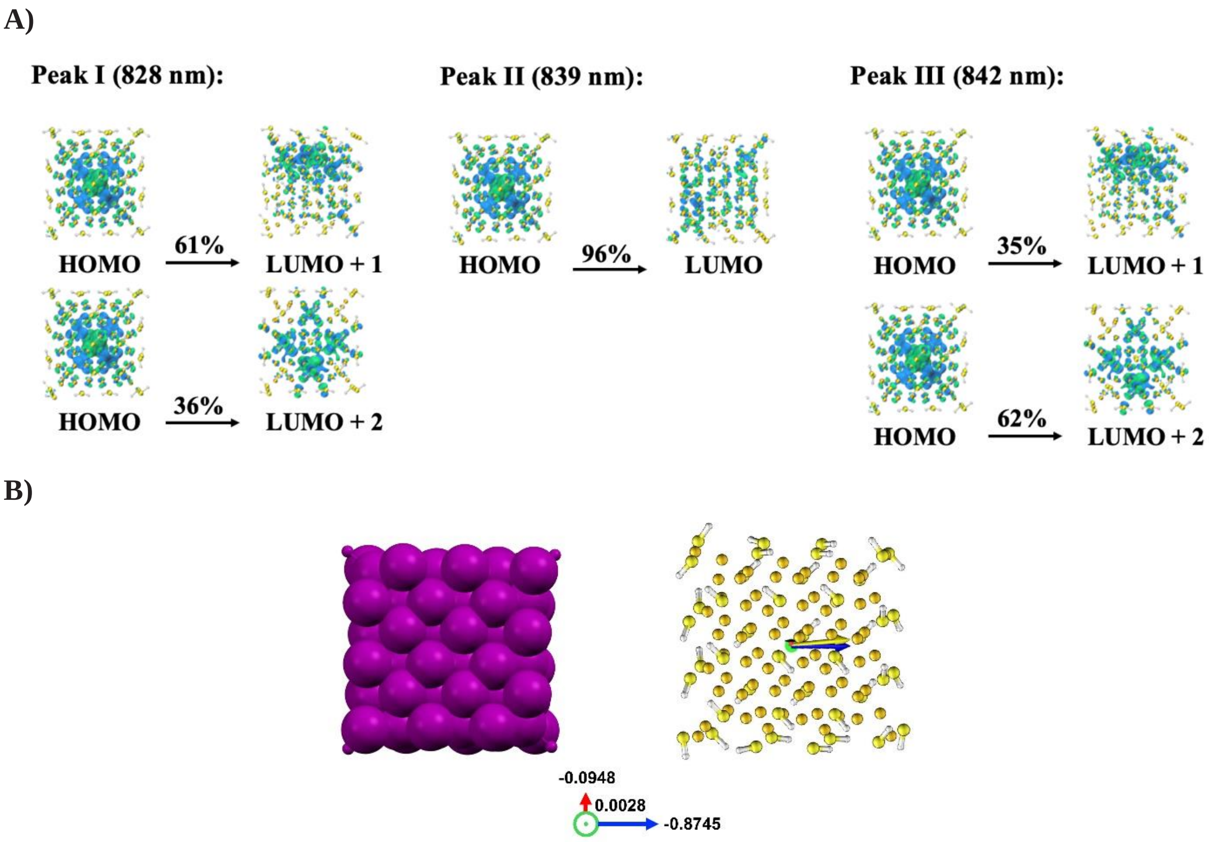

Figure S5. (A) Major component and nature of the Kohn–Sham orbitals involved in the experimental 810 nm NIR absorption peak. (B) Transition dipole (in-plane-polarized due to the 2D structure) of Au_{76}.

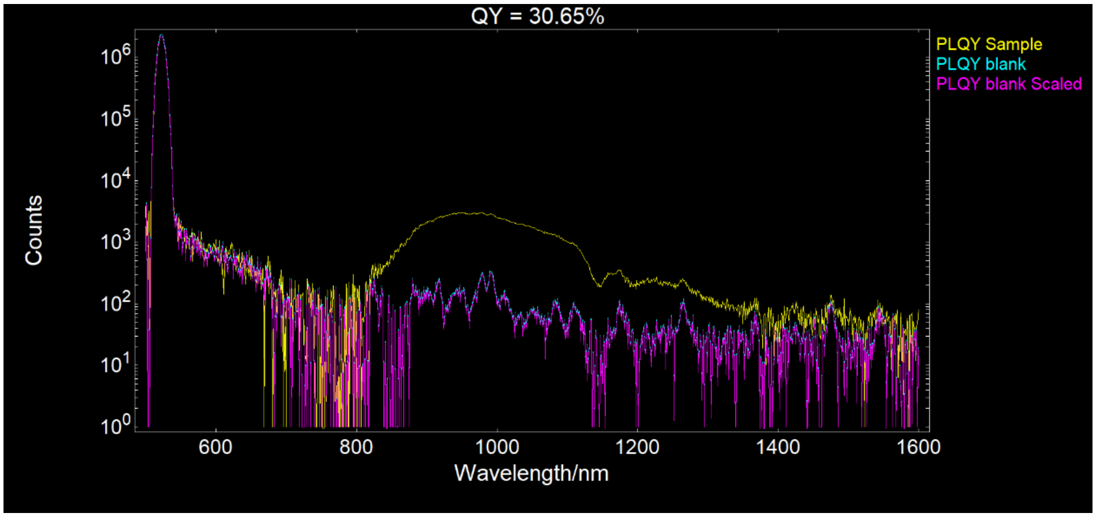

Figure S6. Determination of PLQY of Au_{76} in ambient toluene (measured by an integrating sphere); note that the y-axis in a logarithmic scale (rather than the linear scale in Figure 4A in main text), so the spectral looking is different.

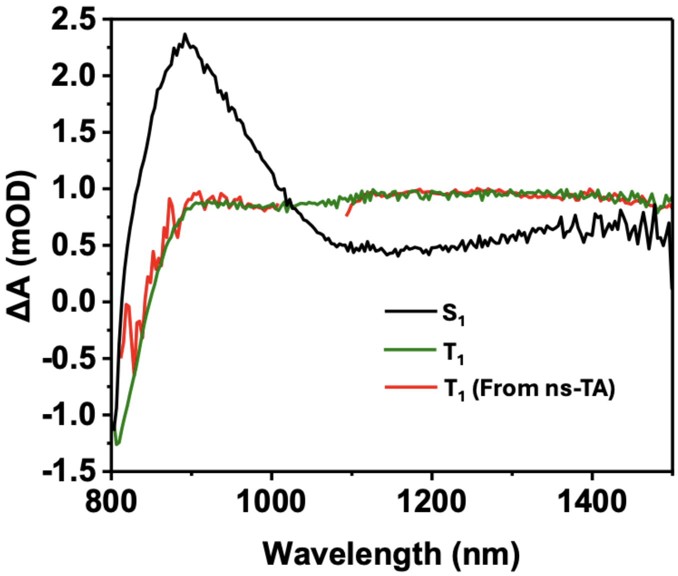

Figure S7. Global analysis of fs-TA data of Au_{76} in the NIR region.

Table S1. Sample and crystal data for $Au_{76}(S\text{-}C_6H_4\text{-}p\text{-}Me)_{42}$.		
**Chemical formula**	$C_{294}H_{294}Au_{76}S_{42}$	
**Formula weight**	20143.25 g/mol	
**Temperature**	230(2) K	
**Wavelength**	1.54178 Å	
**Crystal size**	0.015 x 0.030 x 0.100 mm	
**Crystal system**	orthorhombic	
**Space group**	P n a 21	
**Unit cell dimensions**	a = 42.9740(8) Å	α = 90°
	b = 28.1448(5) Å	β = 90°
	c = 32.5794(6) Å	γ = 90°
**Volume**	39404.6(12) $Å^3$	
**Z**	4	
**Density (calculated)**	3.395 g/cm^3	
**Absorption coefficient**	54.001 mm^{-1}	
**F(000)**	34,936	

**Table S2. Data collection and structure refinement for $Au_{76}(S\text{-}C_6H_4\text{-}p\text{-}Me)_{42}$.**		
**Theta range for data collection**	1.88 to 50.41°	
**Index ranges**	-37<=h<=42, -27<=k<=28, -32<=l<=32	
**Reflections collected**	286,564	
**Independent reflections**	41,007 [R(int) = 0.1188]	
**Coverage of independent reflections**	99.9%	
**Absorption correction**	Multi-Scan	
**Max. and min. transmission**	0.1925 and 0.0482	
**Structure solution technique**	direct methods	
**Structure solution program**	SHELXT 2018/2 (Sheldrick, 2018)	
**Refinement method**	Full-matrix least-squares on F^2	
**Refinement program**	SHELXL-2019/2 (Sheldrick, 2019)	
**Function minimized**	$\Sigma\ w(F_o^2 - F_c^2)^2$	
**Data / restraints / parameters**	41007 / 434 / 1751	
**Goodness-of-fit on F^2**	1.092	
**Δ/σ_{max}**	0.011	
**Final R indices**	25320 data; $I>2\sigma(I)$	R1 = 0.0734, wR2 = 0.1365
	all data	R1 = 0.1254, wR2 = 0.1531
**Weighting scheme**	$w=1/[\sigma^2(F_o^2)+(0.0509P)^2+0.5000P]$<br>where $P=(F_o^2+2F_c^2)/3$	
**Absolute structure parameter**	0.035(11)	
**Largest diff. peak and hole**	1.177 and -1.106 eÅ^{-3}	
**R.M.S. deviation from mean**	0.211 eÅ^{-3}	

**Table S3. Atomic coordinates and equivalent isotropic atomic displacement parameters (Å^2) for $Au_{76}(S-C_6H_4-p-Me)_{42}$.**				
U(eq) is defined as one third of the trace of the orthogonalized U_{ij} tensor.				
	**x/a**	**y/b**	**z/c**	**U(eq)**
Au1	0.64275(6)	0.21770(9)	0.62999(8)	0.0691(8)
Au2	0.68904(6)	0.17195(10)	0.68075(8)	0.0746(8)
Au3	0.59666(7)	0.15807(9)	0.67018(8)	0.0719(8)
Au4	0.63430(6)	0.21891(9)	0.72054(8)	0.0701(8)
Au5	0.67911(6)	0.27629(9)	0.68549(8)	0.0733(8)
Au6	0.59783(7)	0.26089(9)	0.57620(8)	0.0703(8)
Au7	0.68817(6)	0.27409(9)	0.58475(8)	0.0740(8)
Au8	0.65079(6)	0.21488(9)	0.53889(8)	0.0703(8)
Au9	0.69705(6)	0.16928(10)	0.59847(8)	0.0778(9)
Au10	0.60571(6)	0.15588(9)	0.57875(8)	0.0704(8)
Au11	0.55359(6)	0.19614(9)	0.61449(8)	0.0741(8)
Au12	0.63580(6)	0.32222(9)	0.62653(8)	0.0696(8)
Au13	0.58939(7)	0.26319(9)	0.66604(8)	0.0720(8)
Au14	0.62713(6)	0.32553(9)	0.71628(8)	0.0715(8)
Au15	0.67010(7)	0.28340(10)	0.76790(8)	0.0802(9)
Au16	0.58060(6)	0.26698(9)	0.75554(8)	0.0748(9)
Au17	0.61638(7)	0.32325(10)	0.81176(9)	0.0825(9)
Au18	0.57332(6)	0.37206(10)	0.74700(8)	0.0790(9)
Au19	0.62013(6)	0.42397(10)	0.71493(8)	0.0796(9)
Au20	0.58219(6)	0.36879(9)	0.66246(8)	0.0727(8)
Au21	0.67338(7)	0.38834(10)	0.67296(8)	0.0799(9)
Au22	0.64383(6)	0.32024(9)	0.53599(8)	0.0730(8)
Au23	0.69385(6)	0.27430(10)	0.50112(8)	0.0784(9)
Au24	0.60505(6)	0.25841(9)	0.48522(8)	0.0730(8)
Au25	0.59101(7)	0.36643(9)	0.57138(8)	0.0736(8)
Au26	0.68081(7)	0.38586(10)	0.59060(8)	0.0782(9)
Au27	0.63748(7)	0.41941(10)	0.53095(8)	0.0836(9)
Au28	0.69578(7)	0.38200(10)	0.49205(8)	0.0838(9)
Au29	0.60081(7)	0.36260(10)	0.48497(8)	0.0799(9)
Au30	0.62861(7)	0.42770(9)	0.62298(8)	0.0778(9)
Au31	0.58501(7)	0.47741(10)	0.57301(9)	0.0923(10)
Au32	0.62702(7)	0.52434(9)	0.62094(9)	0.0928(10)
Au33	0.57658(7)	0.47911(10)	0.65448(9)	0.0862(9)
Au34	0.62301(7)	0.22783(10)	0.81259(9)	0.0833(9)
Au35	0.64003(6)	0.11774(10)	0.72311(8)	0.0788(9)
Au36	0.66418(7)	0.01936(9)	0.63660(9)	0.0830(9)
Au37	0.65900(6)	0.11711(10)	0.54161(8)	0.0748(8)
Au38	0.64973(7)	0.31109(10)	0.43802(9)	0.0824(9)
Au39	0.65657(6)	0.21638(10)	0.44434(8)	0.0797(9)
Au40	0.65132(6)	0.11350(9)	0.63361(8)	0.0724(8)
Au41	0.67456(7)	0.49643(10)	0.68064(9)	0.0917(10)

	x/a	y/b	z/c	U(eq)
Au42	0.60829(7)	0.04802(10)	0.66964(8)	0.0818(9)
Au43	0.68428(7)	0.17601(11)	0.78147(9)	0.0963(10)
Au44	0.71143(7)	0.16639(10)	0.49730(9)	0.0857(9)
Au45	0.61264(6)	0.15144(9)	0.49380(8)	0.0742(8)
Au46	0.68422(7)	0.49216(11)	0.58111(10)	0.0992(10)
Au47	0.56802(7)	0.26645(10)	0.83784(9)	0.0858(9)
Au48	0.59003(8)	0.29235(11)	0.91848(10)	0.1125(11)
Au49	0.61507(7)	0.04583(10)	0.58749(8)	0.0795(9)
Au50	0.63984(8)	0.92477(10)	0.62754(10)	0.1066(11)
Au51	0.60671(6)	0.25253(10)	0.40208(8)	0.0795(9)
Au52	0.64560(7)	0.25725(12)	0.32982(9)	0.1064(11)
Au53	0.71400(7)	0.06410(10)	0.59055(9)	0.0866(9)
Au54	0.66953(7)	0.39247(10)	0.77370(8)	0.0835(9)
Au55	0.66708(8)	0.30038(12)	0.87615(10)	0.1079(11)
Au56	0.58565(8)	0.60565(11)	0.62116(13)	0.1341(14)
Au57	0.58879(6)	0.15919(9)	0.75536(8)	0.0757(9)
Au58	0.56119(6)	0.18279(10)	0.42881(9)	0.0827(9)
Au59	0.55099(6)	0.30560(10)	0.42839(8)	0.0811(9)
Au60	0.56239(7)	0.08740(10)	0.52505(9)	0.0844(9)
Au61	0.56300(7)	0.97245(10)	0.61851(9)	0.0985(10)
Au62	0.55957(6)	0.20443(9)	0.52212(8)	0.0749(8)
Au63	0.56168(6)	0.09969(9)	0.61726(8)	0.0795(9)
Au64	0.55307(6)	0.29998(9)	0.52016(8)	0.0765(8)
Au65	0.54442(7)	0.09643(10)	0.71122(9)	0.0921(10)
Au66	0.54056(7)	0.41568(11)	0.51517(10)	0.1024(11)
Au67	0.54548(6)	0.31308(9)	0.60955(8)	0.0757(8)
Au68	0.54239(6)	0.21003(10)	0.71067(8)	0.0791(9)
Au69	0.53545(6)	0.30545(9)	0.70456(9)	0.0803(9)
Au70	0.52510(7)	0.19912(10)	0.80009(9)	0.0875(9)
Au71	0.53986(6)	0.40937(9)	0.60819(9)	0.0854(9)
Au72	0.51698(7)	0.31543(10)	0.79447(9)	0.0905(10)
Au73	0.52153(7)	0.41971(10)	0.70118(9)	0.0892(9)
Au74	0.51902(8)	0.53155(11)	0.61161(10)	0.1079(11)
Au75	0.70520(7)	0.06837(10)	0.69570(9)	0.0838(9)
Au76	0.71440(7)	0.27355(12)	0.39516(10)	0.1059(11)
S1	0.5220(4)	0.2371(5)	0.4319(5)	0.076(5)
S2	0.5237(4)	0.1428(5)	0.5328(5)	0.084(5)
S3	0.5120(4)	0.2489(6)	0.6250(6)	0.104(6)
S4	0.5069(4)	0.1485(6)	0.6896(5)	0.086(5)
S5	0.4923(4)	0.3545(6)	0.6827(6)	0.098(6)
S6	0.4852(4)	0.2501(6)	0.7866(5)	0.100(6)
S7	0.5092(4)	0.3508(6)	0.5219(5)	0.089(5)
S8	0.7272(4)	0.3735(6)	0.5498(5)	0.081(5)
S9	0.6535(5)	0.5013(8)	0.5247(7)	0.152(9)

	x/a	y/b	z/c	U(eq)
S10	0.7096(4)	0.3775(5)	0.7282(5)	0.077(5)
S11	0.6314(4)	0.5084(6)	0.7223(5)	0.091(6)
S12	0.6280(3)	0.4070(5)	0.8164(5)	0.080(5)
S13	0.5476(4)	0.3798(6)	0.8129(5)	0.082(5)
S14	0.5512(4)	0.4861(6)	0.7195(6)	0.099(6)
S15	0.5697(3)	0.3814(5)	0.4256(5)	0.071(5)
S16	0.6678(4)	0.3904(6)	0.4321(5)	0.089(5)
S17	0.7388(4)	0.1919(5)	0.5536(5)	0.076(5)
S18	0.6903(4)	0.9438(6)	0.6381(6)	0.104(6)
S19	0.7432(4)	0.0794(6)	0.6479(5)	0.084(5)
S20	0.7021(4)	0.2786(7)	0.8272(5)	0.099(6)
S21	0.6011(3)	0.0316(5)	0.5170(5)	0.075(5)
S22	0.6829(3)	0.0403(6)	0.5375(5)	0.079(5)
S23	0.6027(4)	0.1324(5)	0.4229(5)	0.084(5)
S24	0.6845(4)	0.1437(5)	0.4408(4)	0.079(5)
S25	0.5940(4)	0.2438(6)	0.3295(5)	0.093(6)
S26	0.6990(4)	0.2523(8)	0.3319(6)	0.131(8)
S27	0.5409(4)	0.2643(7)	0.9032(5)	0.112(7)
S28	0.5770(4)	0.0337(6)	0.7297(5)	0.095(6)
S29	0.7370(5)	0.2722(11)	0.4581(6)	0.175(12)
S30	0.7166(4)	0.4951(6)	0.6380(6)	0.105(6)
S31	0.7188(4)	0.3016(7)	0.6407(5)	0.104(6)
S32	0.7237(4)	0.1921(6)	0.7357(6)	0.095(6)
S33	0.6743(4)	0.0603(6)	0.7530(5)	0.096(6)
S34	0.6494(4)	0.1581(6)	0.8344(5)	0.096(6)
S35	0.6363(5)	0.3285(7)	0.9291(6)	0.125(7)
S36	0.5650(4)	0.1480(6)	0.8207(5)	0.088(5)
S37	0.5293(4)	0.0339(6)	0.6289(6)	0.105(6)
S38	0.5910(4)	0.9065(6)	0.6021(6)	0.120(7)
S39	0.6393(5)	0.6069(6)	0.6181(8)	0.141(8)
S40	0.5328(5)	0.6062(7)	0.6302(7)	0.146(8)
S41	0.4954(5)	0.4601(6)	0.5999(8)	0.163(10)
S42	0.5700(5)	0.4803(8)	0.5030(7)	0.146(9)
C11	0.5013(8)	0.2319(17)	0.3825(10)	0.10(2)
C12	0.4847(10)	0.1906(13)	0.3742(12)	0.10(2)
C13	0.4660(9)	0.1880(13)	0.3395(14)	0.11(2)
C14	0.4639(9)	0.2266(18)	0.3130(11)	0.12(3)
C15	0.4805(10)	0.2679(13)	0.3213(13)	0.11(2)
C16	0.4992(9)	0.2705(12)	0.3561(15)	0.12(3)
C17	0.4413(19)	0.230(3)	0.276(3)	0.22(4)
C21	0.4946(7)	0.1323(15)	0.4919(10)	0.051(15)
C22	0.4895(9)	0.0895(12)	0.4716(12)	0.15(3)
C23	0.4628(10)	0.0832(12)	0.4482(11)	0.10(2)
C24	0.4411(7)	0.1196(16)	0.4452(10)	0.11(2)

	x/a	y/b	z/c	U(eq)
C25	0.4461(8)	0.1624(13)	0.4655(12)	0.083(19)
C26	0.4729(9)	0.1687(10)	0.4888(10)	0.084(19)
C27	0.4155(12)	0.106(2)	0.4136(17)	0.15(3)
C31	0.4753(7)	0.2439(10)	0.5973(13)	0.10(2)
C32	0.4613(8)	0.1997(11)	0.5928(13)	0.15(3)
C33	0.4317(9)	0.1962(17)	0.5759(14)	0.18(4)
C34	0.4160(8)	0.237(2)	0.5636(16)	0.25(5)
C35	0.4300(9)	0.2812(18)	0.5681(15)	0.27(6)
C36	0.4596(8)	0.2847(12)	0.5850(13)	0.14(3)
C37	0.3852(16)	0.231(3)	0.547(3)	0.19(4)
C41	0.4733(8)	0.1428(19)	0.7184(14)	0.11(2)
C46	0.4493(13)	0.1745(14)	0.7105(12)	0.11(2)
C44	0.4212(10)	0.1704(16)	0.7315(16)	0.13(3)
C45	0.4173(10)	0.135(2)	0.7605(15)	0.16(3)
C42	0.4414(15)	0.1030(16)	0.7684(13)	0.23(5)
C43	0.4694(11)	0.1071(15)	0.7474(16)	0.14(3)
C47	0.3841(15)	0.133(2)	0.777(2)	0.15(3)
C51	0.4587(10)	0.351(2)	0.7144(14)	0.15(3)
C52	0.4408(16)	0.3102(18)	0.7132(16)	0.21(3)
C53	0.4147(14)	0.3060(17)	0.738(2)	0.17(3)
C54	0.4064(10)	0.343(3)	0.7641(16)	0.20(3)
C55	0.4242(14)	0.3842(19)	0.7653(14)	0.14(3)
C56	0.4504(12)	0.3884(16)	0.7405(17)	0.14(2)
C57	0.3757(18)	0.334(3)	0.788(3)	0.22(4)
C61	0.4610(13)	0.242(3)	0.8260(16)	0.21(3)
C62	0.4395(15)	0.2059(19)	0.8337(16)	0.22(3)
C63	0.4196(12)	0.2093(15)	0.8671(17)	0.20(3)
C64	0.4211(11)	0.2485(16)	0.8930(15)	0.20(3)
C65	0.4425(13)	0.2844(15)	0.8853(18)	0.28(4)
C66	0.4625(11)	0.281(2)	0.852(2)	0.26(4)
C67	0.4042(18)	0.247(3)	0.9353(19)	0.21(4)
C71	0.4825(10)	0.3446(18)	0.4762(11)	0.11(2)
C72	0.4615(11)	0.3076(14)	0.4711(13)	0.14(3)
C73	0.4425(9)	0.3061(14)	0.4366(16)	0.11(2)
C74	0.4446(9)	0.3416(19)	0.4071(12)	0.16(3)
C75	0.4657(11)	0.3786(14)	0.4121(13)	0.11(2)
C76	0.4846(9)	0.3801(13)	0.4467(16)	0.13(3)
C77	0.424(2)	0.329(3)	0.365(3)	0.24(5)
C81	0.7524(10)	0.4205(14)	0.5514(16)	0.10(2)
C82	0.7732(13)	0.4184(16)	0.5840(14)	0.17(3)
C83	0.7944(10)	0.455(2)	0.5900(12)	0.17(3)
C84	0.7947(9)	0.4938(16)	0.5636(17)	0.11(2)
C85	0.7739(12)	0.4959(14)	0.5311(14)	0.19(4)
C86	0.7527(9)	0.4592(19)	0.5250(12)	0.11(2)

	x/a	y/b	z/c	U(eq)
C87	0.811(2)	0.533(3)	0.565(3)	0.26(5)
C91	0.6757(15)	0.517(2)	0.4795(19)	0.23(5)
C92	0.6659(10)	0.5093(17)	0.439(2)	0.19(4)
C93	0.6831(11)	0.5273(18)	0.4069(16)	0.21(4)
C94	0.7102(11)	0.5529(18)	0.4144(15)	0.19(4)
C95	0.7201(12)	0.560(2)	0.4545(17)	0.29(6)
C96	0.7028(16)	0.542(2)	0.4871(14)	0.33(7)
C97	0.7291(16)	0.574(3)	0.379(2)	0.22(4)
C101	0.7340(10)	0.4278(14)	0.7304(14)	0.09(2)
C102	0.7322(8)	0.4649(16)	0.7585(12)	0.09(2)
C103	0.7567(12)	0.4969(13)	0.7620(13)	0.13(3)
C104	0.7829(9)	0.4919(17)	0.7375(17)	0.19(4)
C105	0.7847(9)	0.455(2)	0.7094(14)	0.15(3)
C106	0.7603(13)	0.4228(15)	0.7059(12)	0.15(3)
C107	0.8088(19)	0.524(3)	0.740(3)	0.24(5)
C111	0.6456(11)	0.5265(14)	0.7700(10)	0.10(2)
C112	0.6744(9)	0.5457(14)	0.7797(12)	0.10(2)
C113	0.6799(7)	0.5633(12)	0.8189(14)	0.11(2)
C114	0.6565(11)	0.5618(13)	0.8484(10)	0.084(19)
C115	0.6277(9)	0.5426(14)	0.8386(12)	0.11(2)
C116	0.6222(8)	0.5249(13)	0.7995(15)	0.11(2)
C117	0.6623(13)	0.581(2)	0.8931(18)	0.12(2)
C121	0.6416(10)	0.4283(13)	0.8648(10)	0.10(2)
C122	0.6729(9)	0.4354(13)	0.8739(10)	0.10(2)
C123	0.6816(6)	0.4499(13)	0.9130(12)	0.11(2)
C124	0.6591(8)	0.4575(14)	0.9430(10)	0.12(3)
C125	0.6279(7)	0.4504(14)	0.9338(12)	0.13(3)
C126	0.6191(7)	0.4358(13)	0.8947(13)	0.11(2)
C127	0.6692(13)	0.469(3)	0.9874(13)	0.17(3)
C131	0.5268(10)	0.4319(11)	0.8182(11)	0.09(2)
C132	0.4946(10)	0.4351(12)	0.8154(12)	0.16(3)
C133	0.4800(7)	0.4789(16)	0.8197(11)	0.09(2)
C134	0.4977(10)	0.5195(11)	0.8267(11)	0.071(17)
C135	0.5299(9)	0.5163(12)	0.8296(11)	0.10(2)
C136	0.5445(7)	0.4725(16)	0.8253(11)	0.082(19)
C137	0.4768(16)	0.565(3)	0.829(2)	0.17(3)
C141	0.5246(11)	0.5389(13)	0.7225(12)	0.10(2)
C142	0.4925(11)	0.5373(14)	0.7179(12)	0.11(2)
C143	0.4748(7)	0.578(2)	0.7243(13)	0.16(3)
C144	0.4893(11)	0.6203(15)	0.7353(13)	0.11(2)
C145	0.5214(12)	0.6219(13)	0.7399(11)	0.12(3)
C146	0.5391(7)	0.5812(18)	0.7335(12)	0.10(2)
C147	0.4710(15)	0.664(2)	0.740(2)	0.14(3)
C151	0.5910(8)	0.3933(15)	0.3813(9)	0.069(16)

	x/a	y/b	z/c	U(eq)
C152	0.6078(9)	0.4352(13)	0.3771(13)	0.11(2)
C153	0.6209(9)	0.4471(12)	0.3395(17)	0.17(3)
C154	0.6172(10)	0.4170(18)	0.3060(12)	0.15(2)
C155	0.6005(10)	0.3751(15)	0.3102(10)	0.12(2)
C156	0.5874(8)	0.3632(11)	0.3478(13)	0.070(16)
C157	0.6339(16)	0.427(3)	0.2639(16)	0.19(4)
C161	0.6941(10)	0.3976(15)	0.3886(14)	0.10(2)
C162	0.7191(12)	0.4272(15)	0.3978(12)	0.13(3)
C163	0.7392(9)	0.4417(14)	0.3669(19)	0.15(3)
C164	0.7345(11)	0.4266(18)	0.3268(16)	0.18(4)
C165	0.7095(13)	0.3970(17)	0.3175(11)	0.14(3)
C166	0.6894(9)	0.3825(13)	0.3485(17)	0.12(2)
C167	0.7511(18)	0.443(3)	0.293(2)	0.20(4)
C171	0.7731(8)	0.1586(14)	0.5584(16)	0.11(2)
C172	0.7850(11)	0.1339(16)	0.5249(11)	0.09(2)
C173	0.8128(12)	0.1090(16)	0.5286(15)	0.26(5)
C174	0.8287(8)	0.1087(16)	0.5658(19)	0.17(3)
C175	0.8168(11)	0.1334(17)	0.5993(13)	0.13(3)
C176	0.7890(12)	0.1583(14)	0.5956(12)	0.11(2)
C177	0.8622(11)	0.087(2)	0.566(2)	0.16(3)
C181	0.7112(10)	0.9348(13)	0.5906(10)	0.068(17)
C182	0.6930(7)	0.9274(13)	0.5559(15)	0.11(2)
C183	0.7069(11)	0.9167(14)	0.5186(11)	0.14(3)
C184	0.7391(12)	0.9134(14)	0.5158(11)	0.12(3)
C185	0.7574(7)	0.9208(14)	0.5505(16)	0.11(2)
C186	0.7434(10)	0.9315(13)	0.5878(12)	0.12(3)
C187	0.7545(17)	0.908(3)	0.475(2)	0.18(4)
C191	0.7694(9)	0.0227(13)	0.6479(15)	0.10(2)
C192	0.7907(11)	0.0181(14)	0.6160(12)	0.11(2)
C193	0.8129(9)	0.9823(18)	0.6171(13)	0.14(3)
C194	0.8139(9)	0.9510(14)	0.6502(17)	0.15(3)
C195	0.7926(12)	0.9556(15)	0.6820(13)	0.13(3)
C196	0.7703(9)	0.9915(17)	0.6809(12)	0.11(2)
C197	0.8338(14)	0.909(2)	0.6582(19)	0.13(3)
C201	0.7295(9)	0.3202(15)	0.8265(17)	0.12(2)
C202	0.7515(13)	0.3190(15)	0.7952(13)	0.14(3)
C203	0.7761(10)	0.3511(19)	0.7952(13)	0.16(3)
C204	0.7788(9)	0.3842(15)	0.8266(17)	0.11(2)
C205	0.7569(12)	0.3854(14)	0.8580(13)	0.12(3)
C206	0.7322(10)	0.3533(18)	0.8579(13)	0.15(3)
C207	0.8086(16)	0.416(2)	0.823(2)	0.16(3)
C211	0.5849(9)	0.9739(10)	0.5123(10)	0.046(14)
C212	0.6046(6)	0.9365(15)	0.5021(11)	0.10(2)
C213	0.5926(10)	0.8911(12)	0.4966(11)	0.10(2)

	x/a	y/b	z/c	U(eq)
C214	0.5609(11)	0.8831(11)	0.5013(12)	0.11(2)
C215	0.5412(7)	0.9204(16)	0.5114(12)	0.13(3)
C216	0.5532(8)	0.9658(13)	0.5169(10)	0.087(19)
C217	0.5492(16)	0.832(2)	0.493(2)	0.17(3)
C221	0.7052(9)	0.0356(12)	0.4907(9)	0.061(16)
C222	0.7368(9)	0.0457(12)	0.4885(11)	0.11(2)
C223	0.7527(7)	0.0399(13)	0.4517(14)	0.11(2)
C224	0.7369(10)	0.0240(13)	0.4170(10)	0.10(2)
C225	0.7053(10)	0.0139(12)	0.4192(10)	0.10(2)
C226	0.6894(7)	0.0197(12)	0.4561(13)	0.09(2)
C227	0.7533(13)	0.020(2)	0.3746(13)	0.14(3)
C231	0.5922(10)	0.0729(11)	0.4158(11)	0.079(18)
C232	0.6156(7)	0.0395(17)	0.4099(12)	0.10(2)
C233	0.6080(10)	0.9921(15)	0.4032(13)	0.18(4)
C234	0.5770(12)	0.9781(10)	0.4024(12)	0.10(2)
C235	0.5536(7)	0.0115(16)	0.4083(11)	0.11(2)
C236	0.5612(9)	0.0589(14)	0.4150(11)	0.075(18)
C237	0.5695(12)	0.9260(18)	0.3921(17)	0.09(2)
C241	0.7083(10)	0.1431(14)	0.3979(11)	0.10(2)
C242	0.6927(7)	0.1292(13)	0.3626(16)	0.12(3)
C243	0.7081(11)	0.1290(13)	0.3250(12)	0.12(2)
C244	0.7392(11)	0.1428(14)	0.3229(10)	0.11(2)
C245	0.7548(7)	0.1567(13)	0.3583(15)	0.12(3)
C246	0.7394(10)	0.1569(12)	0.3958(11)	0.071(17)
C247	0.7578(17)	0.152(3)	0.285(2)	0.19(4)
C251	0.5916(9)	0.1811(10)	0.3199(11)	0.10(2)
C252	0.5609(8)	0.1658(11)	0.3166(11)	0.085(19)
C253	0.5545(6)	0.1184(12)	0.3079(11)	0.09(2)
C254	0.5788(7)	0.0864(10)	0.3026(11)	0.09(2)
C255	0.6094(6)	0.1017(12)	0.3059(11)	0.10(2)
C256	0.6158(7)	0.1490(13)	0.3145(11)	0.09(2)
C257	0.5710(12)	0.0337(13)	0.294(2)	0.14(3)
C261	0.7159(14)	0.2867(15)	0.2896(14)	0.12(2)
C262	0.6989(8)	0.2971(17)	0.254(2)	0.18(4)
C263	0.7140(14)	0.3131(16)	0.2193(14)	0.16(3)
C264	0.7461(14)	0.3188(15)	0.2194(14)	0.13(3)
C265	0.7631(8)	0.3084(16)	0.255(2)	0.15(3)
C266	0.7480(13)	0.2923(16)	0.2896(14)	0.14(3)
C267	0.7581(19)	0.347(3)	0.186(3)	0.23(5)
C271	0.5382(12)	0.2009(11)	0.9166(12)	0.10(2)
C272	0.5100(9)	0.1784(16)	0.9087(11)	0.09(2)
C273	0.5067(8)	0.1301(16)	0.9165(12)	0.13(3)
C274	0.5316(12)	0.1043(11)	0.9321(12)	0.081(19)
C275	0.5598(9)	0.1268(17)	0.9401(12)	0.14(3)

	x/a	y/b	z/c	U(eq)
C276	0.5630(8)	0.1751(18)	0.9323(13)	0.15(3)
C277	0.5311(16)	0.056(2)	0.945(2)	0.15(3)
C281	0.5539(11)	0.9825(13)	0.7243(12)	0.09(2)
C282	0.5674(7)	0.9381(18)	0.7183(12)	0.10(2)
C283	0.5487(12)	0.8979(12)	0.7159(12)	0.11(2)
C284	0.5166(12)	0.9021(16)	0.7195(14)	0.15(3)
C285	0.5031(7)	0.946(2)	0.7255(14)	0.18(4)
C286	0.5218(11)	0.9867(14)	0.7279(12)	0.11(2)
C287	0.4936(17)	0.852(3)	0.716(3)	0.20(4)
C291	0.7682(14)	0.304(2)	0.468(2)	0.23(4)
C292	0.7895(16)	0.2981(18)	0.500(2)	0.23(4)
C293	0.8100(14)	0.334(2)	0.5101(16)	0.27(4)
C294	0.8092(13)	0.3771(18)	0.4885(17)	0.26(4)
C295	0.7879(14)	0.383(2)	0.4568(17)	0.25(4)
C296	0.7674(12)	0.347(3)	0.4468(17)	0.25(4)
C297	0.8395(17)	0.409(3)	0.488(3)	0.27(5)
C301	0.7292(14)	0.5573(12)	0.633(3)	0.25(4)
C302	0.7391(16)	0.571(3)	0.672(3)	0.26(4)
C303	0.7504(14)	0.617(4)	0.678(2)	0.27(4)
C304	0.7519(13)	0.6486(18)	0.646(4)	0.22(3)
C305	0.7420(15)	0.635(2)	0.607(3)	0.30(4)
C306	0.7307(14)	0.589(3)	0.600(2)	0.31(4)
C307	0.7709(18)	0.698(3)	0.649(2)	0.20(4)
C311	0.7590(8)	0.2899(14)	0.6437(19)	0.12(3)
C312	0.7785(13)	0.2812(15)	0.6105(12)	0.15(3)
C313	0.8088(12)	0.2657(14)	0.6170(15)	0.14(3)
C314	0.8197(8)	0.2589(14)	0.6567(19)	0.12(3)
C315	0.8002(14)	0.2676(16)	0.6900(12)	0.15(3)
C316	0.7699(12)	0.2832(15)	0.6835(15)	0.15(3)
C317	0.8575(19)	0.240(3)	0.663(3)	0.22(4)
C321	0.7536(9)	0.1445(13)	0.7408(15)	0.11(2)
C322	0.7497(7)	0.1108(16)	0.7716(14)	0.16(3)
C323	0.7737(10)	0.0795(14)	0.7809(11)	0.15(3)
C324	0.8016(8)	0.0820(14)	0.7595(13)	0.16(3)
C325	0.8056(8)	0.1157(15)	0.7287(12)	0.16(3)
C326	0.7815(11)	0.1469(12)	0.7194(12)	0.13(3)
C327	0.8248(14)	0.039(2)	0.762(3)	0.23(5)
C331	0.6583(11)	0.0018(12)	0.7578(14)	0.073(18)
C332	0.6742(8)	0.9653(18)	0.7382(11)	0.11(2)
C333	0.6644(11)	0.9186(14)	0.7428(13)	0.10(2)
C334	0.6387(12)	0.9083(14)	0.7671(16)	0.13(3)
C335	0.6228(9)	0.945(2)	0.7867(15)	0.29(6)
C336	0.6326(11)	0.9916(18)	0.7821(14)	0.19(4)
C337	0.6253(19)	0.862(3)	0.775(3)	0.23(5)

	x/a	y/b	z/c	U(eq)
C341	0.6705(11)	0.1725(14)	0.8803(12)	0.080(19)
C342	0.6537(8)	0.1863(15)	0.9147(16)	0.17(3)
C343	0.6687(9)	0.1914(17)	0.9523(13)	0.20(4)
C344	0.7004(9)	0.1825(17)	0.9555(11)	0.19(4)
C345	0.7172(8)	0.1686(15)	0.9210(14)	0.14(3)
C346	0.7023(11)	0.1636(14)	0.8834(12)	0.15(3)
C347	0.7163(15)	0.183(3)	0.9981(15)	0.22(4)
C351	0.6526(16)	0.3121(19)	0.9797(16)	0.18(3)
C352	0.6843(15)	0.3163(19)	0.9874(16)	0.20(3)
C353	0.6956(10)	0.312(2)	0.0272(19)	0.25(4)
C354	0.6751(12)	0.303(2)	0.0594(15)	0.25(4)
C355	0.6434(11)	0.299(2)	0.0517(18)	0.25(4)
C356	0.6322(11)	0.3036(18)	0.012(2)	0.19(3)
C357	0.6872(19)	0.302(3)	0.1041(18)	0.28(5)
C361	0.5490(11)	0.0888(11)	0.8252(11)	0.074(18)
C362	0.5176(9)	0.0771(14)	0.8236(11)	0.09(2)
C363	0.5082(7)	0.0305(17)	0.8302(12)	0.13(3)
C364	0.5302(11)	0.9955(11)	0.8384(12)	0.12(2)
C365	0.5616(10)	0.0072(14)	0.8401(12)	0.09(2)
C366	0.5710(7)	0.0538(17)	0.8335(12)	0.12(3)
C367	0.518(2)	0.944(3)	0.842(3)	0.27(6)
C371	0.4987(9)	0.023(2)	0.5956(12)	0.08(2)
C372	0.4899(12)	0.9788(15)	0.5800(15)	0.14(3)
C373	0.4637(14)	0.9750(17)	0.5553(15)	0.19(4)
C374	0.4462(10)	0.015(3)	0.5462(14)	0.17(3)
C375	0.4550(12)	0.0592(19)	0.5617(16)	0.20(4)
C376	0.4813(13)	0.0630(14)	0.5864(14)	0.14(3)
C377	0.4159(17)	0.015(3)	0.517(2)	0.20(4)
C381	0.5755(6)	0.8532(10)	0.6274(11)	0.09(2)
C382	0.5462(6)	0.8367(12)	0.6167(11)	0.14(3)
C383	0.5357(8)	0.7933(12)	0.6317(13)	0.17(3)
C384	0.5546(10)	0.7664(11)	0.6574(14)	0.18(4)
C385	0.5840(9)	0.7829(11)	0.6680(12)	0.18(4)
C386	0.5945(7)	0.8263(11)	0.6530(10)	0.12(3)
C387	0.5379(14)	0.731(3)	0.688(2)	0.26(5)
C391	0.6461(13)	0.630(3)	0.6706(12)	0.47(5)
C392	0.6235(13)	0.623(3)	0.7006(12)	0.40(5)
C393	0.6253(16)	0.647(3)	0.7376(12)	0.33(5)
C394	0.6496(19)	0.679(3)	0.7446(16)	0.32(4)
C395	0.6722(17)	0.686(2)	0.7147(18)	0.39(5)
C396	0.6704(13)	0.661(2)	0.6777(16)	0.46(5)
C397	0.652(2)	0.697(4)	0.7894(17)	0.30(6)
C401	0.5196(17)	0.6441(17)	0.5915(15)	0.15(3)
C402	0.4874(17)	0.648(2)	0.5936(16)	0.22(3)

	x/a	y/b	z/c	U(eq)
C403	0.4721(9)	0.684(3)	0.572(2)	0.23(3)
C404	0.4891(17)	0.716(2)	0.5487(18)	0.21(3)
C405	0.5213(17)	0.7120(18)	0.5466(15)	0.17(3)
C406	0.5365(9)	0.676(2)	0.5680(17)	0.14(2)
C407	0.4777(19)	0.753(3)	0.521(3)	0.23(4)
C411_1	0.4581(7)	0.4915(13)	0.6067(17)	0.34(6)
C412_1	0.4414(15)	0.486(3)	0.643(2)	0.26(6)
C413_1	0.4133(16)	0.510(4)	0.648(3)	0.26(6)
C414_1	0.4019(11)	0.539(3)	0.617(4)	0.29(6)
C415_1	0.4186(18)	0.544(4)	0.581(4)	0.31(6)
C416_1	0.4467(17)	0.521(3)	0.576(3)	0.35(6)
C417_1	0.3704(16)	0.565(4)	0.623(5)	0.34(16)
C411_2	0.4671(7)	0.4556(14)	0.5580(10)	0.35(6)
C412_2	0.4652(19)	0.492(2)	0.529(2)	0.36(6)
C413_2	0.444(2)	0.488(3)	0.497(2)	0.37(6)
C414_2	0.4239(13)	0.449(3)	0.4947(16)	0.35(6)
C415_2	0.4258(19)	0.413(3)	0.524(2)	0.37(7)
C416_2	0.4474(18)	0.417(2)	0.555(2)	0.38(6)
C417_2	0.4010(18)	0.445(4)	0.458(2)	0.19(7)
C421	0.5417(10)	0.5233(13)	0.4834(13)	0.26(4)
C422	0.5361(11)	0.5652(13)	0.5047(14)	0.27(4)
C423	0.5134(12)	0.5966(14)	0.4910(19)	0.25(4)
C424	0.4964(10)	0.5862(18)	0.4559(19)	0.22(3)
C425	0.5021(9)	0.5443(19)	0.4345(15)	0.20(3)
C426	0.5247(10)	0.5129(16)	0.4482(12)	0.24(4)
C427	0.4793(17)	0.620(3)	0.429(2)	0.18(4)

Table S4. Bond lengths (Å) for $Au_{76}(S\text{-}C_6H_4\text{-}p\text{-}Me)_{42}$

Au1-Au6	2.877(3)	Au1-Au13	2.877(4)
Au1-Au10	2.889(4)	Au1-Au2	2.889(4)
Au1-Au9	2.891(4)	Au1-Au5	2.903(4)
Au1-Au3	2.908(4)	Au1-Au7	2.916(4)
Au1-Au40	2.958(3)	Au1-Au12	2.959(3)
Au1-Au4	2.972(3)	Au1-Au8	2.989(3)
Au2-S32	2.398(18)	Au2-Au9	2.704(4)
Au2-Au40	2.773(4)	Au2-Au35	2.944(4)
Au2-Au5	2.972(4)	Au2-Au4	2.994(4)
Au2-Au75	3.036(4)	Au2-Au43	3.290(4)
Au3-Au35	2.781(4)	Au3-Au57	2.796(4)
Au3-Au11	2.805(4)	Au3-Au63	2.816(4)
Au3-Au4	2.871(4)	Au3-Au40	2.918(4)
Au3-Au13	2.978(4)	Au3-Au10	3.005(3)
Au3-Au68	3.052(4)	Au3-Au65	3.136(4)
Au3-Au42	3.137(4)	Au4-Au5	2.761(4)
Au4-Au57	2.817(4)	Au4-Au15	2.836(4)
Au4-Au35	2.859(4)	Au4-Au13	2.904(4)
Au4-Au16	2.908(4)	Au4-Au14	3.020(4)
Au4-Au34	3.048(4)	Au4-Au43	3.164(4)
Au5-S31	2.356(17)	Au5-Au15	2.720(4)
Au5-Au14	2.814(4)	Au5-Au12	2.971(4)
Au5-Au21	3.189(4)	Au5-Au7	3.306(4)
Au6-Au64	2.871(4)	Au6-Au12	2.886(3)
Au6-Au62	2.887(4)	Au6-Au8	2.887(4)
Au6-Au67	2.898(4)	Au6-Au22	2.901(4)
Au6-Au11	2.914(4)	Au6-Au13	2.950(3)
Au6-Au10	2.976(3)	Au6-Au24	2.981(4)
Au6-Au25	2.989(4)	Au7-S31	2.377(17)
Au7-Au23	2.735(4)	Au7-Au8	2.755(4)
Au7-Au22	2.800(4)	Au7-Au12	2.959(4)
Au7-Au9	3.008(4)	Au7-Au26	3.167(4)
Au8-Au37	2.776(4)	Au8-Au23	2.781(4)
Au8-Au45	2.834(4)	Au8-Au10	2.863(4)
Au8-Au24	2.902(4)	Au8-Au22	2.982(4)
Au8-Au9	3.061(4)	Au8-Au39	3.090(4)
Au8-Au44	3.239(4)	Au9-S17	2.399(15)
Au9-Au40	2.763(4)	Au9-Au37	2.874(4)
Au9-Au53	3.060(4)	Au9-Au44	3.354(4)
Au10-Au63	2.767(4)	Au10-Au11	2.767(4)
Au10-Au45	2.786(4)	Au10-Au37	2.811(4)
Au10-Au40	2.909(4)	Au10-Au62	3.034(4)
Au10-Au49	3.136(4)	Au10-Au60	3.200(4)
Au11-S3	2.349(17)	Au11-Au63	2.738(3)

Au11-Au13	2.958(4)	Au11-Au62	3.029(4)
Au11-Au68	3.194(4)	Au11-Au67	3.314(3)
Au12-Au26	2.884(4)	Au12-Au21	2.891(4)
Au12-Au20	2.897(4)	Au12-Au13	2.897(3)
Au12-Au25	2.912(4)	Au12-Au14	2.949(4)
Au12-Au22	2.970(4)	Au12-Au30	2.987(3)
Au13-Au69	2.892(4)	Au13-Au14	2.896(4)
Au13-Au68	2.904(4)	Au13-Au16	2.942(4)
Au13-Au67	2.986(4)	Au13-Au20	2.990(4)
Au14-Au15	2.765(4)	Au14-Au19	2.787(4)
Au14-Au18	2.840(4)	Au14-Au20	2.879(4)
Au14-Au16	2.890(4)	Au14-Au21	3.011(4)
Au14-Au17	3.145(4)	Au14-Au54	3.220(4)
Au15-S20	2.376(17)	Au15-Au17	2.938(4)
Au15-Au34	2.943(4)	Au15-Au54	3.076(4)
Au15-Au43	3.115(4)	Au16-Au68	2.721(4)
Au16-Au47	2.735(4)	Au16-Au69	2.774(4)
Au16-Au34	2.827(4)	Au16-Au17	2.868(4)
Au16-Au18	2.987(4)	Au16-Au57	3.054(4)
Au16-Au72	3.308(4)	Au17-S12	2.414(15)
Au17-Au34	2.701(4)	Au17-Au47	2.756(4)
Au17-Au55	3.092(4)	Au17-Au18	3.125(4)
Au17-Au54	3.248(4)	Au18-S13	2.427(15)
Au18-Au19	2.697(4)	Au18-Au20	2.782(4)
Au18-Au69	2.842(4)	Au18-Au73	2.997(4)
Au18-Au72	3.285(4)	Au19-S11	2.437(15)
Au19-Au20	2.827(4)	Au19-Au21	2.848(4)
Au19-Au54	2.993(4)	Au19-Au30	3.020(4)
Au19-Au33	3.129(4)	Au19-Au41	3.298(4)
Au20-Au71	2.782(4)	Au20-Au67	2.814(4)
Au20-Au30	2.895(4)	Au20-Au25	2.992(4)
Au20-Au69	3.016(4)	Au20-Au33	3.125(4)
Au20-Au73	3.231(4)	Au21-S10	2.400(15)
Au21-Au26	2.703(4)	Au21-Au30	2.753(4)
Au21-Au41	3.053(4)	Au21-Au54	3.288(4)
Au22-Au23	2.754(4)	Au22-Au29	2.757(4)
Au22-Au27	2.809(4)	Au22-Au25	2.859(4)
Au22-Au24	2.922(4)	Au22-Au26	3.017(4)
Au22-Au28	3.171(4)	Au22-Au38	3.212(4)
Au23-S29	2.32(2)	Au23-Au39	2.940(4)
Au23-Au38	2.982(4)	Au23-Au28	3.047(4)
Au23-Au44	3.132(4)	Au24-Au51	2.715(4)
Au24-Au62	2.752(4)	Au24-Au64	2.767(4)
Au24-Au39	2.842(4)	Au24-Au38	2.872(4)
Au24-Au29	2.938(4)	Au24-Au45	3.041(4)

Au24-Au59	3.254(4)	Au25-Au67	2.762(4)
Au25-Au71	2.780(4)	Au25-Au27	2.819(4)
Au25-Au29	2.849(4)	Au25-Au30	2.900(4)
Au25-Au64	2.990(4)	Au25-Au31	3.134(4)
Au25-Au66	3.158(4)	Au26-S8	2.420(16)
Au26-Au30	2.744(4)	Au26-Au27	2.852(4)
Au26-Au46	3.011(4)	Au26-Au28	3.276(4)
Au27-S9	2.41(2)	Au27-Au29	2.699(4)
Au27-Au28	2.999(4)	Au27-Au30	3.031(4)
Au27-Au31	3.103(4)	Au27-Au46	3.301(4)
Au28-S16	2.306(16)	Au28-S8	2.327(15)
Au28-Au38	3.317(4)	Au29-S15	2.410(14)
Au29-Au64	2.938(4)	Au29-Au38	2.977(4)
Au29-Au66	3.147(4)	Au29-Au59	3.249(4)
Au30-Au32	2.722(4)	Au30-Au31	2.849(4)
Au30-Au33	2.854(4)	Au30-Au46	3.296(4)
Au30-Au41	3.342(4)	Au31-S42	2.37(2)
Au31-Au33	2.679(4)	Au31-Au32	2.728(4)
Au31-Au71	2.957(4)	Au31-Au66	3.197(4)
Au32-S39	2.386(18)	Au32-Au33	2.741(4)
Au32-Au56	2.898(4)	Au32-Au46	2.923(4)
Au32-Au41	2.928(4)	Au33-S14	2.390(17)
Au33-Au71	2.935(4)	Au33-Au74	3.201(4)
Au33-Au73	3.272(4)	Au34-S34	2.375(16)
Au34-Au47	2.728(4)	Au34-Au57	3.061(4)
Au34-Au43	3.176(4)	Au35-S33	2.392(16)
Au35-Au57	2.704(4)	Au35-Au42	2.957(4)
Au35-Au40	2.959(4)	Au35-Au43	3.150(4)
Au35-Au75	3.252(4)	Au36-S18	2.405(17)
Au36-Au40	2.708(4)	Au36-Au49	2.751(4)
Au36-Au42	2.753(4)	Au36-Au50	2.875(4)
Au36-Au53	2.902(4)	Au36-Au75	2.953(4)
Au37-S22	2.398(16)	Au37-Au45	2.707(4)
Au37-Au44	3.014(4)	Au37-Au40	3.017(4)
Au37-Au49	3.134(4)	Au37-Au53	3.218(4)
Au38-S16	2.373(16)	Au38-Au39	2.689(4)
Au38-Au51	2.739(4)	Au38-Au76	3.285(4)
Au39-S24	2.374(15)	Au39-Au51	2.743(4)
Au39-Au45	3.082(4)	Au39-Au44	3.242(4)
Au39-Au76	3.366(4)	Au40-Au42	2.863(4)
Au40-Au49	2.883(4)	Au40-Au75	3.327(4)
Au40-Au53	3.340(4)	Au41-S30	2.279(18)
Au41-S11	2.323(16)	Au41-Au46	3.271(4)
Au42-S28	2.407(17)	Au42-Au49	2.693(4)
Au42-Au63	3.006(4)	Au42-Au61	3.330(4)

Au42-Au65	3.350(4)	Au43-S32	2.302(17)
Au43-S34	2.340(18)	Au44-S24	2.265(16)
Au44-S17	2.294(15)	Au45-S23	2.411(16)
Au45-Au62	2.877(4)	Au45-Au60	2.991(4)
Au45-Au58	3.186(4)	Au46-S9	2.28(2)
Au46-S30	2.32(2)	Au47-S27	2.426(16)
Au47-Au48	2.886(4)	Au47-Au70	2.916(4)
Au47-Au72	2.951(4)	Au48-S35	2.260(19)
Au48-S27	2.307(19)	Au49-S21	2.408(16)
Au49-Au63	2.916(4)	Au49-Au61	3.208(4)
Au49-Au60	3.260(4)	Au50-S18	2.262(18)
Au50-S38	2.315(19)	Au51-S25	2.440(16)
Au51-Au52	2.890(4)	Au51-Au58	2.905(4)
Au51-Au59	2.950(4)	Au52-S25	2.251(17)
Au52-S26	2.300(19)	Au53-S22	2.284(16)
Au53-S19	2.290(15)	Au54-S12	2.299(15)
Au54-S10	2.312(16)	Au55-S20	2.279(17)
Au55-S35	2.31(2)	Au56-S40	2.29(2)
Au56-S39	2.31(2)	Au57-S36	2.384(17)
Au57-Au68	2.854(4)	Au57-Au65	2.971(4)
Au57-Au70	3.298(4)	Au58-S1	2.277(15)
Au58-S23	2.288(16)	Au58-Au62	3.101(4)
Au59-S15	2.283(15)	Au59-S1	2.299(15)
Au59-Au64	2.995(4)	Au60-S2	2.293(17)
Au60-S21	2.301(15)	Au60-Au63	3.024(4)
Au60-Au62	3.298(4)	Au61-S38	2.275(19)
Au61-S37	2.280(19)	Au62-S2	2.346(15)
Au62-Au64	2.704(4)	Au63-S37	2.346(16)
Au63-Au65	3.151(4)	Au64-S7	2.369(16)
Au64-Au67	2.954(4)	Au64-Au66	3.304(4)
Au65-S4	2.289(17)	Au65-S28	2.332(18)
Au65-Au68	3.198(4)	Au66-S42	2.25(2)
Au66-S7	2.280(17)	Au66-Au71	3.036(4)
Au67-S3	2.364(17)	Au67-Au71	2.721(4)
Au67-Au69	3.133(4)	Au68-S4	2.406(15)
Au68-Au69	2.710(4)	Au68-Au70	3.022(4)
Au69-S5	2.420(16)	Au69-Au72	3.048(4)
Au69-Au73	3.273(4)	Au70-S6	2.278(18)
Au70-S36	2.337(16)	Au70-Au72	3.297(4)
Au71-S41	2.401(18)	Au71-Au73	3.144(4)
Au72-S6	2.305(18)	Au72-S13	2.318(17)
Au73-S5	2.305(18)	Au73-S14	2.339(18)
Au74-S40	2.26(2)	Au74-S41	2.29(2)
Au75-S19	2.278(16)	Au75-S33	2.301(17)
Au76-S26	2.25(2)	Au76-S29	2.27(2)

S1-C11	1.84(3)	S2-C21	1.85(3)
S3-C31	1.82(2)	S4-C41	1.73(4)
S5-C51	1.78(4)	S6-C61	1.67(5)
S7-C71	1.89(4)	S8-C81	1.71(4)
S9-C91	1.81(5)	S10-C101	1.76(3)
S11-C111	1.75(3)	S12-C121	1.78(3)
S13-C131	1.72(3)	S14-C141	1.88(3)
S15-C151	1.74(3)	S16-C161	1.82(4)
S17-C171	1.76(3)	S18-C181	1.81(3)
S19-C191	1.95(3)	S20-C201	1.66(3)
S21-C211	1.77(3)	S22-C221	1.80(3)
S23-C231	1.75(3)	S24-C241	1.73(3)
S25-C251	1.79(3)	S26-C261	1.83(4)
S27-C271	1.84(3)	S28-C281	1.76(3)
S29-C291	1.65(5)	S30-C301	1.84(3)
S31-C311	1.76(4)	S32-C321	1.86(2)
S33-C331	1.79(4)	S34-C341	1.80(4)
S35-C351	1.85(5)	S36-C361	1.81(3)
S37-C371	1.73(4)	S38-C381	1.84(2)
S39-C391	1.85(2)	S40-C401	1.75(4)
S41-C411_2	1.83(2)	S41-C411_1	1.84(2)
S42-C421	1.83(2)	C11-C12	1.39000
C11-C16	1.39000	C12-C13	1.39000
C13-C14	1.39000	C14-C15	1.39000
C14-C17	1.54(8)	C15-C16	1.39000
C21-C22	1.39000	C21-C26	1.39000
C22-C23	1.39000	C23-C24	1.39000
C24-C25	1.39000	C24-C27	1.55(3)
C25-C26	1.39000	C31-C32	1.39000
C31-C36	1.39000	C32-C33	1.39000
C33-C34	1.39000	C34-C35	1.39000
C34-C37	1.43(7)	C35-C36	1.39000
C41-C46	1.39000	C41-C43	1.39000
C46-C44	1.39000	C44-C45	1.39000
C45-C42	1.39000	C45-C47	1.53(7)
C42-C43	1.39000	C51-C52	1.39000
C51-C56	1.39000	C52-C53	1.39000
C53-C54	1.39000	C54-C55	1.39000
C54-C57	1.55(8)	C55-C56	1.39000
C61-C62	1.39000	C61-C66	1.39000
C62-C63	1.39000	C63-C64	1.39000
C64-C65	1.39000	C64-C67	1.56(3)
C65-C66	1.39000	C71-C72	1.39000
C71-C76	1.39000	C72-C73	1.39000
C73-C74	1.39000	C74-C75	1.39000

C74-C77	1.68(8)	C75-C76	1.39000
C81-C82	1.39000	C81-C86	1.39000
C82-C83	1.39000	C83-C84	1.39000
C84-C87	1.33(9)	C84-C85	1.39000
C85-C86	1.39000	C91-C92	1.39000
C91-C96	1.39000	C92-C93	1.39000
C93-C94	1.39000	C94-C95	1.39000
C94-C97	1.54(3)	C95-C96	1.39000
C101-C102	1.39000	C101-C106	1.39000
C102-C103	1.39000	C103-C104	1.39000
C104-C105	1.39000	C104-C107	1.44(8)
C105-C106	1.39000	C111-C112	1.39000
C111-C116	1.39000	C112-C113	1.39000
C113-C114	1.39000	C114-C115	1.39000
C114-C117	1.58(6)	C115-C116	1.39000
C121-C122	1.39000	C121-C126	1.39000
C122-C123	1.39000	C123-C124	1.39000
C124-C125	1.39000	C124-C127	1.55(3)
C125-C126	1.39000	C131-C132	1.39000
C131-C136	1.39000	C132-C133	1.39000
C133-C134	1.39000	C134-C135	1.39000
C134-C137	1.57(7)	C135-C136	1.39000
C141-C142	1.39000	C141-C146	1.39000
C142-C143	1.39000	C143-C144	1.39000
C144-C145	1.39000	C144-C147	1.47(6)
C145-C146	1.39000	C151-C152	1.39000
C151-C156	1.39000	C152-C153	1.39000
C153-C154	1.39000	C154-C155	1.39000
C154-C157	1.57(3)	C155-C156	1.39000
C161-C162	1.39000	C161-C166	1.39000
C162-C163	1.39000	C163-C164	1.39000
C164-C165	1.39000	C164-C167	1.40(8)
C165-C166	1.39000	C171-C172	1.39000
C171-C176	1.39000	C172-C173	1.39000
C173-C174	1.39000	C174-C175	1.39000
C174-C177	1.57(3)	C175-C176	1.39000
C181-C182	1.39000	C181-C186	1.39000
C182-C183	1.39000	C183-C184	1.39000
C184-C185	1.39000	C184-C187	1.50(7)
C185-C186	1.39000	C191-C192	1.39000
C191-C196	1.39000	C192-C193	1.39000
C193-C194	1.39000	C194-C195	1.39000
C194-C197	1.49(6)	C195-C196	1.39000
C201-C202	1.39000	C201-C206	1.39000
C202-C203	1.39000	C203-C204	1.39000

C204-C205	1.39000	C204-C207	1.57(7)
C205-C206	1.39000	C211-C212	1.39000
C211-C216	1.39000	C212-C213	1.39000
C213-C214	1.39000	C214-C215	1.39000
C214-C217	1.55(7)	C215-C216	1.39000
C221-C222	1.39000	C221-C226	1.39000
C222-C223	1.39000	C223-C224	1.39000
C224-C225	1.39000	C224-C227	1.56(3)
C225-C226	1.39000	C231-C232	1.39000
C231-C236	1.39000	C232-C233	1.39000
C233-C234	1.39000	C234-C235	1.39000
C234-C237	1.54(5)	C235-C236	1.39000
C241-C242	1.39000	C241-C246	1.39000
C242-C243	1.39000	C243-C244	1.39000
C244-C245	1.39000	C244-C247	1.49(7)
C245-C246	1.39000	C251-C252	1.39000
C251-C256	1.39000	C252-C253	1.39000
C253-C254	1.39000	C254-C255	1.39000
C254-C257	1.54(3)	C255-C256	1.39000
C261-C262	1.39000	C261-C266	1.39000
C262-C263	1.39000	C263-C264	1.39000
C264-C265	1.39000	C264-C267	1.43(8)
C265-C266	1.39000	C271-C272	1.39000
C271-C276	1.39000	C272-C273	1.39000
C273-C274	1.39000	C274-C275	1.39000
C274-C277	1.43(7)	C275-C276	1.39000
C281-C282	1.39000	C281-C286	1.39000
C282-C283	1.39000	C283-C284	1.39000
C284-C285	1.39000	C284-C287	1.72(8)
C285-C286	1.39000	C291-C292	1.39000
C291-C296	1.39000	C292-C293	1.39000
C293-C294	1.39000	C294-C295	1.39000
C294-C297	1.57(3)	C295-C296	1.39000
C301-C302	1.39000	C301-C306	1.39000
C302-C303	1.39000	C303-C304	1.39000
C304-C305	1.39000	C304-C307	1.61(8)
C305-C306	1.39000	C311-C312	1.39000
C311-C316	1.39000	C312-C313	1.39000
C313-C314	1.39000	C314-C315	1.39000
C314-C317	1.72(8)	C315-C316	1.39000
C321-C322	1.39000	C321-C326	1.39000
C322-C323	1.39000	C323-C324	1.39000
C324-C325	1.39000	C324-C327	1.56(3)
C325-C326	1.39000	C331-C332	1.39000
C331-C336	1.39000	C332-C333	1.39000

C333-C334	1.39000	C334-C335	1.39000
C334-C337	1.44(8)	C335-C336	1.39000
C341-C342	1.39000	C341-C346	1.39000
C342-C343	1.39000	C343-C344	1.39000
C344-C345	1.39000	C344-C347	1.55(3)
C345-C346	1.39000	C351-C352	1.39000
C351-C356	1.39000	C352-C353	1.39000
C353-C354	1.39000	C354-C355	1.39000
C354-C357	1.55(3)	C355-C356	1.39000
C361-C362	1.39000	C361-C366	1.39000
C362-C363	1.39000	C363-C364	1.39000
C364-C365	1.39000	C364-C367	1.55(9)
C365-C366	1.39000	C371-C372	1.39000
C371-C376	1.39000	C372-C373	1.39000
C373-C374	1.39000	C374-C375	1.39000
C374-C377	1.62(8)	C375-C376	1.39000
C381-C382	1.39000	C381-C386	1.39000
C382-C383	1.39000	C383-C384	1.39000
C384-C385	1.39000	C384-C387	1.58(3)
C385-C386	1.39000	C391-C392	1.39000
C391-C396	1.39000	C392-C393	1.39000
C393-C394	1.39000	C394-C395	1.39000
C394-C397	1.55(3)	C395-C396	1.39000
C401-C402	1.39000	C401-C406	1.39000
C402-C403	1.39000	C403-C404	1.39000
C404-C405	1.39000	C404-C407	1.47(8)
C405-C406	1.39000	C411_1-C412_1	1.39000
C411_1-C416_1	1.39000	C412_1-C413_1	1.39000
C413_1-C414_1	1.39000	C414_1-C415_1	1.39000
C414_1-C417_1	1.55(2)	C415_1-C416_1	1.39000
C411_2-C412_2	1.39000	C411_2-C416_2	1.39000
C412_2-C413_2	1.39000	C413_2-C414_2	1.39000
C414_2-C415_2	1.39000	C414_2-C417_2	1.55(2)
C415_2-C416_2	1.39000	C421-C422	1.39000
C421-C426	1.39000	C422-C423	1.39000
C423-C424	1.39000	C424-C425	1.39000
C424-C427	1.50(7)	C425-C426	1.39000

**Table S5. Bond angles (°) for $Au_{76}(S\text{-}C_6H_4\text{-}p\text{-}Me)_{42}$**			
Au6-Au1-Au13	61.68(9)	Au6-Au1-Au10	62.14(9)
Au13-Au1-Au10	93.71(10)	Au6-Au1-Au2	177.32(13)
Au13-Au1-Au2	120.90(11)	Au10-Au1-Au2	116.21(11)
Au6-Au1-Au9	121.63(12)	Au13-Au1-Au9	176.57(13)
Au10-Au1-Au9	87.45(10)	Au2-Au1-Au9	55.78(9)
Au6-Au1-Au5	120.03(11)	Au13-Au1-Au5	85.53(10)
Au10-Au1-Au5	176.68(12)	Au2-Au1-Au5	61.72(9)
Au9-Au1-Au5	93.13(10)	Au6-Au1-Au3	93.50(10)
Au13-Au1-Au3	61.97(9)	Au10-Au1-Au3	62.44(9)
Au2-Au1-Au3	87.37(10)	Au9-Au1-Au3	115.96(11)
Au5-Au1-Au3	114.46(11)	Au6-Au1-Au7	84.91(10)
Au13-Au1-Au7	119.84(11)	Au10-Au1-Au7	113.86(11)
Au2-Au1-Au7	94.07(10)	Au9-Au1-Au7	62.40(9)
Au5-Au1-Au7	69.23(9)	Au3-Au1-Au7	176.26(12)
Au6-Au1-Au40	121.79(11)	Au13-Au1-Au40	121.62(11)
Au10-Au1-Au40	59.65(8)	Au2-Au1-Au40	56.61(9)
Au9-Au1-Au40	56.37(9)	Au5-Au1-Au40	118.12(11)
Au3-Au1-Au40	59.65(9)	Au7-Au1-Au40	118.45(11)
Au6-Au1-Au12	59.27(8)	Au13-Au1-Au12	59.51(8)
Au10-Au1-Au12	121.40(11)	Au2-Au1-Au12	122.30(11)
Au9-Au1-Au12	122.45(11)	Au5-Au1-Au12	60.88(9)
Au3-Au1-Au12	121.48(11)	Au7-Au1-Au12	60.47(8)
Au40-Au1-Au12	178.64(12)	Au6-Au1-Au4	121.18(11)
Au13-Au1-Au4	59.50(9)	Au10-Au1-Au4	120.86(11)
Au2-Au1-Au4	61.40(9)	Au9-Au1-Au4	117.17(11)
Au5-Au1-Au4	56.03(9)	Au3-Au1-Au4	58.43(8)
Au7-Au1-Au4	125.26(11)	Au40-Au1-Au4	89.26(10)
Au12-Au1-Au4	90.81(10)	Au6-Au1-Au8	58.92(9)
Au13-Au1-Au8	120.61(11)	Au10-Au1-Au8	58.26(9)
Au2-Au1-Au8	118.48(11)	Au9-Au1-Au8	62.71(9)
Au5-Au1-Au8	124.83(11)	Au3-Au1-Au8	120.70(11)
Au7-Au1-Au8	55.61(8)	Au40-Au1-Au8	89.93(10)
Au12-Au1-Au8	90.01(10)	Au4-Au1-Au8	179.06(13)
S32-Au2-Au9	131.9(4)	S32-Au2-Au40	156.5(4)
Au9-Au2-Au40	60.58(10)	S32-Au2-Au1	138.6(4)
Au9-Au2-Au1	62.14(9)	Au40-Au2-Au1	62.94(9)
S32-Au2-Au35	102.5(4)	Au9-Au2-Au35	122.78(12)
Au40-Au2-Au35	62.24(9)	Au1-Au2-Au35	90.39(10)
S32-Au2-Au5	79.5(4)	Au9-Au2-Au5	95.58(11)
Au40-Au2-Au5	122.08(11)	Au1-Au2-Au5	59.37(8)
Au35-Au2-Au5	112.65(11)	S32-Au2-Au4	93.5(4)
Au9-Au2-Au4	122.80(12)	Au40-Au2-Au4	92.43(10)
Au1-Au2-Au4	60.67(9)	Au35-Au2-Au4	57.56(8)
Au5-Au2-Au4	55.13(8)	S32-Au2-Au75	88.0(4)

Au9-Au2-Au75	95.92(11)	Au40-Au2-Au75	69.70(10)
Au1-Au2-Au75	132.62(12)	Au35-Au2-Au75	65.85(9)
Au5-Au2-Au75	166.76(12)	Au4-Au2-Au75	122.29(11)
S32-Au2-Au43	44.4(4)	Au9-Au2-Au43	176.23(13)
Au40-Au2-Au43	122.46(12)	Au1-Au2-Au43	120.84(11)
Au35-Au2-Au43	60.40(9)	Au5-Au2-Au43	84.54(10)
Au4-Au2-Au43	60.25(9)	Au75-Au2-Au43	83.54(10)
Au35-Au3-Au57	58.01(9)	Au35-Au3-Au11	177.78(13)
Au57-Au3-Au11	123.92(12)	Au35-Au3-Au63	119.94(12)
Au57-Au3-Au63	123.35(12)	Au11-Au3-Au63	58.31(9)
Au35-Au3-Au4	60.75(9)	Au57-Au3-Au4	59.61(9)
Au11-Au3-Au4	120.91(11)	Au63-Au3-Au4	177.01(13)
Au35-Au3-Au1	93.34(10)	Au57-Au3-Au1	121.52(12)
Au11-Au3-Au1	86.43(10)	Au63-Au3-Au1	115.13(11)
Au4-Au3-Au1	61.91(9)	Au35-Au3-Au40	62.50(9)
Au57-Au3-Au40	120.50(12)	Au11-Au3-Au40	115.56(11)
Au63-Au3-Au40	85.92(10)	Au4-Au3-Au40	92.07(10)
Au1-Au3-Au40	61.03(9)	Au35-Au3-Au13	120.25(12)
Au57-Au3-Au13	91.21(10)	Au11-Au3-Au13	61.43(9)
Au63-Au3-Au13	119.72(12)	Au4-Au3-Au13	59.50(9)
Au1-Au3-Au13	58.51(8)	Au40-Au3-Au13	119.54(11)
Au35-Au3-Au10	121.30(12)	Au57-Au3-Au10	179.28(14)
Au11-Au3-Au10	56.76(9)	Au63-Au3-Au10	56.65(9)
Au4-Au3-Au10	120.39(11)	Au1-Au3-Au10	58.48(9)
Au40-Au3-Au10	58.81(9)	Au13-Au3-Au10	89.37(10)
Au35-Au3-Au68	116.08(11)	Au57-Au3-Au68	58.22(9)
Au11-Au3-Au68	65.94(9)	Au63-Au3-Au68	97.84(11)
Au4-Au3-Au68	84.13(10)	Au1-Au3-Au68	116.01(11)
Au40-Au3-Au68	176.08(12)	Au13-Au3-Au68	57.55(8)
Au10-Au3-Au68	122.49(11)	Au35-Au3-Au65	89.41(10)
Au57-Au3-Au65	59.78(9)	Au11-Au3-Au65	90.85(10)
Au63-Au3-Au65	63.66(9)	Au4-Au3-Au65	119.32(11)
Au1-Au3-Au65	177.22(12)	Au40-Au3-Au65	120.84(11)
Au13-Au3-Au65	119.59(11)	Au10-Au3-Au65	120.26(11)
Au68-Au3-Au65	62.22(9)	Au35-Au3-Au42	59.59(9)
Au57-Au3-Au42	92.06(10)	Au11-Au3-Au42	118.60(11)
Au63-Au3-Au42	60.37(9)	Au4-Au3-Au42	120.15(11)
Au1-Au3-Au42	117.31(11)	Au40-Au3-Au42	56.29(8)
Au13-Au3-Au42	175.71(12)	Au10-Au3-Au42	87.34(10)
Au68-Au3-Au42	126.67(11)	Au65-Au3-Au42	64.56(9)
Au5-Au4-Au57	179.07(13)	Au5-Au4-Au15	58.14(9)
Au57-Au4-Au15	122.65(12)	Au5-Au4-Au35	122.32(12)
Au57-Au4-Au35	56.90(9)	Au15-Au4-Au35	125.08(12)
Au5-Au4-Au3	120.37(12)	Au57-Au4-Au3	58.87(9)
Au15-Au4-Au3	176.81(13)	Au35-Au4-Au3	58.08(9)

Au5-Au4-Au13	87.68(10)	Au57-Au4-Au13	92.35(10)
Au15-Au4-Au13	114.75(11)	Au35-Au4-Au13	120.17(11)
Au3-Au4-Au13	62.10(9)	Au5-Au4-Au16	116.33(11)
Au57-Au4-Au16	64.46(9)	Au15-Au4-Au16	85.38(10)
Au35-Au4-Au16	121.35(11)	Au3-Au4-Au16	93.12(10)
Au13-Au4-Au16	60.83(9)	Au5-Au4-Au1	60.72(9)
Au57-Au4-Au1	118.53(11)	Au15-Au4-Au1	118.75(11)
Au35-Au4-Au1	90.41(10)	Au3-Au4-Au1	59.66(8)
Au13-Au4-Au1	58.62(8)	Au16-Au4-Au1	119.44(11)
Au5-Au4-Au2	62.03(9)	Au57-Au4-Au2	117.16(11)
Au15-Au4-Au2	95.27(11)	Au35-Au4-Au2	60.35(9)
Au3-Au4-Au2	86.10(10)	Au13-Au4-Au2	116.55(11)
Au16-Au4-Au2	177.25(12)	Au1-Au4-Au2	57.93(8)
Au5-Au4-Au14	58.04(9)	Au57-Au4-Au14	122.72(11)
Au15-Au4-Au14	56.24(9)	Au35-Au4-Au14	178.68(13)
Au3-Au4-Au14	120.60(11)	Au13-Au4-Au14	58.50(8)
Au16-Au4-Au14	58.31(8)	Au1-Au4-Au14	88.75(10)
Au2-Au4-Au14	119.93(11)	Au5-Au4-Au34	118.01(11)
Au57-Au4-Au34	62.78(9)	Au15-Au4-Au34	59.89(9)
Au35-Au4-Au34	93.84(10)	Au3-Au4-Au34	121.46(11)
Au13-Au4-Au34	117.42(11)	Au16-Au4-Au34	56.61(9)
Au1-Au4-Au34	175.39(12)	Au2-Au4-Au34	125.98(11)
Au14-Au4-Au34	86.97(10)	Au5-Au4-Au43	90.52(10)
Au57-Au4-Au43	89.49(11)	Au15-Au4-Au43	62.26(9)
Au35-Au4-Au43	62.82(9)	Au3-Au4-Au43	120.90(11)
Au13-Au4-Au43	177.01(12)	Au16-Au4-Au43	118.05(11)
Au1-Au4-Au43	122.36(11)	Au2-Au4-Au43	64.52(9)
Au14-Au4-Au43	118.50(11)	Au34-Au4-Au43	61.46(9)
S31-Au5-Au15	133.8(4)	S31-Au5-Au4	159.8(5)
Au15-Au5-Au4	62.32(10)	S31-Au5-Au14	130.3(5)
Au15-Au5-Au14	59.93(10)	Au4-Au5-Au14	65.60(9)
S31-Au5-Au1	100.1(4)	Au15-Au5-Au1	125.44(12)
Au4-Au5-Au1	63.25(9)	Au14-Au5-Au1	94.28(10)
S31-Au5-Au12	85.5(4)	Au15-Au5-Au12	121.14(12)
Au4-Au5-Au12	94.88(10)	Au14-Au5-Au12	61.23(9)
Au1-Au5-Au12	60.49(8)	S31-Au5-Au2	99.4(5)
Au15-Au5-Au2	98.30(11)	Au4-Au5-Au2	62.84(9)
Au14-Au5-Au2	128.27(12)	Au1-Au5-Au2	58.91(9)
Au12-Au5-Au2	119.10(11)	S31-Au5-Au21	71.2(5)
Au15-Au5-Au21	92.45(10)	Au4-Au5-Au21	125.28(11)
Au14-Au5-Au21	59.82(9)	Au1-Au5-Au21	116.12(11)
Au12-Au5-Au21	55.84(8)	Au2-Au5-Au21	168.99(12)
S31-Au5-Au7	46.0(4)	Au15-Au5-Au7	176.54(13)
Au4-Au5-Au7	118.81(11)	Au14-Au5-Au7	117.18(11)
Au1-Au5-Au7	55.56(8)	Au12-Au5-Au7	55.95(8)

Au2-Au5-Au7	85.01(9)	Au21-Au5-Au7	84.29(9)
Au64-Au6-Au1	177.27(12)	Au64-Au6-Au12	120.72(11)
Au1-Au6-Au12	61.79(9)	Au64-Au6-Au62	56.03(9)
Au1-Au6-Au62	121.43(11)	Au12-Au6-Au62	176.35(12)
Au64-Au6-Au8	115.61(11)	Au1-Au6-Au8	62.48(9)
Au12-Au6-Au8	93.54(10)	Au62-Au6-Au8	86.86(10)
Au64-Au6-Au67	61.59(9)	Au1-Au6-Au67	120.44(11)
Au12-Au6-Au67	85.56(10)	Au62-Au6-Au67	93.77(10)
Au8-Au6-Au67	175.68(13)	Au64-Au6-Au22	87.06(10)
Au1-Au6-Au22	93.50(10)	Au12-Au6-Au22	61.76(9)
Au62-Au6-Au22	115.44(11)	Au8-Au6-Au22	62.02(9)
Au67-Au6-Au22	113.98(11)	Au64-Au6-Au11	94.29(11)
Au1-Au6-Au11	84.99(10)	Au12-Au6-Au11	119.97(11)
Au62-Au6-Au11	62.96(9)	Au8-Au6-Au11	114.46(11)
Au67-Au6-Au11	69.52(9)	Au22-Au6-Au11	176.44(12)
Au64-Au6-Au13	122.69(12)	Au1-Au6-Au13	59.16(9)
Au12-Au6-Au13	59.52(9)	Au62-Au6-Au13	123.20(12)
Au8-Au6-Au13	121.64(11)	Au67-Au6-Au13	61.41(9)
Au22-Au6-Au13	121.28(11)	Au11-Au6-Au13	60.58(9)
Au64-Au6-Au10	118.33(11)	Au1-Au6-Au10	59.14(8)
Au12-Au6-Au10	120.92(11)	Au62-Au6-Au10	62.30(9)
Au8-Au6-Au10	58.44(9)	Au67-Au6-Au10	125.54(11)
Au22-Au6-Au10	120.46(11)	Au11-Au6-Au10	56.03(8)
Au13-Au6-Au10	90.47(10)	Au64-Au6-Au24	56.39(9)
Au1-Au6-Au24	121.73(11)	Au12-Au6-Au24	121.33(11)
Au62-Au6-Au24	55.92(9)	Au8-Au6-Au24	59.25(9)
Au67-Au6-Au24	117.74(11)	Au22-Au6-Au24	59.57(9)
Au11-Au6-Au24	118.62(11)	Au13-Au6-Au24	178.91(13)
Au10-Au6-Au24	89.58(10)	Au64-Au6-Au25	61.32(9)
Au1-Au6-Au25	121.18(11)	Au12-Au6-Au25	59.40(8)
Au62-Au6-Au25	117.33(11)	Au8-Au6-Au25	120.06(11)
Au67-Au6-Au25	55.93(9)	Au22-Au6-Au25	58.05(9)
Au11-Au6-Au25	125.45(11)	Au13-Au6-Au25	91.04(10)
Au10-Au6-Au25	178.32(13)	Au24-Au6-Au25	88.92(10)
S31-Au7-Au23	135.5(4)	S31-Au7-Au8	159.3(5)
Au23-Au7-Au8	60.87(9)	S31-Au7-Au22	131.3(5)
Au23-Au7-Au22	59.65(9)	Au8-Au7-Au22	64.92(9)
S31-Au7-Au1	99.2(4)	Au23-Au7-Au1	124.36(12)
Au8-Au7-Au1	63.55(9)	Au22-Au7-Au1	94.80(10)
S31-Au7-Au12	85.4(4)	Au23-Au7-Au12	121.68(12)
Au8-Au7-Au12	94.75(11)	Au22-Au7-Au12	62.03(9)
Au1-Au7-Au12	60.49(8)	S31-Au7-Au9	97.8(5)
Au23-Au7-Au9	97.98(11)	Au8-Au7-Au9	63.98(9)
Au22-Au7-Au9	128.71(12)	Au1-Au7-Au9	58.39(8)
Au12-Au7-Au9	118.49(11)	S31-Au7-Au26	71.7(5)

Au23-Au7-Au26	93.82(11)	Au8-Au7-Au26	125.11(12)
Au22-Au7-Au26	60.36(9)	Au1-Au7-Au26	116.32(11)
Au12-Au7-Au26	56.06(8)	Au9-Au7-Au26	167.89(12)
S31-Au7-Au5	45.4(4)	Au23-Au7-Au5	177.96(13)
Au8-Au7-Au5	118.75(11)	Au22-Au7-Au5	118.31(11)
Au1-Au7-Au5	55.21(8)	Au12-Au7-Au5	56.29(8)
Au9-Au7-Au5	83.44(9)	Au26-Au7-Au5	84.83(9)
Au7-Au8-Au37	120.54(12)	Au7-Au8-Au23	59.21(9)
Au37-Au8-Au23	121.71(12)	Au7-Au8-Au45	177.99(13)
Au37-Au8-Au45	57.69(9)	Au23-Au8-Au45	122.31(12)
Au7-Au8-Au10	119.96(11)	Au37-Au8-Au10	59.77(9)
Au23-Au8-Au10	178.48(13)	Au45-Au8-Au10	58.55(9)
Au7-Au8-Au6	87.71(10)	Au37-Au8-Au6	122.10(12)
Au23-Au8-Au6	116.18(12)	Au45-Au8-Au6	92.57(10)
Au10-Au8-Au6	62.33(9)	Au7-Au8-Au24	117.80(12)
Au37-Au8-Au24	121.60(12)	Au23-Au8-Au24	86.00(10)
Au45-Au8-Au24	64.02(9)	Au10-Au8-Au24	93.43(10)
Au6-Au8-Au24	61.99(9)	Au7-Au8-Au22	58.27(9)
Au37-Au8-Au22	178.45(13)	Au23-Au8-Au22	56.96(9)
Au45-Au8-Au22	123.51(11)	Au10-Au8-Au22	121.55(11)
Au6-Au8-Au22	59.22(9)	Au24-Au8-Au22	59.55(9)
Au7-Au8-Au1	60.85(9)	Au37-Au8-Au1	90.53(10)
Au23-Au8-Au1	120.01(11)	Au45-Au8-Au1	117.67(11)
Au10-Au8-Au1	59.12(8)	Au6-Au8-Au1	58.59(8)
Au24-Au8-Au1	120.58(11)	Au22-Au8-Au1	89.62(9)
Au7-Au8-Au9	62.03(9)	Au37-Au8-Au9	58.76(9)
Au23-Au8-Au9	95.77(11)	Au45-Au8-Au9	116.11(11)
Au10-Au8-Au9	84.75(10)	Au6-Au8-Au9	115.66(11)
Au24-Au8-Au9	177.58(12)	Au22-Au8-Au9	120.15(11)
Au1-Au8-Au9	57.07(8)	Au7-Au8-Au39	119.03(12)
Au37-Au8-Au39	92.02(10)	Au23-Au8-Au39	59.83(9)
Au45-Au8-Au39	62.52(9)	Au10-Au8-Au39	120.96(11)
Au6-Au8-Au39	118.49(11)	Au24-Au8-Au39	56.51(9)
Au22-Au8-Au39	87.88(10)	Au1-Au8-Au39	176.94(12)
Au9-Au8-Au39	125.85(11)	Au7-Au8-Au44	90.72(10)
Au37-Au8-Au44	59.55(9)	Au23-Au8-Au44	62.16(9)
Au45-Au8-Au44	89.03(10)	Au10-Au8-Au44	119.32(11)
Au6-Au8-Au44	178.18(12)	Au24-Au8-Au44	118.09(11)
Au22-Au8-Au44	119.12(11)	Au1-Au8-Au44	121.30(11)
Au9-Au8-Au44	64.28(9)	Au39-Au8-Au44	61.58(9)
S17-Au9-Au2	133.8(4)	S17-Au9-Au40	159.1(4)
Au2-Au9-Au40	60.96(10)	S17-Au9-Au37	99.7(4)
Au2-Au9-Au37	125.51(13)	Au40-Au9-Au37	64.67(10)
S17-Au9-Au1	134.0(4)	Au2-Au9-Au1	62.09(9)
Au40-Au9-Au1	63.04(9)	Au37-Au9-Au1	90.61(11)

S17-Au9-Au7	75.2(4)	Au2-Au9-Au7	95.97(11)
Au40-Au9-Au7	121.91(12)	Au37-Au9-Au7	109.46(11)
Au1-Au9-Au7	59.21(9)	S17-Au9-Au53	91.6(4)
Au2-Au9-Au53	98.10(11)	Au40-Au9-Au53	69.80(10)
Au37-Au9-Au53	65.60(9)	Au1-Au9-Au53	132.71(12)
Au7-Au9-Au53	165.14(12)	S17-Au9-Au8	89.3(4)
Au2-Au9-Au8	122.31(12)	Au40-Au9-Au8	92.24(10)
Au37-Au9-Au8	55.66(8)	Au1-Au9-Au8	60.22(9)
Au7-Au9-Au8	53.99(8)	Au53-Au9-Au8	120.46(11)
S17-Au9-Au44	43.1(4)	Au2-Au9-Au44	176.70(13)
Au40-Au9-Au44	121.62(11)	Au37-Au9-Au44	57.26(8)
Au1-Au9-Au44	120.61(11)	Au7-Au9-Au44	84.32(9)
Au53-Au9-Au44	81.36(9)	Au8-Au9-Au44	60.44(8)
Au63-Au10-Au11	59.32(9)	Au63-Au10-Au45	119.86(12)
Au11-Au10-Au45	121.53(12)	Au63-Au10-Au37	122.04(12)
Au11-Au10-Au37	178.63(13)	Au45-Au10-Au37	57.85(9)
Au63-Au10-Au8	179.37(13)	Au11-Au10-Au8	120.07(11)
Au45-Au10-Au8	60.21(9)	Au37-Au10-Au8	58.57(9)
Au63-Au10-Au1	117.31(11)	Au11-Au10-Au1	87.49(10)
Au45-Au10-Au1	122.82(12)	Au37-Au10-Au1	91.92(11)
Au8-Au10-Au1	62.62(9)	Au63-Au10-Au40	87.01(10)
Au11-Au10-Au40	117.06(11)	Au45-Au10-Au40	121.32(12)
Au37-Au10-Au40	63.65(9)	Au8-Au10-Au40	93.47(10)
Au1-Au10-Au40	61.35(9)	Au63-Au10-Au6	120.17(11)
Au11-Au10-Au6	60.85(9)	Au45-Au10-Au6	91.66(10)
Au37-Au10-Au6	117.79(11)	Au8-Au10-Au6	59.23(9)
Au1-Au10-Au6	58.72(8)	Au40-Au10-Au6	120.07(11)
Au63-Au10-Au3	58.23(9)	Au11-Au10-Au3	57.97(9)
Au45-Au10-Au3	178.09(13)	Au37-Au10-Au3	122.69(12)
Au8-Au10-Au3	121.70(11)	Au1-Au10-Au3	59.08(8)
Au40-Au10-Au3	59.10(9)	Au6-Au10-Au3	89.58(10)
Au63-Au10-Au62	94.95(11)	Au11-Au10-Au62	62.77(9)
Au45-Au10-Au62	59.08(9)	Au37-Au10-Au62	116.47(11)
Au8-Au10-Au62	84.56(10)	Au1-Au10-Au62	116.12(11)
Au40-Au10-Au62	177.38(12)	Au6-Au10-Au62	57.41(8)
Au3-Au10-Au62	120.60(11)	Au63-Au10-Au49	58.80(9)
Au11-Au10-Au49	118.04(11)	Au45-Au10-Au49	91.84(10)
Au37-Au10-Au49	63.33(9)	Au8-Au10-Au49	121.82(11)
Au1-Au10-Au49	118.14(11)	Au40-Au10-Au49	56.83(8)
Au6-Au10-Au49	176.29(12)	Au3-Au10-Au49	86.95(10)
Au62-Au10-Au49	125.72(11)	Au63-Au10-Au60	60.39(9)
Au11-Au10-Au60	90.34(10)	Au45-Au10-Au60	59.48(9)
Au37-Au10-Au60	90.28(10)	Au8-Au10-Au60	119.67(11)
Au1-Au10-Au60	177.49(12)	Au40-Au10-Au60	118.75(11)
Au6-Au10-Au60	121.11(11)	Au3-Au10-Au60	118.62(11)

Au62-Au10-Au60	63.81(9)	Au49-Au10-Au60	61.92(8)
S3-Au11-Au63	135.9(4)	S3-Au11-Au10	159.4(5)
Au63-Au11-Au10	60.34(9)	S3-Au11-Au3	130.5(5)
Au63-Au11-Au3	61.06(9)	Au10-Au11-Au3	65.27(9)
S3-Au11-Au6	99.4(4)	Au63-Au11-Au6	123.45(12)
Au10-Au11-Au6	63.12(9)	Au3-Au11-Au6	94.90(10)
S3-Au11-Au13	84.9(4)	Au63-Au11-Au13	123.21(12)
Au10-Au11-Au13	94.55(11)	Au3-Au11-Au13	62.18(9)
Au6-Au11-Au13	60.31(8)	S3-Au11-Au62	99.2(5)
Au63-Au11-Au62	95.65(11)	Au10-Au11-Au62	62.93(9)
Au3-Au11-Au62	128.04(12)	Au6-Au11-Au62	58.08(8)
Au13-Au11-Au62	118.09(11)	S3-Au11-Au68	70.5(5)
Au63-Au11-Au68	96.20(11)	Au10-Au11-Au68	125.80(12)
Au3-Au11-Au68	60.76(9)	Au6-Au11-Au68	116.22(11)
Au13-Au11-Au68	56.17(8)	Au62-Au11-Au68	167.93(11)
S3-Au11-Au67	45.5(4)	Au63-Au11-Au67	178.46(13)
Au10-Au11-Au67	118.13(11)	Au3-Au11-Au67	118.71(11)
Au6-Au11-Au67	55.02(8)	Au13-Au11-Au67	56.53(8)
Au62-Au11-Au67	83.35(9)	Au68-Au11-Au67	84.85(9)
Au26-Au12-Au6	121.32(12)	Au26-Au12-Au21	55.81(9)
Au6-Au12-Au21	176.31(12)	Au26-Au12-Au20	114.60(11)
Au6-Au12-Au20	92.90(10)	Au21-Au12-Au20	86.66(10)
Au26-Au12-Au13	176.36(12)	Au6-Au12-Au13	61.33(9)
Au21-Au12-Au13	121.42(12)	Au20-Au12-Au13	62.13(9)
Au26-Au12-Au25	85.84(10)	Au6-Au12-Au25	62.05(9)
Au21-Au12-Au25	114.64(11)	Au20-Au12-Au25	62.00(9)
Au13-Au12-Au25	93.67(10)	Au26-Au12-Au14	117.87(11)
Au6-Au12-Au14	120.71(11)	Au21-Au12-Au14	62.07(9)
Au20-Au12-Au14	58.98(9)	Au13-Au12-Au14	59.39(9)
Au25-Au12-Au14	120.98(11)	Au26-Au12-Au7	65.63(9)
Au6-Au12-Au7	83.96(10)	Au21-Au12-Au7	96.34(11)
Au20-Au12-Au7	176.21(12)	Au13-Au12-Au7	117.74(11)
Au25-Au12-Au7	114.49(11)	Au14-Au12-Au7	124.52(11)
Au26-Au12-Au1	124.41(11)	Au6-Au12-Au1	58.95(8)
Au21-Au12-Au1	124.30(12)	Au20-Au12-Au1	120.97(11)
Au13-Au12-Au1	58.84(8)	Au25-Au12-Au1	120.99(11)
Au14-Au12-Au1	90.37(10)	Au7-Au12-Au1	59.04(8)
Au26-Au12-Au22	62.01(9)	Au6-Au12-Au22	59.35(9)
Au21-Au12-Au22	117.82(11)	Au20-Au12-Au22	120.14(11)
Au13-Au12-Au22	120.68(11)	Au25-Au12-Au22	58.14(9)
Au14-Au12-Au22	179.06(13)	Au7-Au12-Au22	56.36(9)
Au1-Au12-Au22	90.42(10)	Au26-Au12-Au5	96.46(10)
Au6-Au12-Au5	117.47(11)	Au21-Au12-Au5	65.91(9)
Au20-Au12-Au5	115.71(11)	Au13-Au12-Au5	83.95(10)
Au25-Au12-Au5	177.36(12)	Au14-Au12-Au5	56.76(8)

Au7-Au12-Au5	67.77(9)	Au1-Au12-Au5	58.63(8)
Au22-Au12-Au5	124.13(11)	Au26-Au12-Au30	55.70(9)
Au6-Au12-Au30	120.92(11)	Au21-Au12-Au30	55.83(9)
Au20-Au12-Au30	58.93(9)	Au13-Au12-Au30	121.06(11)
Au25-Au12-Au30	58.87(9)	Au14-Au12-Au30	89.66(10)
Au7-Au12-Au30	121.05(11)	Au1-Au12-Au30	179.85(15)
Au22-Au12-Au30	89.55(10)	Au5-Au12-Au30	121.49(11)
Au1-Au13-Au69	177.55(13)	Au1-Au13-Au14	93.09(10)
Au69-Au13-Au14	87.39(10)	Au1-Au13-Au12	61.65(9)
Au69-Au13-Au12	120.58(11)	Au14-Au13-Au12	61.19(9)
Au1-Au13-Au4	61.89(9)	Au69-Au13-Au4	116.35(11)
Au14-Au13-Au4	62.75(9)	Au12-Au13-Au4	93.45(10)
Au1-Au13-Au68	121.97(11)	Au69-Au13-Au68	55.75(9)
Au14-Au13-Au68	114.75(11)	Au12-Au13-Au68	175.33(12)
Au4-Au13-Au68	86.26(10)	Au1-Au13-Au16	121.53(12)
Au69-Au13-Au16	56.78(9)	Au14-Au13-Au16	59.32(9)
Au12-Au13-Au16	120.52(11)	Au4-Au13-Au16	59.65(9)
Au68-Au13-Au16	55.47(9)	Au1-Au13-Au6	59.15(8)
Au69-Au13-Au6	122.56(12)	Au14-Au13-Au6	120.34(11)
Au12-Au13-Au6	59.15(8)	Au4-Au13-Au6	121.04(11)
Au68-Au13-Au6	124.82(12)	Au16-Au13-Au6	179.13(14)
Au1-Au13-Au11	84.20(10)	Au69-Au13-Au11	95.28(11)
Au14-Au13-Au11	177.05(13)	Au12-Au13-Au11	118.14(11)
Au4-Au13-Au11	114.77(11)	Au68-Au13-Au11	66.04(9)
Au16-Au13-Au11	121.27(11)	Au6-Au13-Au11	59.11(9)
Au1-Au13-Au3	59.52(8)	Au69-Au13-Au3	118.23(11)
Au14-Au13-Au3	121.16(11)	Au12-Au13-Au3	121.17(11)
Au4-Au13-Au3	58.41(9)	Au68-Au13-Au3	62.50(9)
Au16-Au13-Au3	90.26(10)	Au6-Au13-Au3	90.59(10)
Au11-Au13-Au3	56.39(8)	Au1-Au13-Au67	117.47(11)
Au69-Au13-Au67	64.38(9)	Au14-Au13-Au67	114.66(11)
Au12-Au13-Au67	83.78(10)	Au4-Au13-Au67	176.98(12)
Au68-Au13-Au67	96.40(11)	Au16-Au13-Au67	120.84(11)
Au6-Au13-Au67	58.44(9)	Au11-Au13-Au67	67.76(9)
Au3-Au13-Au67	124.15(11)	Au1-Au13-Au20	120.59(11)
Au69-Au13-Au20	61.66(9)	Au14-Au13-Au20	58.52(9)
Au12-Au13-Au20	58.93(8)	Au4-Au13-Au20	121.28(11)
Au68-Au13-Au20	117.37(11)	Au16-Au13-Au20	89.39(10)
Au6-Au13-Au20	89.77(10)	Au11-Au13-Au20	123.93(11)
Au3-Au13-Au20	179.62(14)	Au67-Au13-Au20	56.17(8)
Au15-Au14-Au19	120.53(12)	Au15-Au14-Au5	58.35(9)
Au19-Au14-Au5	124.72(12)	Au15-Au14-Au18	121.83(12)
Au19-Au14-Au18	57.27(9)	Au5-Au14-Au18	177.84(13)
Au15-Au14-Au20	179.61(14)	Au19-Au14-Au20	59.84(9)
Au5-Au14-Au20	121.58(12)	Au18-Au14-Au20	58.22(9)

Au15-Au14-Au16	87.04(11)	Au19-Au14-Au16	119.95(12)
Au5-Au14-Au16	115.23(11)	Au18-Au14-Au16	62.84(9)
Au20-Au14-Au16	92.67(10)	Au15-Au14-Au13	117.26(12)
Au19-Au14-Au13	122.19(12)	Au5-Au14-Au13	86.83(10)
Au18-Au14-Au13	91.29(10)	Au20-Au14-Au13	62.37(9)
Au16-Au14-Au13	61.13(9)	Au15-Au14-Au12	120.34(11)
Au19-Au14-Au12	91.68(10)	Au5-Au14-Au12	62.01(9)
Au18-Au14-Au12	117.83(12)	Au20-Au14-Au12	59.61(9)
Au16-Au14-Au12	120.55(11)	Au13-Au14-Au12	59.42(8)
Au15-Au14-Au21	95.51(11)	Au19-Au14-Au21	58.69(9)
Au5-Au14-Au21	66.30(9)	Au18-Au14-Au21	115.59(11)
Au20-Au14-Au21	84.78(10)	Au16-Au14-Au21	177.45(13)
Au13-Au14-Au21	117.41(11)	Au12-Au14-Au21	58.02(8)
Au15-Au14-Au4	58.52(9)	Au19-Au14-Au4	178.24(13)
Au5-Au14-Au4	56.36(9)	Au18-Au14-Au4	121.67(11)
Au20-Au14-Au4	121.11(11)	Au16-Au14-Au4	58.90(9)
Au13-Au14-Au4	58.74(8)	Au12-Au14-Au4	90.07(10)
Au21-Au14-Au4	122.52(11)	Au15-Au14-Au17	59.19(9)
Au19-Au14-Au17	91.15(10)	Au5-Au14-Au17	117.33(11)
Au18-Au14-Au17	62.70(9)	Au20-Au14-Au17	120.83(11)
Au16-Au14-Au17	56.57(9)	Au13-Au14-Au17	117.67(11)
Au12-Au14-Au17	176.79(12)	Au21-Au14-Au17	124.93(11)
Au4-Au14-Au17	87.09(10)	Au15-Au14-Au54	61.29(9)
Au19-Au14-Au54	59.25(9)	Au5-Au14-Au54	92.64(10)
Au18-Au14-Au54	89.21(10)	Au20-Au14-Au54	119.08(11)
Au16-Au14-Au54	117.91(11)	Au13-Au14-Au54	178.47(13)
Au12-Au14-Au54	121.54(11)	Au21-Au14-Au54	63.60(9)
Au4-Au14-Au54	119.80(11)	Au17-Au14-Au54	61.35(8)
S20-Au15-Au5	135.7(4)	S20-Au15-Au14	154.9(5)
Au5-Au15-Au14	61.72(10)	S20-Au15-Au4	136.1(5)
Au5-Au15-Au4	59.54(9)	Au14-Au15-Au4	65.24(9)
S20-Au15-Au17	94.7(4)	Au5-Au15-Au17	128.32(13)
Au14-Au15-Au17	66.87(10)	Au4-Au15-Au17	94.74(11)
S20-Au15-Au34	88.1(4)	Au5-Au15-Au34	123.17(12)
Au14-Au15-Au34	93.99(11)	Au4-Au15-Au34	63.64(9)
Au17-Au15-Au34	54.68(9)	S20-Au15-Au54	90.6(5)
Au5-Au15-Au54	97.77(11)	Au14-Au15-Au54	66.67(10)
Au4-Au15-Au54	131.90(12)	Au17-Au15-Au54	65.34(9)
Au34-Au15-Au54	119.63(11)	S20-Au15-Au43	73.5(5)
Au5-Au15-Au43	92.34(11)	Au14-Au15-Au43	129.28(12)
Au4-Au15-Au43	64.04(9)	Au17-Au15-Au43	117.08(12)
Au34-Au15-Au43	63.17(10)	Au54-Au15-Au43	164.02(12)
Au68-Au16-Au47	113.84(12)	Au68-Au16-Au69	59.08(10)
Au47-Au16-Au69	116.79(13)	Au68-Au16-Au34	120.85(12)
Au47-Au16-Au34	58.72(10)	Au69-Au16-Au34	175.39(13)

Au68-Au16-Au17	172.71(14)	Au47-Au16-Au17	58.87(10)
Au69-Au16-Au17	122.80(12)	Au34-Au16-Au17	56.61(9)
Au68-Au16-Au14	121.05(12)	Au47-Au16-Au14	125.02(12)
Au69-Au16-Au14	89.79(11)	Au34-Au16-Au14	93.85(11)
Au17-Au16-Au14	66.22(9)	Au68-Au16-Au4	89.66(10)
Au47-Au16-Au4	122.60(12)	Au69-Au16-Au4	120.13(12)
Au34-Au16-Au4	64.20(9)	Au17-Au16-Au4	94.70(11)
Au14-Au16-Au4	62.79(9)	Au68-Au16-Au13	61.55(9)
Au47-Au16-Au13	175.32(13)	Au69-Au16-Au13	60.70(9)
Au34-Au16-Au13	123.70(12)	Au17-Au16-Au13	125.74(12)
Au14-Au16-Au13	59.55(9)	Au4-Au16-Au13	59.52(9)
Au68-Au16-Au18	118.04(12)	Au47-Au16-Au18	94.36(11)
Au69-Au16-Au18	58.97(9)	Au34-Au16-Au18	120.98(12)
Au17-Au16-Au18	64.47(9)	Au14-Au16-Au18	57.76(9)
Au4-Au16-Au18	120.48(11)	Au13-Au16-Au18	87.53(10)
Au68-Au16-Au57	58.89(9)	Au47-Au16-Au57	91.10(11)
Au69-Au16-Au57	117.85(12)	Au34-Au16-Au57	62.60(9)
Au17-Au16-Au57	119.20(12)	Au14-Au16-Au57	119.07(11)
Au4-Au16-Au57	56.33(8)	Au13-Au16-Au57	86.98(10)
Au18-Au16-Au57	174.51(12)	Au68-Au16-Au72	87.14(10)
Au47-Au16-Au72	57.52(9)	Au69-Au16-Au72	59.37(9)
Au34-Au16-Au72	116.19(11)	Au17-Au16-Au72	88.31(10)
Au14-Au16-Au72	120.43(11)	Au4-Au16-Au72	176.39(12)
Au13-Au16-Au72	120.07(11)	Au18-Au16-Au72	62.68(9)
Au57-Au16-Au72	120.36(11)	S12-Au17-Au34	161.5(4)
S12-Au17-Au47	134.7(4)	Au34-Au17-Au47	59.98(10)
S12-Au17-Au16	133.6(4)	Au34-Au17-Au16	60.92(10)
Au47-Au17-Au16	58.15(10)	S12-Au17-Au15	103.9(4)
Au34-Au17-Au15	62.76(10)	Au47-Au17-Au15	121.40(12)
Au16-Au17-Au15	84.25(10)	S12-Au17-Au55	90.8(4)
Au34-Au17-Au55	73.25(11)	Au47-Au17-Au55	101.62(12)
Au16-Au17-Au55	134.11(13)	Au15-Au17-Au55	72.35(10)
S12-Au17-Au18	74.7(4)	Au34-Au17-Au18	120.43(12)
Au47-Au17-Au18	90.95(11)	Au16-Au17-Au18	59.61(9)
Au15-Au17-Au18	107.75(11)	Au55-Au17-Au18	165.22(13)
S12-Au17-Au14	90.7(4)	Au34-Au17-Au14	90.85(10)
Au47-Au17-Au14	115.30(12)	Au16-Au17-Au14	57.21(9)
Au15-Au17-Au14	53.94(8)	Au55-Au17-Au14	124.87(12)
Au18-Au17-Au14	53.86(8)	S12-Au17-Au54	45.0(4)
Au34-Au17-Au54	121.74(12)	Au47-Au17-Au54	174.75(13)
Au16-Au17-Au54	117.67(11)	Au15-Au17-Au54	59.38(9)
Au55-Au17-Au54	83.59(10)	Au18-Au17-Au54	83.97(10)
Au14-Au17-Au54	60.46(8)	S13-Au18-Au19	129.4(4)
S13-Au18-Au20	160.4(4)	Au19-Au18-Au20	62.11(10)
S13-Au18-Au14	136.5(4)	Au19-Au18-Au14	60.39(9)

Au20-Au18-Au14	61.59(9)	S13-Au18-Au69	103.2(4)
Au19-Au18-Au69	126.59(13)	Au20-Au18-Au69	64.85(10)
Au14-Au18-Au69	89.47(11)	S13-Au18-Au16	93.1(4)
Au19-Au18-Au16	119.63(12)	Au20-Au18-Au16	92.59(11)
Au14-Au18-Au16	59.40(9)	Au69-Au18-Au16	56.78(9)
S13-Au18-Au73	93.6(4)	Au19-Au18-Au73	96.81(12)
Au20-Au18-Au73	67.88(10)	Au14-Au18-Au73	129.47(12)
Au69-Au18-Au73	68.13(10)	Au16-Au18-Au73	124.57(12)
S13-Au18-Au17	73.3(4)	Au19-Au18-Au17	93.33(11)
Au20-Au18-Au17	124.95(12)	Au14-Au18-Au17	63.44(9)
Au69-Au18-Au17	112.19(11)	Au16-Au18-Au17	55.93(9)
Au73-Au18-Au17	166.65(12)	S13-Au18-Au72	44.8(4)
Au19-Au18-Au72	174.20(13)	Au20-Au18-Au72	123.44(12)
Au14-Au18-Au72	122.84(11)	Au69-Au18-Au72	59.13(9)
Au16-Au18-Au72	63.45(9)	Au73-Au18-Au72	84.51(10)
Au17-Au18-Au72	84.56(10)	S11-Au19-Au18	129.7(4)
S11-Au19-Au14	161.2(4)	Au18-Au19-Au14	62.34(10)
S11-Au19-Au20	135.2(4)	Au18-Au19-Au20	60.42(9)
Au14-Au19-Au20	61.69(9)	S11-Au19-Au21	103.4(4)
Au18-Au19-Au21	126.48(13)	Au14-Au19-Au21	64.59(10)
Au20-Au19-Au21	88.83(11)	S11-Au19-Au54	94.9(4)
Au18-Au19-Au54	96.93(12)	Au14-Au19-Au54	67.59(10)
Au20-Au19-Au54	129.27(12)	Au21-Au19-Au54	68.47(10)
S11-Au19-Au30	92.3(4)	Au18-Au19-Au30	119.55(12)
Au14-Au19-Au30	92.14(10)	Au20-Au19-Au30	59.26(9)
Au21-Au19-Au30	55.88(9)	Au54-Au19-Au30	124.02(12)
S11-Au19-Au33	72.4(4)	Au18-Au19-Au33	93.80(11)
Au14-Au19-Au33	124.58(11)	Au20-Au19-Au33	63.06(9)
Au21-Au19-Au33	110.67(11)	Au54-Au19-Au33	166.87(12)
Au30-Au19-Au33	55.28(9)	S11-Au19-Au41	44.7(4)
Au18-Au19-Au41	174.39(13)	Au14-Au19-Au41	122.92(11)
Au20-Au19-Au41	122.95(12)	Au21-Au19-Au41	59.03(9)
Au54-Au19-Au41	84.08(10)	Au30-Au19-Au41	63.70(9)
Au33-Au19-Au41	84.51(10)	Au18-Au20-Au71	121.73(12)
Au18-Au20-Au67	123.24(12)	Au71-Au20-Au67	58.19(9)
Au18-Au20-Au19	57.47(9)	Au71-Au20-Au19	122.40(12)
Au67-Au20-Au19	178.88(14)	Au18-Au20-Au14	60.19(9)
Au71-Au20-Au14	178.07(13)	Au67-Au20-Au14	120.91(12)
Au19-Au20-Au14	58.48(9)	Au18-Au20-Au30	121.02(12)
Au71-Au20-Au30	86.16(10)	Au67-Au20-Au30	115.67(12)
Au19-Au20-Au30	63.68(9)	Au14-Au20-Au30	92.90(10)
Au18-Au20-Au12	121.59(12)	Au71-Au20-Au12	116.67(12)
Au67-Au20-Au12	86.92(10)	Au19-Au20-Au12	91.96(11)
Au14-Au20-Au12	61.40(9)	Au30-Au20-Au12	62.08(9)
Au18-Au20-Au13	90.49(10)	Au71-Au20-Au13	120.03(12)

Au67-Au20-Au13	61.84(9)	Au19-Au20-Au13	117.57(12)
Au14-Au20-Au13	59.11(9)	Au30-Au20-Au13	121.02(11)
Au12-Au20-Au13	58.93(8)	Au18-Au20-Au25	179.13(14)
Au71-Au20-Au25	57.43(9)	Au67-Au20-Au25	56.71(9)
Au19-Au20-Au25	122.60(12)	Au14-Au20-Au25	120.65(11)
Au30-Au20-Au25	58.99(9)	Au12-Au20-Au25	59.25(9)
Au13-Au20-Au25	90.20(10)	Au18-Au20-Au69	58.53(9)
Au71-Au20-Au69	95.52(11)	Au67-Au20-Au69	64.90(9)
Au19-Au20-Au69	115.72(12)	Au14-Au20-Au69	85.40(10)
Au30-Au20-Au69	178.22(13)	Au12-Au20-Au69	116.49(11)
Au13-Au20-Au69	57.57(9)	Au25-Au20-Au69	121.49(12)
Au18-Au20-Au33	92.23(10)	Au71-Au20-Au33	59.25(9)
Au67-Au20-Au33	117.34(11)	Au19-Au20-Au33	63.19(9)
Au14-Au20-Au33	121.51(11)	Au30-Au20-Au33	56.44(9)
Au12-Au20-Au33	118.50(11)	Au13-Au20-Au33	177.05(12)
Au25-Au20-Au33	87.09(10)	Au69-Au20-Au33	125.01(11)
Au18-Au20-Au73	59.22(9)	Au71-Au20-Au73	62.52(9)
Au67-Au20-Au73	91.95(10)	Au19-Au20-Au73	89.17(10)
Au14-Au20-Au73	119.41(11)	Au30-Au20-Au73	118.37(11)
Au12-Au20-Au73	178.86(13)	Au13-Au20-Au73	120.60(11)
Au25-Au20-Au73	119.94(11)	Au69-Au20-Au73	63.08(9)
Au33-Au20-Au73	61.94(9)	S10-Au21-Au26	131.6(4)
S10-Au21-Au30	161.5(4)	Au26-Au21-Au30	60.39(10)
S10-Au21-Au19	101.9(4)	Au26-Au21-Au19	125.49(13)
Au30-Au21-Au19	65.22(10)	S10-Au21-Au12	132.3(4)
Au26-Au21-Au12	61.97(9)	Au30-Au21-Au12	63.85(9)
Au19-Au21-Au12	91.66(11)	S10-Au21-Au14	90.2(4)
Au26-Au21-Au14	121.88(12)	Au30-Au21-Au14	93.00(11)
Au19-Au21-Au14	56.73(9)	Au12-Au21-Au14	59.91(9)
S10-Au21-Au41	93.1(4)	Au26-Au21-Au41	96.03(12)
Au30-Au21-Au41	70.07(10)	Au19-Au21-Au41	67.86(10)
Au12-Au21-Au41	133.91(12)	Au14-Au21-Au41	123.88(11)
S10-Au21-Au5	74.2(4)	Au26-Au21-Au5	95.31(11)
Au30-Au21-Au5	121.84(11)	Au19-Au21-Au5	110.41(11)
Au12-Au21-Au5	58.24(8)	Au14-Au21-Au5	53.88(8)
Au41-Au21-Au5	166.78(12)	S10-Au21-Au54	44.7(4)
Au26-Au21-Au54	176.06(13)	Au30-Au21-Au54	122.75(12)
Au19-Au21-Au54	57.85(9)	Au12-Au21-Au54	121.12(11)
Au14-Au21-Au54	61.29(8)	Au41-Au21-Au54	83.33(10)
Au5-Au21-Au54	84.90(9)	Au23-Au22-Au29	118.53(12)
Au23-Au22-Au7	59.00(9)	Au29-Au22-Au7	177.22(13)
Au23-Au22-Au27	121.21(12)	Au29-Au22-Au27	57.99(9)
Au7-Au22-Au27	124.06(12)	Au23-Au22-Au25	178.74(13)
Au29-Au22-Au25	60.93(9)	Au7-Au22-Au25	121.50(12)
Au27-Au22-Au25	59.64(9)	Au23-Au22-Au6	116.61(12)

Au29-Au22-Au6	93.69(11)	Au7-Au22-Au6	86.59(10)
Au27-Au22-Au6	122.16(12)	Au25-Au22-Au6	62.52(9)
Au23-Au22-Au24	86.11(10)	Au29-Au22-Au24	62.22(9)
Au7-Au22-Au24	115.66(11)	Au27-Au22-Au24	120.20(12)
Au25-Au22-Au24	92.65(10)	Au6-Au22-Au24	61.58(9)
Au23-Au22-Au12	120.61(12)	Au29-Au22-Au12	120.84(12)
Au7-Au22-Au12	61.61(9)	Au27-Au22-Au12	91.61(10)
Au25-Au22-Au12	59.91(9)	Au6-Au22-Au12	58.88(8)
Au24-Au22-Au12	120.46(11)	Au23-Au22-Au8	57.85(9)
Au29-Au22-Au8	121.09(12)	Au7-Au22-Au8	56.81(9)
Au27-Au22-Au8	178.45(13)	Au25-Au22-Au8	121.28(11)
Au6-Au22-Au8	58.76(9)	Au24-Au22-Au8	58.86(8)
Au12-Au22-Au8	89.94(10)	Au23-Au22-Au26	96.86(11)
Au29-Au22-Au26	116.36(12)	Au7-Au22-Au26	65.86(9)
Au27-Au22-Au26	58.49(9)	Au25-Au22-Au26	84.38(10)
Au6-Au22-Au26	116.44(11)	Au24-Au22-Au26	177.01(13)
Au12-Au22-Au26	57.60(8)	Au8-Au22-Au26	122.50(11)
Au23-Au22-Au28	61.41(9)	Au29-Au22-Au28	87.87(10)
Au7-Au22-Au28	91.79(10)	Au27-Au22-Au28	59.83(9)
Au25-Au22-Au28	119.46(11)	Au6-Au22-Au28	177.96(12)
Au24-Au22-Au28	118.18(11)	Au12-Au22-Au28	121.32(11)
Au8-Au22-Au28	119.26(11)	Au26-Au22-Au28	63.88(9)
Au23-Au22-Au38	59.39(9)	Au29-Au22-Au38	59.23(9)
Au7-Au22-Au38	118.22(11)	Au27-Au22-Au38	91.67(10)
Au25-Au22-Au38	119.99(11)	Au6-Au22-Au38	117.15(11)
Au24-Au22-Au38	55.59(8)	Au12-Au22-Au38	175.88(12)
Au8-Au22-Au38	86.78(9)	Au26-Au22-Au38	126.40(11)
Au28-Au22-Au38	62.60(8)	S29-Au23-Au7	132.2(5)
S29-Au23-Au22	152.0(8)	Au7-Au23-Au22	61.34(10)
S29-Au23-Au8	141.4(8)	Au7-Au23-Au8	59.92(9)
Au22-Au23-Au8	65.19(10)	S29-Au23-Au39	92.3(6)
Au7-Au23-Au39	125.22(12)	Au22-Au23-Au39	95.42(11)
Au8-Au23-Au39	65.31(9)	S29-Au23-Au38	95.7(6)
Au7-Au23-Au38	129.10(13)	Au22-Au23-Au38	67.98(10)
Au8-Au23-Au38	95.20(11)	Au39-Au23-Au38	54.01(9)
S29-Au23-Au28	86.9(8)	Au7-Au23-Au28	95.81(11)
Au22-Au23-Au28	66.06(9)	Au8-Au23-Au28	131.25(12)
Au39-Au23-Au28	120.36(12)	Au38-Au23-Au28	66.74(9)
S29-Au23-Au44	76.0(8)	Au7-Au23-Au44	93.38(11)
Au22-Au23-Au44	131.29(12)	Au8-Au23-Au44	66.10(9)
Au39-Au23-Au44	64.45(9)	Au38-Au23-Au44	117.55(11)
Au28-Au23-Au44	162.62(12)	Au51-Au24-Au62	114.88(12)
Au51-Au24-Au64	117.22(12)	Au62-Au24-Au64	58.68(9)
Au51-Au24-Au39	59.11(9)	Au62-Au24-Au39	121.89(12)
Au64-Au24-Au39	176.32(13)	Au51-Au24-Au38	58.65(9)

Au62-Au24-Au38	173.52(13)	Au64-Au24-Au38	122.80(12)
Au39-Au24-Au38	56.16(9)	Au51-Au24-Au8	123.90(12)
Au62-Au24-Au8	89.13(10)	Au64-Au24-Au8	118.53(12)
Au39-Au24-Au8	65.10(9)	Au38-Au24-Au8	95.04(11)
Au51-Au24-Au22	125.88(12)	Au62-Au24-Au22	119.11(12)
Au64-Au24-Au22	88.61(11)	Au39-Au24-Au22	93.95(11)
Au38-Au24-Au22	67.33(9)	Au8-Au24-Au22	61.59(9)
Au51-Au24-Au29	93.42(11)	Au62-Au24-Au29	120.54(12)
Au64-Au24-Au29	61.90(9)	Au39-Au24-Au29	117.55(12)
Au38-Au24-Au29	61.62(9)	Au8-Au24-Au29	117.72(11)
Au22-Au24-Au29	56.13(9)	Au51-Au24-Au6	175.03(13)
Au62-Au24-Au6	60.30(9)	Au64-Au24-Au6	59.80(9)
Au39-Au24-Au6	123.84(12)	Au38-Au24-Au6	126.16(12)
Au8-Au24-Au6	58.76(8)	Au22-Au24-Au6	58.85(9)
Au29-Au24-Au6	88.45(10)	Au51-Au24-Au45	91.63(10)
Au62-Au24-Au45	59.31(9)	Au64-Au24-Au45	117.86(11)
Au39-Au24-Au45	63.09(9)	Au38-Au24-Au45	119.25(12)
Au8-Au24-Au45	56.91(8)	Au22-Au24-Au45	118.45(11)
Au29-Au24-Au45	174.26(12)	Au6-Au24-Au45	86.73(9)
Au51-Au24-Au59	58.39(9)	Au62-Au24-Au59	88.09(10)
Au64-Au24-Au59	59.00(9)	Au39-Au24-Au59	117.35(11)
Au38-Au24-Au59	87.81(10)	Au8-Au24-Au59	177.04(12)
Au22-Au24-Au59	119.09(11)	Au29-Au24-Au59	63.05(9)
Au6-Au24-Au59	118.80(11)	Au45-Au24-Au59	122.20(11)
Au67-Au25-Au71	58.81(9)	Au67-Au25-Au27	178.71(14)
Au71-Au25-Au27	122.12(12)	Au67-Au25-Au29	121.95(12)
Au71-Au25-Au29	124.03(13)	Au27-Au25-Au29	56.87(9)
Au67-Au25-Au22	119.79(12)	Au71-Au25-Au22	178.01(14)
Au27-Au25-Au22	59.31(9)	Au29-Au25-Au22	57.78(9)
Au67-Au25-Au30	117.19(12)	Au71-Au25-Au30	86.10(11)
Au27-Au25-Au30	64.00(9)	Au29-Au25-Au30	120.86(12)
Au22-Au25-Au30	93.54(11)	Au67-Au25-Au12	87.60(10)
Au71-Au25-Au12	116.23(12)	Au27-Au25-Au12	92.64(11)
Au29-Au25-Au12	119.73(12)	Au22-Au25-Au12	61.95(9)
Au30-Au25-Au12	61.85(9)	Au67-Au25-Au6	60.37(9)
Au71-Au25-Au6	119.13(12)	Au27-Au25-Au6	118.74(12)
Au29-Au25-Au6	89.99(10)	Au22-Au25-Au6	59.43(9)
Au30-Au25-Au6	120.39(11)	Au12-Au25-Au6	58.55(8)
Au67-Au25-Au64	61.64(9)	Au71-Au25-Au64	94.67(11)
Au27-Au25-Au64	117.15(12)	Au29-Au25-Au64	60.36(9)
Au22-Au25-Au64	85.61(10)	Au30-Au25-Au64	177.74(13)
Au12-Au25-Au64	115.95(11)	Au6-Au25-Au64	57.40(8)
Au67-Au25-Au20	58.39(9)	Au71-Au25-Au20	57.48(9)
Au27-Au25-Au20	122.78(12)	Au29-Au25-Au20	178.48(14)
Au22-Au25-Au20	120.70(12)	Au30-Au25-Au20	58.84(9)

Au12-Au25-Au20	58.75(9)	Au6-Au25-Au20	88.99(10)
Au64-Au25-Au20	119.88(12)	Au67-Au25-Au31	118.41(12)
Au71-Au25-Au31	59.64(9)	Au27-Au25-Au31	62.56(9)
Au29-Au25-Au31	93.82(11)	Au22-Au25-Au31	121.69(12)
Au30-Au25-Au31	56.18(9)	Au12-Au25-Au31	118.01(11)
Au6-Au25-Au31	175.91(12)	Au64-Au25-Au31	126.00(12)
Au20-Au25-Au31	87.17(10)	Au67-Au25-Au66	90.77(11)
Au71-Au25-Au66	61.10(10)	Au27-Au25-Au66	89.04(11)
Au29-Au25-Au66	62.94(10)	Au22-Au25-Au66	120.72(12)
Au30-Au25-Au66	117.23(11)	Au12-Au25-Au66	177.33(13)
Au6-Au25-Au66	122.27(11)	Au64-Au25-Au66	64.95(9)
Au20-Au25-Au66	118.58(11)	Au31-Au25-Au66	61.06(9)
S8-Au26-Au21	130.3(4)	S8-Au26-Au30	161.0(4)
Au21-Au26-Au30	60.71(10)	S8-Au26-Au27	102.1(4)
Au21-Au26-Au27	126.20(13)	Au30-Au26-Au27	65.55(10)
S8-Au26-Au12	133.3(4)	Au21-Au26-Au12	62.22(10)
Au30-Au26-Au12	64.04(9)	Au27-Au26-Au12	92.54(11)
S8-Au26-Au46	92.6(4)	Au21-Au26-Au46	94.71(12)
Au30-Au26-Au46	69.69(10)	Au27-Au26-Au46	68.46(10)
Au12-Au26-Au46	133.73(12)	S8-Au26-Au22	91.2(4)
Au21-Au26-Au22	122.61(12)	Au30-Au26-Au22	93.37(11)
Au27-Au26-Au22	57.11(9)	Au12-Au26-Au22	60.39(9)
Au46-Au26-Au22	124.98(12)	S8-Au26-Au7	75.1(4)
Au21-Au26-Au7	95.58(11)	Au30-Au26-Au7	122.07(11)
Au27-Au26-Au7	110.67(11)	Au12-Au26-Au7	58.31(8)
Au46-Au26-Au7	167.34(12)	Au22-Au26-Au7	53.78(8)
S8-Au26-Au28	45.2(4)	Au21-Au26-Au28	175.44(13)
Au30-Au26-Au28	123.47(12)	Au27-Au26-Au28	58.10(9)
Au12-Au26-Au28	120.59(11)	Au46-Au26-Au28	85.57(10)
Au22-Au26-Au28	60.35(8)	Au7-Au26-Au28	83.60(9)
S9-Au27-Au29	133.3(5)	S9-Au27-Au22	157.8(6)
Au29-Au27-Au22	60.04(9)	S9-Au27-Au25	138.3(6)
Au29-Au27-Au25	62.12(10)	Au22-Au27-Au25	61.05(9)
S9-Au27-Au26	100.8(5)	Au29-Au27-Au26	124.28(13)
Au22-Au27-Au26	64.39(10)	Au25-Au27-Au26	88.23(11)
S9-Au27-Au28	93.5(6)	Au29-Au27-Au28	92.59(11)
Au22-Au27-Au28	66.09(10)	Au25-Au27-Au28	127.14(12)
Au26-Au27-Au28	68.05(10)	S9-Au27-Au30	92.6(6)
Au29-Au27-Au30	121.42(12)	Au22-Au27-Au30	91.77(10)
Au25-Au27-Au30	59.30(9)	Au26-Au27-Au30	55.51(9)
Au28-Au27-Au30	123.38(12)	S9-Au27-Au31	75.1(6)
Au29-Au27-Au31	97.62(12)	Au22-Au27-Au31	124.58(12)
Au25-Au27-Au31	63.71(9)	Au26-Au27-Au31	110.35(12)
Au28-Au27-Au31	168.12(12)	Au30-Au27-Au31	55.34(9)
S9-Au27-Au46	43.6(5)	Au29-Au27-Au46	175.95(13)

Au22-Au27-Au46	121.89(12)	Au25-Au27-Au46	121.86(12)
Au26-Au27-Au46	58.05(9)	Au28-Au27-Au46	85.33(10)
Au30-Au27-Au46	62.56(9)	Au31-Au27-Au46	84.10(10)
S16-Au28-S8	176.0(6)	S16-Au28-Au27	83.5(4)
S8-Au28-Au27	100.3(4)	S16-Au28-Au23	99.8(4)
S8-Au28-Au23	80.5(4)	Au27-Au28-Au23	106.59(11)
S16-Au28-Au22	94.1(4)	S8-Au28-Au22	89.3(4)
Au27-Au28-Au22	54.08(8)	Au23-Au28-Au22	52.53(8)
S16-Au28-Au26	136.4(4)	S8-Au28-Au26	47.6(4)
Au27-Au28-Au26	53.85(8)	Au23-Au28-Au26	86.13(10)
Au22-Au28-Au26	55.77(8)	S16-Au28-Au38	45.7(4)
S8-Au28-Au38	135.5(4)	Au27-Au28-Au38	86.39(10)
Au23-Au28-Au38	55.71(8)	Au22-Au28-Au38	59.31(8)
Au26-Au28-Au38	115.02(10)	S15-Au29-Au27	129.7(4)
S15-Au29-Au22	161.9(4)	Au27-Au29-Au22	61.97(10)
S15-Au29-Au25	134.7(4)	Au27-Au29-Au25	61.01(10)
Au22-Au29-Au25	61.29(9)	S15-Au29-Au64	93.3(4)
Au27-Au29-Au64	123.13(12)	Au22-Au29-Au64	88.48(10)
Au25-Au29-Au64	62.21(9)	S15-Au29-Au24	104.8(4)
Au27-Au29-Au24	123.60(12)	Au22-Au29-Au24	61.65(9)
Au25-Au29-Au24	92.53(10)	Au64-Au29-Au24	56.18(9)
S15-Au29-Au38	94.9(4)	Au27-Au29-Au38	99.30(12)
Au22-Au29-Au38	68.02(10)	Au25-Au29-Au38	129.10(12)
Au64-Au29-Au38	113.67(11)	Au24-Au29-Au38	58.10(9)
S15-Au29-Au66	72.0(4)	Au27-Au29-Au66	91.47(11)
Au22-Au29-Au66	124.63(12)	Au25-Au29-Au66	63.34(10)
Au64-Au29-Au66	65.67(10)	Au24-Au29-Au66	121.59(12)
Au38-Au29-Au66	166.63(13)	S15-Au29-Au59	44.6(3)
Au27-Au29-Au59	172.86(13)	Au22-Au29-Au59	124.78(12)
Au25-Au29-Au59	118.81(11)	Au64-Au29-Au59	57.65(8)
Au24-Au29-Au59	63.24(9)	Au38-Au29-Au59	86.18(10)
Au66-Au29-Au59	82.50(10)	Au32-Au30-Au26	116.10(13)
Au32-Au30-Au21	115.73(12)	Au26-Au30-Au21	58.90(9)
Au32-Au30-Au31	58.59(10)	Au26-Au30-Au31	121.91(13)
Au21-Au30-Au31	174.28(13)	Au32-Au30-Au33	58.84(10)
Au26-Au30-Au33	174.93(13)	Au21-Au30-Au33	122.60(13)
Au31-Au30-Au33	56.04(10)	Au32-Au30-Au20	124.47(12)
Au26-Au30-Au20	119.22(12)	Au21-Au30-Au20	89.32(11)
Au31-Au30-Au20	94.70(11)	Au33-Au30-Au20	65.85(10)
Au32-Au30-Au25	124.49(13)	Au26-Au30-Au25	88.71(11)
Au21-Au30-Au25	119.53(12)	Au31-Au30-Au25	66.07(10)
Au33-Au30-Au25	94.21(11)	Au20-Au30-Au25	62.17(9)
Au32-Au30-Au12	175.42(13)	Au26-Au30-Au12	60.26(9)
Au21-Au30-Au12	60.32(9)	Au31-Au30-Au12	125.32(12)
Au33-Au30-Au12	124.81(12)	Au20-Au30-Au12	58.99(9)

Au25-Au30-Au12	59.28(9)	Au32-Au30-Au19	93.20(11)
Au26-Au30-Au19	117.71(12)	Au21-Au30-Au19	58.91(9)
Au31-Au30-Au19	120.30(12)	Au33-Au30-Au19	64.30(10)
Au20-Au30-Au19	57.06(9)	Au25-Au30-Au19	119.15(11)
Au12-Au30-Au19	86.53(10)	Au32-Au30-Au27	93.21(11)
Au26-Au30-Au27	58.94(9)	Au21-Au30-Au27	117.78(12)
Au31-Au30-Au27	63.60(10)	Au33-Au30-Au27	119.55(12)
Au20-Au30-Au27	118.81(11)	Au25-Au30-Au27	56.70(9)
Au12-Au30-Au27	87.07(10)	Au19-Au30-Au27	173.58(12)
Au32-Au30-Au46	57.18(9)	Au26-Au30-Au46	58.97(9)
Au21-Au30-Au46	87.69(11)	Au31-Au30-Au46	88.29(11)
Au33-Au30-Au46	115.97(11)	Au20-Au30-Au46	177.01(12)
Au25-Au30-Au46	119.43(11)	Au12-Au30-Au46	119.22(11)
Au19-Au30-Au46	121.15(11)	Au27-Au30-Au46	62.73(9)
Au32-Au30-Au41	56.64(9)	Au26-Au30-Au41	88.94(10)
Au21-Au30-Au41	59.18(9)	Au31-Au30-Au41	115.18(11)
Au33-Au30-Au41	88.13(10)	Au20-Au30-Au41	119.26(11)
Au25-Au30-Au41	177.64(12)	Au12-Au30-Au41	119.49(11)
Au19-Au30-Au41	62.21(9)	Au27-Au30-Au41	121.75(11)
Au46-Au30-Au41	59.05(8)	S42-Au31-Au33	156.2(6)
S42-Au31-Au32	135.5(5)	Au33-Au31-Au32	60.91(11)
S42-Au31-Au30	138.2(6)	Au33-Au31-Au30	62.08(10)
Au32-Au31-Au30	58.37(10)	S42-Au31-Au71	102.5(5)
Au33-Au31-Au71	62.54(10)	Au32-Au31-Au71	121.73(13)
Au30-Au31-Au71	83.80(11)	S42-Au31-Au27	78.0(6)
Au33-Au31-Au27	123.04(13)	Au32-Au31-Au27	91.53(12)
Au30-Au31-Au27	61.06(10)	Au71-Au31-Au27	107.89(11)
S42-Au31-Au25	92.3(6)	Au33-Au31-Au25	92.62(12)
Au32-Au31-Au25	115.96(12)	Au30-Au31-Au25	57.75(9)
Au71-Au31-Au25	54.22(9)	Au27-Au31-Au25	53.73(9)
S42-Au31-Au66	44.7(5)	Au33-Au31-Au66	120.85(13)
Au32-Au31-Au66	174.90(14)	Au30-Au31-Au66	117.58(12)
Au71-Au31-Au66	58.97(9)	Au27-Au31-Au66	83.57(10)
Au25-Au31-Au66	59.84(9)	S39-Au32-Au30	165.7(5)
S39-Au32-Au31	126.6(6)	Au30-Au32-Au31	63.04(10)
S39-Au32-Au33	130.1(5)	Au30-Au32-Au33	62.99(10)
Au31-Au32-Au33	58.66(10)	S39-Au32-Au56	50.7(5)
Au30-Au32-Au56	143.56(14)	Au31-Au32-Au56	88.73(13)
Au33-Au32-Au56	83.14(12)	S39-Au32-Au46	95.6(5)
Au30-Au32-Au46	71.34(11)	Au31-Au32-Au46	98.76(13)
Au33-Au32-Au46	134.26(13)	Au56-Au32-Au46	139.61(15)
S39-Au32-Au41	97.6(6)	Au30-Au32-Au41	72.44(11)
Au31-Au32-Au41	135.40(13)	Au33-Au32-Au41	99.34(12)
Au56-Au32-Au41	129.66(15)	Au46-Au32-Au41	67.99(10)
S14-Au33-Au31	160.2(4)	S14-Au33-Au32	132.5(4)

Au31-Au33-Au32	60.43(11)	S14-Au33-Au30	135.9(4)
Au31-Au33-Au30	61.89(10)	Au32-Au33-Au30	58.17(10)
S14-Au33-Au71	105.4(4)	Au31-Au33-Au71	63.38(10)
Au32-Au33-Au71	122.07(13)	Au30-Au33-Au71	84.11(10)
S14-Au33-Au20	92.5(4)	Au31-Au33-Au20	93.11(11)
Au32-Au33-Au20	115.70(12)	Au30-Au33-Au20	57.71(9)
Au71-Au33-Au20	54.54(9)	S14-Au33-Au19	75.9(4)
Au31-Au33-Au19	122.26(13)	Au32-Au33-Au19	90.47(11)
Au30-Au33-Au19	60.42(9)	Au71-Au33-Au19	108.26(11)
Au20-Au33-Au19	53.75(8)	S14-Au33-Au74	89.8(4)
Au31-Au33-Au74	71.37(11)	Au32-Au33-Au74	102.88(12)
Au30-Au33-Au74	133.02(13)	Au71-Au33-Au74	70.66(10)
Au20-Au33-Au74	123.65(11)	Au19-Au33-Au74	164.91(13)
S14-Au33-Au73	45.6(4)	Au31-Au33-Au73	123.32(13)
Au32-Au33-Au73	173.94(13)	Au30-Au33-Au73	118.34(12)
Au71-Au33-Au73	60.57(9)	Au20-Au33-Au73	60.63(9)
Au19-Au33-Au73	83.48(10)	Au74-Au33-Au73	83.10(10)
S34-Au34-Au17	151.0(4)	S34-Au34-Au47	130.7(4)
Au17-Au34-Au47	61.02(10)	S34-Au34-Au16	145.7(4)
Au17-Au34-Au16	62.47(10)	Au47-Au34-Au16	58.96(10)
S34-Au34-Au15	105.0(4)	Au17-Au34-Au15	62.56(10)
Au47-Au34-Au15	122.22(12)	Au16-Au34-Au15	84.89(11)
S34-Au34-Au4	98.6(4)	Au17-Au34-Au4	95.10(11)
Au47-Au34-Au4	117.85(12)	Au16-Au34-Au4	59.19(9)
Au15-Au34-Au4	56.47(9)	S34-Au34-Au57	83.7(4)
Au17-Au34-Au57	124.80(12)	Au47-Au34-Au57	91.08(11)
Au16-Au34-Au57	62.33(9)	Au15-Au34-Au57	111.36(11)
Au4-Au34-Au57	54.91(8)	S34-Au34-Au43	47.2(4)
Au17-Au34-Au43	122.75(12)	Au47-Au34-Au43	175.78(13)
Au16-Au34-Au43	120.23(12)	Au15-Au34-Au43	61.06(9)
Au4-Au34-Au43	61.06(9)	Au57-Au34-Au43	85.06(10)
S33-Au35-Au57	129.4(4)	S33-Au35-Au3	160.1(4)
Au57-Au35-Au3	61.26(10)	S33-Au35-Au4	137.5(5)
Au57-Au35-Au4	60.77(9)	Au3-Au35-Au4	61.17(9)
S33-Au35-Au2	95.8(4)	Au57-Au35-Au2	122.75(12)
Au3-Au35-Au2	88.70(10)	Au4-Au35-Au2	62.09(9)
S33-Au35-Au42	94.3(4)	Au57-Au35-Au42	98.02(11)
Au3-Au35-Au42	66.20(9)	Au4-Au35-Au42	127.14(12)
Au2-Au35-Au42	113.43(11)	S33-Au35-Au40	105.8(4)
Au57-Au35-Au40	122.27(12)	Au3-Au35-Au40	61.01(9)
Au4-Au35-Au40	91.46(10)	Au2-Au35-Au40	56.05(9)
Au42-Au35-Au40	57.88(9)	S33-Au35-Au43	74.6(4)
Au57-Au35-Au43	91.86(11)	Au3-Au35-Au43	124.50(12)
Au4-Au35-Au43	63.33(9)	Au2-Au35-Au43	65.24(9)
Au42-Au35-Au43	168.39(12)	Au40-Au35-Au43	121.12(11)

S33-Au35-Au75	45.0(4)	Au57-Au35-Au75	172.93(13)
Au3-Au35-Au75	125.54(11)	Au4-Au35-Au75	119.45(11)
Au2-Au35-Au75	58.43(9)	Au42-Au35-Au75	87.25(10)
Au40-Au35-Au75	64.58(9)	Au43-Au35-Au75	82.43(10)
S18-Au36-Au40	163.9(5)	S18-Au36-Au49	127.6(4)
Au40-Au36-Au49	63.75(10)	S18-Au36-Au42	131.2(5)
Au40-Au36-Au42	63.22(10)	Au49-Au36-Au42	58.58(10)
S18-Au36-Au50	49.7(4)	Au40-Au36-Au50	145.88(14)
Au49-Au36-Au50	84.95(12)	Au42-Au36-Au50	89.66(12)
S18-Au36-Au53	92.8(5)	Au40-Au36-Au53	72.98(10)
Au49-Au36-Au53	98.50(11)	Au42-Au36-Au53	135.95(13)
Au50-Au36-Au53	128.10(13)	S18-Au36-Au75	96.9(4)
Au40-Au36-Au75	71.83(10)	Au49-Au36-Au75	135.29(12)
Au42-Au36-Au75	97.41(12)	Au50-Au36-Au75	135.79(13)
Au53-Au36-Au75	72.17(10)	S22-Au37-Au45	127.3(4)
S22-Au37-Au8	161.2(4)	Au45-Au37-Au8	62.24(9)
S22-Au37-Au10	136.3(4)	Au45-Au37-Au10	60.62(9)
Au8-Au37-Au10	61.66(9)	S22-Au37-Au9	104.6(4)
Au45-Au37-Au9	127.39(12)	Au8-Au37-Au9	65.58(9)
Au10-Au37-Au9	89.30(10)	S22-Au37-Au44	93.9(4)
Au45-Au37-Au44	96.33(11)	Au8-Au37-Au44	67.88(10)
Au10-Au37-Au44	129.54(12)	Au9-Au37-Au44	69.41(10)
S22-Au37-Au40	94.1(4)	Au45-Au37-Au40	120.21(12)
Au8-Au37-Au40	92.94(10)	Au10-Au37-Au40	59.76(9)
Au9-Au37-Au40	55.88(9)	Au44-Au37-Au40	124.97(12)
S22-Au37-Au49	73.0(4)	Au45-Au37-Au49	93.41(11)
Au8-Au37-Au49	124.98(12)	Au10-Au37-Au49	63.41(9)
Au9-Au37-Au49	111.25(11)	Au44-Au37-Au49	166.73(12)
Au40-Au37-Au49	55.87(8)	S22-Au37-Au53	45.1(4)
Au45-Au37-Au53	172.35(12)	Au8-Au37-Au53	124.67(11)
Au10-Au37-Au53	124.42(11)	Au9-Au37-Au53	59.98(9)
Au44-Au37-Au53	84.35(10)	Au40-Au37-Au53	64.69(9)
Au49-Au37-Au53	84.80(10)	S16-Au38-Au39	154.6(4)
S16-Au38-Au51	138.8(4)	Au39-Au38-Au51	60.68(10)
S16-Au38-Au24	138.5(4)	Au39-Au38-Au24	61.35(10)
Au51-Au38-Au24	57.81(9)	S16-Au38-Au29	79.3(4)
Au39-Au38-Au29	121.37(12)	Au51-Au38-Au29	92.07(11)
Au24-Au38-Au29	60.28(9)	S16-Au38-Au23	100.0(4)
Au39-Au38-Au23	62.20(9)	Au51-Au38-Au23	120.99(12)
Au24-Au38-Au23	82.92(10)	Au29-Au38-Au23	105.30(11)
S16-Au38-Au22	91.8(4)	Au39-Au38-Au22	90.68(10)
Au51-Au38-Au22	114.82(12)	Au24-Au38-Au22	57.08(9)
Au29-Au38-Au22	52.75(8)	Au23-Au38-Au22	52.62(8)
S16-Au38-Au76	89.5(4)	Au39-Au38-Au76	67.74(10)
Au51-Au38-Au76	101.29(12)	Au24-Au38-Au76	128.84(12)

Au29-Au38-Au76	166.55(13)	Au23-Au38-Au76	69.11(10)
Au22-Au38-Au76	120.99(11)	S16-Au38-Au28	44.1(4)
Au39-Au38-Au28	119.37(12)	Au51-Au38-Au28	172.57(13)
Au24-Au38-Au28	115.17(11)	Au29-Au38-Au28	81.69(10)
Au23-Au38-Au28	57.56(9)	Au22-Au38-Au28	58.09(8)
Au76-Au38-Au28	85.08(10)	S24-Au39-Au38	154.8(4)
S24-Au39-Au51	133.7(4)	Au38-Au39-Au51	60.56(10)
S24-Au39-Au24	140.9(4)	Au38-Au39-Au24	62.49(10)
Au51-Au39-Au24	58.14(9)	S24-Au39-Au23	103.4(4)
Au38-Au39-Au23	63.79(10)	Au51-Au39-Au23	122.40(12)
Au24-Au39-Au23	84.20(10)	S24-Au39-Au45	79.9(4)
Au38-Au39-Au45	124.11(12)	Au51-Au39-Au45	90.22(11)
Au24-Au39-Au45	61.62(9)	Au23-Au39-Au45	109.49(11)
S24-Au39-Au8	94.4(4)	Au38-Au39-Au8	94.65(11)
Au51-Au39-Au8	116.27(12)	Au24-Au39-Au8	58.39(9)
Au23-Au39-Au8	54.86(8)	Au45-Au39-Au8	54.67(8)
S24-Au39-Au44	44.3(4)	Au38-Au39-Au44	123.39(12)
Au51-Au39-Au44	174.93(13)	Au24-Au39-Au44	119.85(11)
Au23-Au39-Au44	60.64(9)	Au45-Au39-Au44	84.82(10)
Au8-Au39-Au44	61.46(8)	S24-Au39-Au76	90.8(4)
Au38-Au39-Au76	64.57(10)	Au51-Au39-Au76	99.24(11)
Au24-Au39-Au76	126.82(12)	Au23-Au39-Au76	68.42(9)
Au45-Au39-Au76	169.86(12)	Au8-Au39-Au76	122.71(11)
Au44-Au39-Au76	85.64(10)	Au36-Au40-Au9	115.19(12)
Au36-Au40-Au2	116.18(12)	Au9-Au40-Au2	58.46(9)
Au36-Au40-Au42	59.15(10)	Au9-Au40-Au42	174.27(13)
Au2-Au40-Au42	122.16(12)	Au36-Au40-Au49	58.85(9)
Au9-Au40-Au49	122.94(12)	Au2-Au40-Au49	175.02(13)
Au42-Au40-Au49	55.89(9)	Au36-Au40-Au10	124.11(12)
Au9-Au40-Au10	89.53(10)	Au2-Au40-Au10	119.40(11)
Au42-Au40-Au10	94.62(11)	Au49-Au40-Au10	65.57(9)
Au36-Au40-Au3	124.76(12)	Au9-Au40-Au3	119.84(11)
Au2-Au40-Au3	89.39(10)	Au42-Au40-Au3	65.74(9)
Au49-Au40-Au3	93.54(11)	Au10-Au40-Au3	62.09(9)
Au36-Au40-Au1	175.38(13)	Au9-Au40-Au1	60.58(9)
Au2-Au40-Au1	60.45(9)	Au42-Au40-Au1	125.04(12)
Au49-Au40-Au1	124.53(12)	Au10-Au40-Au1	59.00(8)
Au3-Au40-Au1	59.32(9)	Au36-Au40-Au35	92.14(11)
Au9-Au40-Au35	120.14(12)	Au2-Au40-Au35	61.72(9)
Au42-Au40-Au35	61.04(9)	Au49-Au40-Au35	116.84(11)
Au10-Au40-Au35	118.59(11)	Au3-Au40-Au35	56.50(8)
Au1-Au40-Au35	88.79(10)	Au36-Au40-Au37	92.66(11)
Au9-Au40-Au37	59.44(9)	Au2-Au40-Au37	117.79(12)
Au42-Au40-Au37	119.98(11)	Au49-Au40-Au37	64.13(9)
Au10-Au40-Au37	56.60(9)	Au3-Au40-Au37	118.64(11)

Au1-Au40-Au37	86.60(10)	Au35-Au40-Au37	174.71(12)
Au36-Au40-Au75	57.49(9)	Au9-Au40-Au75	88.51(10)
Au2-Au40-Au75	58.87(9)	Au42-Au40-Au75	87.39(10)
Au49-Au40-Au75	116.15(11)	Au10-Au40-Au75	177.92(12)
Au3-Au40-Au75	118.44(11)	Au1-Au40-Au75	119.30(11)
Au35-Au40-Au75	61.98(9)	Au37-Au40-Au75	122.74(11)
Au36-Au40-Au53	56.18(9)	Au9-Au40-Au53	59.27(9)
Au2-Au40-Au53	90.47(10)	Au42-Au40-Au53	115.16(11)
Au49-Au40-Au53	86.67(10)	Au10-Au40-Au53	117.14(10)
Au3-Au40-Au53	178.98(12)	Au1-Au40-Au53	119.77(11)
Au35-Au40-Au53	124.27(11)	Au37-Au40-Au53	60.57(8)
Au75-Au40-Au53	62.30(8)	S30-Au41-S11	172.5(6)
S30-Au41-Au32	98.8(5)	S11-Au41-Au32	78.0(4)
S30-Au41-Au21	86.9(4)	S11-Au41-Au21	100.3(4)
Au32-Au41-Au21	101.61(11)	S30-Au41-Au46	45.2(5)
S11-Au41-Au46	133.3(4)	Au32-Au41-Au46	55.94(10)
Au21-Au41-Au46	83.35(10)	S30-Au41-Au19	139.3(5)
S11-Au41-Au19	47.6(4)	Au32-Au41-Au19	84.04(10)
Au21-Au41-Au19	53.12(8)	Au46-Au41-Au19	113.77(11)
S30-Au41-Au30	96.7(5)	S11-Au41-Au30	86.6(4)
Au32-Au41-Au30	50.93(8)	Au21-Au41-Au30	50.75(8)
Au46-Au41-Au30	59.77(9)	Au19-Au41-Au30	54.09(8)
S28-Au42-Au49	149.8(4)	S28-Au42-Au36	139.1(4)
Au49-Au42-Au36	60.68(10)	S28-Au42-Au40	143.3(4)
Au49-Au42-Au40	62.44(10)	Au36-Au42-Au40	57.63(9)
S28-Au42-Au35	83.7(4)	Au49-Au42-Au35	123.42(13)
Au36-Au42-Au35	91.28(11)	Au40-Au42-Au35	61.08(9)
S28-Au42-Au63	99.8(4)	Au49-Au42-Au63	61.25(10)
Au36-Au42-Au63	120.07(12)	Au40-Au42-Au63	83.46(10)
Au35-Au42-Au63	108.70(11)	S28-Au42-Au3	94.1(4)
Au49-Au42-Au3	92.60(11)	Au36-Au42-Au3	115.50(12)
Au40-Au42-Au3	57.97(8)	Au35-Au42-Au3	54.20(8)
Au63-Au42-Au3	54.51(8)	S28-Au42-Au61	88.5(4)
Au49-Au42-Au61	63.34(10)	Au36-Au42-Au61	97.31(11)
Au40-Au42-Au61	125.71(11)	Au35-Au42-Au61	171.17(13)
Au63-Au42-Au61	68.64(9)	Au3-Au42-Au61	122.71(11)
S28-Au42-Au65	44.1(4)	Au49-Au42-Au65	119.98(12)
Au36-Au42-Au65	172.76(13)	Au40-Au42-Au65	115.67(11)
Au35-Au42-Au65	82.52(10)	Au63-Au42-Au65	59.13(9)
Au3-Au42-Au65	57.70(8)	Au61-Au42-Au65	89.04(10)
S32-Au43-S34	172.4(6)	S32-Au43-Au15	82.0(4)
S34-Au43-Au15	100.8(4)	S32-Au43-Au35	99.0(4)
S34-Au43-Au35	86.9(4)	Au15-Au43-Au35	107.55(11)
S32-Au43-Au4	91.1(4)	S34-Au43-Au4	96.3(4)
Au15-Au43-Au4	53.70(8)	Au35-Au43-Au4	53.85(8)

S32-Au43-Au34	136.6(4)	S34-Au43-Au34	48.1(4)
Au15-Au43-Au34	55.77(9)	Au35-Au43-Au34	86.06(10)
Au4-Au43-Au34	57.47(8)	S32-Au43-Au2	46.8(4)
S34-Au43-Au2	140.1(4)	Au15-Au43-Au2	84.51(10)
Au35-Au43-Au2	54.36(8)	Au4-Au43-Au2	55.23(8)
Au34-Au43-Au2	112.70(11)	S24-Au44-S17	178.1(6)
S24-Au44-Au37	83.0(4)	S17-Au44-Au37	98.3(4)
S24-Au44-Au23	100.5(4)	S17-Au44-Au23	77.8(4)
Au37-Au44-Au23	104.30(11)	S24-Au44-Au8	92.8(4)
S17-Au44-Au8	86.9(4)	Au37-Au44-Au8	52.56(8)
Au23-Au44-Au8	51.74(8)	S24-Au44-Au39	47.1(4)
S17-Au44-Au39	131.5(4)	Au37-Au44-Au39	84.90(10)
Au23-Au44-Au39	54.91(8)	Au8-Au44-Au39	56.96(8)
S24-Au44-Au9	135.4(4)	S17-Au44-Au9	45.6(4)
Au37-Au44-Au9	53.33(8)	Au23-Au44-Au9	83.86(9)
Au8-Au44-Au9	55.29(8)	Au39-Au44-Au9	112.24(10)
S23-Au45-Au37	127.0(4)	S23-Au45-Au10	160.6(4)
Au37-Au45-Au10	61.52(9)	S23-Au45-Au8	137.7(4)
Au37-Au45-Au8	60.07(9)	Au10-Au45-Au8	61.24(9)
S23-Au45-Au62	106.4(4)	Au37-Au45-Au62	125.72(12)
Au10-Au45-Au62	64.75(9)	Au8-Au45-Au62	88.03(10)
S23-Au45-Au60	93.7(4)	Au37-Au45-Au60	96.91(11)
Au10-Au45-Au60	67.16(10)	Au8-Au45-Au60	128.37(12)
Au62-Au45-Au60	68.34(10)	S23-Au45-Au24	96.5(4)
Au37-Au45-Au24	119.02(11)	Au10-Au45-Au24	92.03(10)
Au8-Au45-Au24	59.06(9)	Au62-Au45-Au24	55.34(8)
Au60-Au45-Au24	123.39(12)	S23-Au45-Au39	74.9(4)
Au37-Au45-Au39	93.54(11)	Au10-Au45-Au39	123.92(11)
Au8-Au45-Au39	62.81(9)	Au62-Au45-Au39	110.23(11)
Au60-Au45-Au39	167.78(12)	Au24-Au45-Au39	55.29(8)
S23-Au45-Au58	45.7(4)	Au37-Au45-Au58	172.73(13)
Au10-Au45-Au58	124.99(12)	Au8-Au45-Au58	124.84(11)
Au62-Au45-Au58	61.27(9)	Au60-Au45-Au58	83.80(10)
Au24-Au45-Au58	65.82(9)	Au39-Au45-Au58	85.04(9)
S9-Au46-S30	171.4(7)	S9-Au46-Au32	80.6(6)
S30-Au46-Au32	98.0(4)	S9-Au46-Au26	99.6(6)
S30-Au46-Au26	89.0(4)	Au32-Au46-Au26	102.79(12)
S9-Au46-Au41	136.3(6)	S30-Au46-Au41	44.2(4)
Au32-Au46-Au41	56.07(10)	Au26-Au46-Au41	85.90(11)
S9-Au46-Au30	88.6(6)	S30-Au46-Au30	97.1(4)
Au32-Au46-Au30	51.48(8)	Au26-Au46-Au30	51.34(8)
Au41-Au46-Au30	61.18(9)	S9-Au46-Au27	47.0(5)
S30-Au46-Au27	141.5(4)	Au32-Au46-Au27	84.28(10)
Au26-Au46-Au27	53.48(9)	Au41-Au46-Au27	115.86(11)
Au30-Au46-Au27	54.71(8)	S27-Au47-Au34	132.1(5)

S27-Au47-Au16	162.7(5)	Au34-Au47-Au16	62.32(10)
S27-Au47-Au17	130.3(5)	Au34-Au47-Au17	59.00(10)
Au16-Au47-Au17	62.97(10)	S27-Au47-Au48	50.6(4)
Au34-Au47-Au48	95.23(12)	Au16-Au47-Au48	145.71(14)
Au17-Au47-Au48	83.51(12)	S27-Au47-Au70	92.9(5)
Au34-Au47-Au70	99.32(12)	Au16-Au47-Au70	73.45(10)
Au17-Au47-Au70	136.37(13)	Au48-Au47-Au70	139.05(13)
S27-Au47-Au72	94.3(5)	Au34-Au47-Au72	133.29(13)
Au16-Au47-Au72	71.04(10)	Au17-Au47-Au72	98.16(12)
Au48-Au47-Au72	124.16(13)	Au70-Au47-Au72	68.38(10)
S35-Au48-S27	172.5(8)	S35-Au48-Au47	122.8(5)
S27-Au48-Au47	54.3(4)	S21-Au49-Au42	157.7(4)
S21-Au49-Au36	134.5(4)	Au42-Au49-Au36	60.74(10)
S21-Au49-Au40	137.9(4)	Au42-Au49-Au40	61.67(10)
Au36-Au49-Au40	57.40(9)	S21-Au49-Au63	102.0(4)
Au42-Au49-Au63	64.68(10)	Au36-Au49-Au63	123.43(12)
Au40-Au49-Au63	84.74(10)	S21-Au49-Au37	78.6(3)
Au42-Au49-Au37	121.63(12)	Au36-Au49-Au37	89.34(11)
Au40-Au49-Au37	60.01(9)	Au63-Au49-Au37	107.44(11)
S21-Au49-Au10	92.6(4)	Au42-Au49-Au10	93.08(11)
Au36-Au49-Au10	114.75(12)	Au40-Au49-Au10	57.60(8)
Au63-Au49-Au10	54.26(8)	Au37-Au49-Au10	53.26(8)
S21-Au49-Au61	91.1(4)	Au42-Au49-Au61	68.05(10)
Au36-Au49-Au61	100.22(11)	Au40-Au49-Au61	129.64(12)
Au63-Au49-Au61	71.41(10)	Au37-Au49-Au61	169.23(12)
Au10-Au49-Au61	125.09(11)	S21-Au49-Au60	44.9(4)
Au42-Au49-Au60	122.46(12)	Au36-Au49-Au60	173.01(13)
Au40-Au49-Au60	117.60(11)	Au63-Au49-Au60	58.32(9)
Au37-Au49-Au60	83.74(9)	Au10-Au49-Au60	60.00(8)
Au61-Au49-Au60	86.75(10)	S18-Au50-S38	167.8(7)
S18-Au50-Au36	54.2(4)	S38-Au50-Au36	124.9(5)
S25-Au51-Au24	165.3(4)	S25-Au51-Au38	128.8(4)
Au24-Au51-Au38	63.55(10)	S25-Au51-Au39	128.7(4)
Au24-Au51-Au39	62.75(10)	Au38-Au51-Au39	58.76(10)
S25-Au51-Au52	49.1(4)	Au24-Au51-Au52	145.60(13)
Au38-Au51-Au52	86.00(11)	Au39-Au51-Au52	88.52(11)
S25-Au51-Au58	94.1(4)	Au24-Au51-Au58	74.00(10)
Au38-Au51-Au58	137.12(13)	Au39-Au51-Au58	97.18(11)
Au52-Au51-Au58	131.66(13)	S25-Au51-Au59	98.7(4)
Au24-Au51-Au59	70.00(10)	Au38-Au51-Au59	96.83(11)
Au39-Au51-Au59	132.55(13)	Au52-Au51-Au59	133.07(13)
Au58-Au51-Au59	73.04(10)	S25-Au52-S26	166.7(7)
S25-Au52-Au51	55.0(4)	S26-Au52-Au51	123.4(5)
S22-Au53-S19	172.8(6)	S22-Au53-Au36	80.4(4)
S19-Au53-Au36	93.7(4)	S22-Au53-Au9	102.0(4)

S19-Au53-Au9	83.1(4)	Au36-Au53-Au9	101.57(11)
S22-Au53-Au37	48.1(4)	S19-Au53-Au37	136.0(4)
Au36-Au53-Au37	85.14(10)	Au9-Au53-Au37	54.42(8)
S22-Au53-Au40	88.2(4)	S19-Au53-Au40	91.2(4)
Au36-Au53-Au40	50.84(8)	Au9-Au53-Au40	50.93(8)
Au37-Au53-Au40	54.74(8)	S12-Au54-S10	177.3(6)
S12-Au54-Au19	77.5(4)	S10-Au54-Au19	99.9(4)
S12-Au54-Au15	102.8(4)	S10-Au54-Au15	76.9(4)
Au19-Au54-Au15	105.20(11)	S12-Au54-Au14	91.0(4)
S10-Au54-Au14	86.7(4)	Au19-Au54-Au14	53.16(8)
Au15-Au54-Au14	52.04(8)	S12-Au54-Au17	47.9(4)
S10-Au54-Au17	131.3(4)	Au19-Au54-Au17	85.60(10)
Au15-Au54-Au17	55.28(8)	Au14-Au54-Au17	58.20(8)
S12-Au54-Au21	130.5(4)	S10-Au54-Au21	46.9(4)
Au19-Au54-Au21	53.68(8)	Au15-Au54-Au21	84.43(10)
Au14-Au54-Au21	55.11(8)	Au17-Au54-Au21	113.28(10)
S20-Au55-S35	172.9(7)	S20-Au55-Au17	92.7(4)
S35-Au55-Au17	91.8(5)	S40-Au56-S39	174.9(9)
S40-Au56-Au32	127.8(5)	S39-Au56-Au32	53.1(4)
S36-Au57-Au35	129.8(4)	S36-Au57-Au3	159.6(4)
Au35-Au57-Au3	60.73(10)	S36-Au57-Au4	137.4(4)
Au35-Au57-Au4	62.34(9)	Au3-Au57-Au4	61.52(9)
S36-Au57-Au68	102.9(4)	Au35-Au57-Au68	125.95(12)
Au3-Au57-Au68	65.40(9)	Au4-Au57-Au68	88.88(10)
S36-Au57-Au65	94.5(4)	Au35-Au57-Au65	94.48(11)
Au3-Au57-Au65	65.82(10)	Au4-Au57-Au65	127.26(12)
Au68-Au57-Au65	66.58(10)	S36-Au57-Au16	94.6(4)
Au35-Au57-Au16	121.54(12)	Au3-Au57-Au16	91.55(10)
Au4-Au57-Au16	59.21(9)	Au68-Au57-Au16	54.72(9)
Au65-Au57-Au16	121.17(12)	S36-Au57-Au34	75.2(4)
Au35-Au57-Au34	96.76(11)	Au3-Au57-Au34	123.62(12)
Au4-Au57-Au34	62.31(9)	Au68-Au57-Au34	109.27(11)
Au65-Au57-Au34	168.09(12)	Au16-Au57-Au34	55.07(9)
S36-Au57-Au70	45.1(4)	Au35-Au57-Au70	173.90(13)
Au3-Au57-Au70	122.87(12)	Au4-Au57-Au70	123.42(11)
Au68-Au57-Au70	58.31(9)	Au65-Au57-Au70	83.32(10)
Au16-Au57-Au70	64.22(9)	Au34-Au57-Au70	85.10(10)
S1-Au58-S23	175.6(6)	S1-Au58-Au51	93.3(4)
S23-Au58-Au51	82.4(4)	S1-Au58-Au62	78.9(4)
S23-Au58-Au62	102.9(4)	Au51-Au58-Au62	100.16(11)
S1-Au58-Au45	132.0(4)	S23-Au58-Au45	49.0(4)
Au51-Au58-Au45	85.36(10)	Au62-Au58-Au45	54.45(8)
S15-Au59-S1	167.8(5)	S15-Au59-Au51	100.1(4)
S1-Au59-Au51	91.7(4)	S15-Au59-Au64	94.5(4)
S1-Au59-Au64	85.5(4)	Au51-Au59-Au64	103.83(11)

S15-Au59-Au29	47.8(4)	S1-Au59-Au29	138.0(4)
Au51-Au59-Au29	83.10(10)	Au64-Au59-Au29	55.95(8)
S15-Au59-Au24	98.8(4)	S1-Au59-Au24	90.9(4)
Au51-Au59-Au24	51.61(8)	Au64-Au59-Au24	52.35(8)
Au29-Au59-Au24	53.72(8)	S2-Au60-S21	179.7(7)
S2-Au60-Au45	98.7(4)	S21-Au60-Au45	81.4(4)
S2-Au60-Au63	78.8(4)	S21-Au60-Au63	101.5(4)
Au45-Au60-Au63	106.05(11)	S2-Au60-Au10	87.2(4)
S21-Au60-Au10	93.1(4)	Au45-Au60-Au10	53.36(8)
Au63-Au60-Au10	52.69(8)	S2-Au60-Au49	132.7(4)
S21-Au60-Au49	47.6(4)	Au45-Au60-Au49	85.84(10)
Au63-Au60-Au49	55.13(8)	Au10-Au60-Au49	58.08(8)
S2-Au60-Au62	45.4(4)	S21-Au60-Au62	134.8(4)
Au45-Au60-Au62	54.19(8)	Au63-Au60-Au62	85.07(10)
Au10-Au60-Au62	55.64(8)	Au49-Au60-Au62	113.71(10)
S38-Au61-S37	171.5(7)	S38-Au61-Au49	94.8(4)
S37-Au61-Au49	90.1(4)	S38-Au61-Au42	109.3(5)
S37-Au61-Au42	79.2(4)	Au49-Au61-Au42	48.60(8)
S2-Au62-Au64	132.1(4)	S2-Au62-Au24	160.0(4)
Au64-Au62-Au24	60.93(10)	S2-Au62-Au45	100.7(4)
Au64-Au62-Au45	126.14(12)	Au24-Au62-Au45	65.36(9)
S2-Au62-Au6	133.7(4)	Au64-Au62-Au6	61.70(9)
Au24-Au62-Au6	63.78(9)	Au45-Au62-Au6	91.69(10)
S2-Au62-Au11	74.9(4)	Au64-Au62-Au11	95.24(11)
Au24-Au62-Au11	122.46(11)	Au45-Au62-Au11	110.23(11)
Au6-Au62-Au11	58.96(9)	S2-Au62-Au10	90.3(4)
Au64-Au62-Au10	121.99(12)	Au24-Au62-Au10	92.87(10)
Au45-Au62-Au10	56.18(8)	Au6-Au62-Au10	60.29(8)
Au11-Au62-Au10	54.31(8)	S2-Au62-Au58	90.9(4)
Au64-Au62-Au58	100.05(11)	Au24-Au62-Au58	70.38(10)
Au45-Au62-Au58	64.28(9)	Au6-Au62-Au58	133.92(12)
Au11-Au62-Au58	163.86(12)	Au10-Au62-Au58	119.54(11)
S2-Au62-Au60	44.1(4)	Au64-Au62-Au60	176.17(13)
Au24-Au62-Au60	122.54(12)	Au45-Au62-Au60	57.47(9)
Au6-Au62-Au60	120.74(11)	Au11-Au62-Au60	84.11(9)
Au10-Au62-Au60	60.55(8)	Au58-Au62-Au60	80.29(9)
S37-Au63-Au11	135.4(5)	S37-Au63-Au10	158.4(5)
Au11-Au63-Au10	60.34(9)	S37-Au63-Au3	132.7(5)
Au11-Au63-Au3	60.63(9)	Au10-Au63-Au3	65.12(9)
S37-Au63-Au49	96.3(5)	Au11-Au63-Au49	127.18(12)
Au10-Au63-Au49	66.94(10)	Au3-Au63-Au49	95.00(11)
S37-Au63-Au42	85.5(5)	Au11-Au63-Au42	125.66(12)
Au10-Au63-Au42	94.50(11)	Au3-Au63-Au42	65.12(9)
Au49-Au63-Au42	54.07(9)	S37-Au63-Au60	94.4(5)
Au11-Au63-Au60	94.70(11)	Au10-Au63-Au60	66.92(9)

Au3-Au63-Au60	132.03(12)	Au49-Au63-Au60	66.55(10)
Au42-Au63-Au60	120.12(12)	S37-Au63-Au65	71.4(4)
Au11-Au63-Au65	91.78(11)	Au10-Au63-Au65	128.19(12)
Au3-Au63-Au65	63.12(9)	Au49-Au63-Au65	119.56(12)
Au42-Au63-Au65	65.88(9)	Au60-Au63-Au65	164.59(12)
S7-Au64-Au62	133.0(4)	S7-Au64-Au24	155.3(4)
Au62-Au64-Au24	60.39(9)	S7-Au64-Au6	138.6(4)
Au62-Au64-Au6	62.27(9)	Au24-Au64-Au6	63.81(9)
S7-Au64-Au29	101.7(4)	Au62-Au64-Au29	122.25(12)
Au24-Au64-Au29	61.91(9)	Au6-Au64-Au29	90.58(10)
S7-Au64-Au67	79.2(4)	Au62-Au64-Au67	96.44(11)
Au24-Au64-Au67	123.19(12)	Au6-Au64-Au67	59.65(9)
Au29-Au64-Au67	112.77(11)	S7-Au64-Au25	92.5(4)
Au62-Au64-Au25	123.53(12)	Au24-Au64-Au25	93.08(11)
Au6-Au64-Au25	61.28(9)	Au29-Au64-Au25	57.43(9)
Au67-Au64-Au25	55.38(9)	S7-Au64-Au59	88.2(4)
Au62-Au64-Au59	94.55(11)	Au24-Au64-Au59	68.64(10)
Au6-Au64-Au59	132.45(12)	Au29-Au64-Au59	66.40(9)
Au67-Au64-Au59	166.99(12)	Au25-Au64-Au59	122.70(11)
S7-Au64-Au66	43.6(4)	Au62-Au64-Au66	176.27(13)
Au24-Au64-Au66	121.86(11)	Au6-Au64-Au66	121.19(11)
Au29-Au64-Au66	60.21(9)	Au67-Au64-Au66	84.69(10)
Au25-Au64-Au66	59.98(9)	Au59-Au64-Au66	83.92(10)
S4-Au65-S28	170.6(6)	S4-Au65-Au57	102.7(4)
S28-Au65-Au57	86.6(4)	S4-Au65-Au3	91.0(4)
S28-Au65-Au3	95.7(4)	Au57-Au65-Au3	54.41(8)
S4-Au65-Au63	81.2(4)	S28-Au65-Au63	97.5(4)
Au57-Au65-Au63	107.56(11)	Au3-Au65-Au63	53.22(8)
S4-Au65-Au68	48.6(4)	S28-Au65-Au68	140.8(4)
Au57-Au65-Au68	54.96(8)	Au3-Au65-Au68	57.60(8)
Au63-Au65-Au68	88.39(10)	S4-Au65-Au42	135.4(4)
S28-Au65-Au42	45.9(4)	Au57-Au65-Au42	84.93(10)
Au3-Au65-Au42	57.74(8)	Au63-Au65-Au42	54.98(8)
Au68-Au65-Au42	115.25(11)	S42-Au66-S7	175.1(7)
S42-Au66-Au71	103.2(6)	S7-Au66-Au71	81.5(4)
S42-Au66-Au29	82.3(6)	S7-Au66-Au29	97.9(4)
Au71-Au66-Au29	107.01(12)	S42-Au66-Au25	94.1(5)
S7-Au66-Au25	90.0(4)	Au71-Au66-Au25	53.30(9)
Au29-Au66-Au25	53.72(9)	S42-Au66-Au31	47.9(5)
S7-Au66-Au31	137.1(4)	Au71-Au66-Au31	56.58(9)
Au29-Au66-Au31	87.17(11)	Au25-Au66-Au31	59.10(9)
S42-Au66-Au64	135.6(6)	S7-Au66-Au64	45.8(4)
Au71-Au66-Au64	83.97(10)	Au29-Au66-Au64	54.11(8)
Au25-Au66-Au64	55.07(8)	Au31-Au66-Au64	114.17(11)
S3-Au67-Au71	135.3(4)	S3-Au67-Au25	160.5(5)

Au71-Au67-Au25	60.93(10)	S3-Au67-Au20	129.6(5)
Au71-Au67-Au20	60.32(10)	Au25-Au67-Au20	64.90(9)
S3-Au67-Au6	99.5(4)	Au71-Au67-Au6	124.58(12)
Au25-Au67-Au6	63.70(9)	Au20-Au67-Au6	94.41(11)
S3-Au67-Au64	100.4(5)	Au71-Au67-Au64	96.78(11)
Au25-Au67-Au64	62.98(9)	Au20-Au67-Au64	127.72(12)
Au6-Au67-Au64	58.76(9)	S3-Au67-Au13	84.0(4)
Au71-Au67-Au13	122.30(12)	Au25-Au67-Au13	94.91(11)
Au20-Au67-Au13	61.98(9)	Au6-Au67-Au13	60.15(8)
Au64-Au67-Au13	118.64(11)	S3-Au67-Au69	69.8(5)
Au71-Au67-Au69	94.14(11)	Au25-Au67-Au69	125.43(12)
Au20-Au67-Au69	60.66(9)	Au6-Au67-Au69	116.27(11)
Au64-Au67-Au69	168.79(12)	Au13-Au67-Au69	56.35(8)
S3-Au67-Au11	45.1(4)	Au71-Au67-Au11	177.92(13)
Au25-Au67-Au11	119.17(11)	Au20-Au67-Au11	117.69(11)
Au6-Au67-Au11	55.47(8)	Au64-Au67-Au11	84.97(9)
Au13-Au67-Au11	55.71(8)	Au69-Au67-Au11	84.17(9)
S4-Au68-Au69	128.5(4)	S4-Au68-Au16	163.6(4)
Au69-Au68-Au16	61.44(10)	S4-Au68-Au57	103.2(4)
Au69-Au68-Au57	127.69(12)	Au16-Au68-Au57	66.39(10)
S4-Au68-Au13	131.9(4)	Au69-Au68-Au13	61.90(9)
Au16-Au68-Au13	62.98(9)	Au57-Au68-Au13	91.60(11)
S4-Au68-Au70	92.7(4)	Au69-Au68-Au70	98.31(11)
Au16-Au68-Au70	71.96(10)	Au57-Au68-Au70	68.23(10)
Au13-Au68-Au70	134.92(12)	S4-Au68-Au3	90.9(4)
Au69-Au68-Au3	121.82(12)	Au16-Au68-Au3	93.06(11)
Au57-Au68-Au3	56.38(9)	Au13-Au68-Au3	59.94(9)
Au70-Au68-Au3	123.76(12)	S4-Au68-Au11	74.2(4)
Au69-Au68-Au11	93.77(11)	Au16-Au68-Au11	120.54(11)
Au57-Au68-Au11	109.50(11)	Au13-Au68-Au11	57.79(8)
Au70-Au68-Au11	165.99(12)	Au3-Au68-Au11	53.30(8)
S4-Au68-Au65	45.6(4)	Au69-Au68-Au65	173.84(13)
Au16-Au68-Au65	124.71(12)	Au57-Au68-Au65	58.46(9)
Au13-Au68-Au65	119.93(11)	Au70-Au68-Au65	84.24(10)
Au3-Au68-Au65	60.18(8)	Au11-Au68-Au65	83.06(9)
S5-Au69-Au68	132.2(4)	S5-Au69-Au16	159.3(4)
Au68-Au69-Au16	59.48(9)	S5-Au69-Au18	101.9(4)
Au68-Au69-Au18	123.72(13)	Au16-Au69-Au18	64.25(10)
S5-Au69-Au13	136.1(4)	Au68-Au69-Au13	62.35(9)
Au16-Au69-Au13	62.53(9)	Au18-Au69-Au13	91.34(11)
S5-Au69-Au20	92.2(4)	Au68-Au69-Au20	123.09(12)
Au16-Au69-Au20	92.13(11)	Au18-Au69-Au20	56.62(9)
Au13-Au69-Au20	60.78(9)	S5-Au69-Au72	91.8(4)
Au68-Au69-Au72	92.83(11)	Au16-Au69-Au72	69.06(10)
Au18-Au69-Au72	67.71(10)	Au13-Au69-Au72	131.58(12)

Au20-Au69-Au72	123.79(12)	S5-Au69-Au67	77.0(4)
Au68-Au69-Au67	97.20(11)	Au16-Au69-Au67	121.47(12)
Au18-Au69-Au67	110.90(11)	Au13-Au69-Au67	59.27(9)
Au20-Au69-Au67	54.43(8)	Au72-Au69-Au67	168.30(12)
S5-Au69-Au73	44.7(4)	Au68-Au69-Au73	175.23(13)
Au16-Au69-Au73	122.11(12)	Au18-Au69-Au73	58.18(9)
Au13-Au69-Au73	122.41(11)	Au20-Au69-Au73	61.68(9)
Au72-Au69-Au73	83.93(10)	Au67-Au69-Au73	85.67(9)
S6-Au70-S36	174.4(6)	S6-Au70-Au47	98.5(5)
S36-Au70-Au47	79.3(4)	S6-Au70-Au68	86.3(5)
S36-Au70-Au68	99.2(4)	Au47-Au70-Au68	100.66(11)
S6-Au70-Au72	44.3(4)	S36-Au70-Au72	134.8(4)
Au47-Au70-Au72	56.30(9)	Au68-Au70-Au72	82.62(10)
S6-Au70-Au57	139.0(5)	S36-Au70-Au57	46.3(4)
Au47-Au70-Au57	83.27(10)	Au68-Au70-Au57	53.46(8)
Au72-Au70-Au57	113.67(10)	S41-Au71-Au67	131.6(5)
S41-Au71-Au25	147.1(6)	Au67-Au71-Au25	60.26(9)
S41-Au71-Au20	146.7(6)	Au67-Au71-Au20	61.49(9)
Au25-Au71-Au20	65.09(9)	S41-Au71-Au33	95.1(5)
Au67-Au71-Au33	127.58(13)	Au25-Au71-Au33	95.01(11)
Au20-Au71-Au33	66.21(10)	S41-Au71-Au31	95.4(6)
Au67-Au71-Au31	126.37(13)	Au25-Au71-Au31	66.15(10)
Au20-Au71-Au31	94.77(11)	Au33-Au71-Au31	54.09(9)
S41-Au71-Au66	82.0(6)	Au67-Au71-Au66	94.22(12)
Au25-Au71-Au66	65.61(10)	Au20-Au71-Au66	130.69(12)
Au33-Au71-Au66	117.93(12)	Au31-Au71-Au66	64.45(10)
S41-Au71-Au73	81.6(6)	Au67-Au71-Au73	95.67(11)
Au25-Au71-Au73	130.85(12)	Au20-Au71-Au73	65.76(10)
Au33-Au71-Au73	65.02(10)	Au31-Au71-Au73	118.55(12)
Au66-Au71-Au73	163.54(13)	S6-Au72-S13	171.3(6)
S6-Au72-Au47	96.9(5)	S13-Au72-Au47	79.6(4)
S6-Au72-Au69	88.5(4)	S13-Au72-Au69	100.0(4)
Au47-Au72-Au69	102.93(11)	S6-Au72-Au18	140.5(4)
S13-Au72-Au18	47.6(4)	Au47-Au72-Au18	84.51(10)
Au69-Au72-Au18	53.16(8)	S6-Au72-Au70	43.7(5)
S13-Au72-Au70	134.6(4)	Au47-Au72-Au70	55.32(9)
Au69-Au72-Au70	86.24(10)	Au18-Au72-Au70	115.46(11)
S6-Au72-Au16	96.8(4)	S13-Au72-Au16	87.3(4)
Au47-Au72-Au16	51.45(8)	Au69-Au72-Au16	51.56(8)
Au18-Au72-Au16	53.88(8)	Au70-Au72-Au16	61.62(8)
S5-Au73-S14	179.6(7)	S5-Au73-Au18	100.3(4)
S14-Au73-Au18	80.0(4)	S5-Au73-Au71	79.1(5)
S14-Au73-Au71	100.5(4)	Au18-Au73-Au71	104.62(11)
S5-Au73-Au20	89.1(4)	S14-Au73-Au20	90.8(4)
Au18-Au73-Au20	52.90(8)	Au71-Au73-Au20	51.72(8)

S5-Au73-Au33	132.8(5)	S14-Au73-Au33	46.9(4)
Au18-Au73-Au33	85.60(10)	Au71-Au73-Au33	54.41(8)
Au20-Au73-Au33	57.44(8)	S5-Au73-Au69	47.6(4)
S14-Au73-Au69	132.6(4)	Au18-Au73-Au69	53.68(8)
Au71-Au73-Au69	84.01(10)	Au20-Au73-Au69	55.24(8)
Au33-Au73-Au69	112.68(11)	S40-Au74-S41	167.7(8)
S40-Au74-Au33	96.2(6)	S41-Au74-Au33	90.6(5)
S19-Au75-S33	169.0(6)	S19-Au75-Au36	92.6(4)
S33-Au75-Au36	97.9(5)	S19-Au75-Au2	85.6(4)
S33-Au75-Au2	95.3(4)	Au36-Au75-Au2	101.97(11)
S19-Au75-Au35	138.3(4)	S33-Au75-Au35	47.3(4)
Au36-Au75-Au35	82.21(10)	Au2-Au75-Au35	55.72(8)
S19-Au75-Au40	91.8(4)	S33-Au75-Au40	97.4(4)
Au36-Au75-Au40	50.68(8)	Au2-Au75-Au40	51.43(8)
Au35-Au75-Au40	53.44(8)	S26-Au76-S29	162.0(9)
S26-Au76-Au38	103.1(5)	S29-Au76-Au38	89.0(5)
S26-Au76-Au39	95.3(5)	S29-Au76-Au39	82.9(6)
Au38-Au76-Au39	47.69(8)	C11-S1-Au58	105.3(14)
C11-S1-Au59	106.5(17)	Au58-S1-Au59	99.2(6)
C21-S2-Au60	107.6(15)	C21-S2-Au62	117.0(13)
Au60-S2-Au62	90.6(6)	C31-S3-Au11	122.6(13)
C31-S3-Au67	118.8(12)	Au11-S3-Au67	89.3(6)
C41-S4-Au65	111.(2)	C41-S4-Au68	116.1(16)
Au65-S4-Au68	85.8(5)	C51-S5-Au73	109.(2)
C51-S5-Au69	115.0(17)	Au73-S5-Au69	87.7(6)
C61-S6-Au70	104.(2)	C61-S6-Au72	113.(3)
Au70-S6-Au72	92.0(6)	C71-S7-Au66	111.0(19)
C71-S7-Au64	114.1(15)	Au66-S7-Au64	90.6(6)
C81-S8-Au28	108.(2)	C81-S8-Au26	113.2(16)
Au28-S8-Au26	87.3(5)	C91-S9-Au46	112.(3)
C91-S9-Au27	117.(2)	Au46-S9-Au27	89.4(8)
C101-S10-Au54	105.7(18)	C101-S10-Au21	108.4(15)
Au54-S10-Au21	88.5(5)	C111-S11-Au41	106.5(18)
C111-S11-Au19	116.2(16)	Au41-S11-Au19	87.7(5)
C121-S12-Au54	109.9(16)	C121-S12-Au17	116.8(15)
Au54-S12-Au17	87.1(5)	C131-S13-Au72	113.5(18)
C131-S13-Au18	113.6(14)	Au72-S13-Au18	87.6(5)
C141-S14-Au73	108.3(18)	C141-S14-Au33	112.9(15)
Au73-S14-Au33	87.5(6)	C151-S15-Au59	113.4(16)
C151-S15-Au29	114.6(13)	Au59-S15-Au29	87.6(5)
C161-S16-Au28	110.3(17)	C161-S16-Au38	111.7(17)
Au28-S16-Au38	90.3(6)	C171-S17-Au44	109.6(18)
C171-S17-Au9	115.6(16)	Au44-S17-Au9	91.2(5)
C181-S18-Au50	108.2(17)	C181-S18-Au36	109.8(15)
Au50-S18-Au36	76.0(5)	C191-S19-Au75	107.6(17)

C191-S19-Au53	99.4(14)	Au75-S19-Au53	98.0(6)
C201-S20-Au55	107.(2)	C201-S20-Au15	111.2(19)
Au55-S20-Au15	99.8(7)	C211-S21-Au60	110.6(15)
C211-S21-Au49	109.4(13)	Au60-S21-Au49	87.6(5)
C221-S22-Au53	110.6(15)	C221-S22-Au37	109.9(13)
Au53-S22-Au37	86.8(6)	C231-S23-Au58	113.7(17)
C231-S23-Au45	112.7(14)	Au58-S23-Au45	85.3(5)
C241-S24-Au44	110.9(18)	C241-S24-Au39	110.3(15)
Au44-S24-Au39	88.6(5)	C251-S25-Au52	102.9(15)
C251-S25-Au51	106.4(14)	Au52-S25-Au51	75.9(5)
C261-S26-Au76	116.(2)	C261-S26-Au52	110.(2)
Au76-S26-Au52	107.7(9)	C271-S27-Au48	109.8(19)
C271-S27-Au47	105.3(15)	Au48-S27-Au47	75.1(5)
C281-S28-Au65	104.8(18)	C281-S28-Au42	111.8(16)
Au65-S28-Au42	90.0(5)	C291-S29-Au76	121.(3)
C291-S29-Au23	121.(2)	Au76-S29-Au23	101.8(8)
C301-S30-Au41	106.(2)	C301-S30-Au46	98.(3)
Au41-S30-Au46	90.7(7)	C311-S31-Au5	128.(2)
C311-S31-Au7	122.(2)	Au5-S31-Au7	88.6(6)
C321-S32-Au43	108.0(19)	C321-S32-Au2	109.0(15)
Au43-S32-Au2	88.8(6)	C331-S33-Au75	112.5(18)
C331-S33-Au35	114.9(16)	Au75-S33-Au35	87.7(5)
C341-S34-Au43	104.0(18)	C341-S34-Au34	107.7(16)
Au43-S34-Au34	84.7(6)	C351-S35-Au48	111.(2)
C351-S35-Au55	111.(2)	Au48-S35-Au55	103.6(8)
C361-S36-Au70	108.2(16)	C361-S36-Au57	110.9(14)
Au70-S36-Au57	88.6(6)	C371-S37-Au61	105.(2)
C371-S37-Au63	119.4(19)	Au61-S37-Au63	101.4(7)
C381-S38-Au61	111.8(15)	C381-S38-Au50	110.4(10)
Au61-S38-Au50	102.4(7)	C391-S39-Au56	97.0(19)
C391-S39-Au32	110.(2)	Au56-S39-Au32	76.2(6)
C401-S40-Au74	107.(2)	C401-S40-Au56	103.(3)
Au74-S40-Au56	102.6(8)	C411_2-S41-Au74	118.6(14)
C411_1-S41-Au74	86.8(12)	C411_2-S41-Au71	124.8(12)
C411_1-S41-Au71	165.(2)	Au74-S41-Au71	98.6(8)
C421-S42-Au66	102.9(19)	C421-S42-Au31	122.7(15)
Au66-S42-Au31	87.4(7)	C12-C11-C16	120.00
C12-C11-S1	119.(3)	C16-C11-S1	121.(3)
C11-C12-C13	120.00	C14-C13-C12	120.00
C15-C14-C13	120.00	C15-C14-C17	115.(5)
C13-C14-C17	125.(5)	C16-C15-C14	120.00
C15-C16-C11	120.00	C22-C21-C26	120.00
C22-C21-S2	126.(3)	C26-C21-S2	113.(3)
C23-C22-C21	120.00	C22-C23-C24	120.00
C23-C24-C25	120.00	C23-C24-C27	110.(4)

C25-C24-C27	130.(4)	C26-C25-C24	120.00
C25-C26-C21	120.00	C32-C31-C36	120.00
C32-C31-S3	119.7(11)	C36-C31-S3	119.9(11)
C31-C32-C33	120.00	C34-C33-C32	120.00
C33-C34-C35	120.00	C33-C34-C37	117.(5)
C35-C34-C37	123.(5)	C36-C35-C34	120.00
C35-C36-C31	120.00	C46-C41-C43	120.00
C46-C41-S4	117.(4)	C43-C41-S4	122.(4)
C41-C46-C44	120.00	C45-C44-C46	120.00
C44-C45-C42	120.00	C44-C45-C47	112.(5)
C42-C45-C47	127.(5)	C43-C42-C45	120.00
C42-C43-C41	120.00	C52-C51-C56	120.00
C52-C51-S5	118.(5)	C56-C51-S5	122.(5)
C51-C52-C53	120.00	C54-C53-C52	120.00
C53-C54-C55	120.00	C53-C54-C57	114.(6)
C55-C54-C57	126.(6)	C54-C55-C56	120.00
C55-C56-C51	120.00	C62-C61-C66	120.00
C62-C61-S6	131.(4)	C66-C61-S6	109.(4)
C61-C62-C63	120.00	C64-C63-C62	120.00
C63-C64-C65	120.00	C63-C64-C67	119.4(13)
C65-C64-C67	119.3(13)	C64-C65-C66	120.00
C65-C66-C61	120.00	C72-C71-C76	120.00
C72-C71-S7	124.(3)	C76-C71-S7	116.(3)
C71-C72-C73	120.00	C72-C73-C74	120.00
C75-C74-C73	120.00	C75-C74-C77	127.(5)
C73-C74-C77	113.(5)	C74-C75-C76	120.00
C75-C76-C71	120.00	C82-C81-C86	120.00
C82-C81-S8	114.(4)	C86-C81-S8	126.(4)
C81-C82-C83	120.00	C82-C83-C84	120.00
C87-C84-C85	109.(6)	C87-C84-C83	131.(6)
C85-C84-C83	120.00	C84-C85-C86	120.00
C85-C86-C81	120.00	C92-C91-C96	120.00
C92-C91-S9	125.(4)	C96-C91-S9	115.(4)
C91-C92-C93	120.00	C94-C93-C92	120.00
C93-C94-C95	120.00	C93-C94-C97	120.2(12)
C95-C94-C97	119.7(12)	C94-C95-C96	120.00
C95-C96-C91	120.00	C102-C101-C106	120.00
C102-C101-S10	127.(3)	C106-C101-S10	112.(3)
C101-C102-C103	120.00	C104-C103-C102	120.00
C103-C104-C105	120.00	C103-C104-C107	122.(6)
C105-C104-C107	118.(6)	C104-C105-C106	120.00
C105-C106-C101	120.00	C112-C111-C116	120.00
C112-C111-S11	129.(3)	C116-C111-S11	111.(3)
C111-C112-C113	120.00	C114-C113-C112	120.00
C115-C114-C113	120.00	C115-C114-C117	119.(4)

C113-C114-C117	121.(4)	C114-C115-C116	120.00
C115-C116-C111	120.00	C122-C121-C126	120.00
C122-C121-S12	124.(3)	C126-C121-S12	116.(3)
C121-C122-C123	120.00	C124-C123-C122	120.00
C123-C124-C125	120.00	C123-C124-C127	119.8(12)
C125-C124-C127	120.0(11)	C126-C125-C124	120.00
C125-C126-C121	120.00	C132-C131-C136	120.00
C132-C131-S13	124.(3)	C136-C131-S13	116.(3)
C131-C132-C133	120.00	C132-C133-C134	120.00
C135-C134-C133	120.00	C135-C134-C137	128.(4)
C133-C134-C137	112.(4)	C136-C135-C134	120.00
C135-C136-C131	120.00	C142-C141-C146	120.00
C142-C141-S14	125.(3)	C146-C141-S14	115.(3)
C143-C142-C141	120.00	C142-C143-C144	120.00
C143-C144-C145	120.00	C143-C144-C147	121.(5)
C145-C144-C147	119.(5)	C146-C145-C144	120.00
C145-C146-C141	120.00	C152-C151-C156	120.00
C152-C151-S15	121.(3)	C156-C151-S15	118.(3)
C153-C152-C151	120.00	C152-C153-C154	120.00
C155-C154-C153	120.00	C155-C154-C157	119.(5)
C153-C154-C157	121.(5)	C154-C155-C156	120.00
C155-C156-C151	120.00	C162-C161-C166	120.00
C162-C161-S16	112.(4)	C166-C161-S16	127.(4)
C161-C162-C163	120.00	C164-C163-C162	120.00
C163-C164-C165	120.00	C163-C164-C167	125.(6)
C165-C164-C167	115.(6)	C166-C165-C164	120.00
C165-C166-C161	120.00	C172-C171-C176	120.00
C172-C171-S17	120.(4)	C176-C171-S17	120.(4)
C171-C172-C173	120.00	C174-C173-C172	120.00
C173-C174-C175	120.00	C173-C174-C177	117.(5)
C175-C174-C177	122.(5)	C174-C175-C176	120.00
C175-C176-C171	120.00	C182-C181-C186	120.00
C182-C181-S18	116.(3)	C186-C181-S18	124.(3)
C181-C182-C183	120.00	C184-C183-C182	120.00
C183-C184-C185	120.00	C183-C184-C187	120.(5)
C185-C184-C187	119.(5)	C186-C185-C184	120.00
C185-C186-C181	120.00	C192-C191-C196	120.00
C192-C191-S19	117.(3)	C196-C191-S19	122.(3)
C191-C192-C193	120.00	C194-C193-C192	120.00
C195-C194-C193	120.00	C195-C194-C197	109.(5)
C193-C194-C197	131.(5)	C194-C195-C196	120.00
C195-C196-C191	120.00	C202-C201-C206	120.00
C202-C201-S20	118.(4)	C206-C201-S20	122.(4)
C203-C202-C201	120.00	C202-C203-C204	120.00
C203-C204-C205	120.00	C203-C204-C207	113.(5)

C205-C204-C207	127.(5)	C206-C205-C204	120.00
C205-C206-C201	120.00	C212-C211-C216	120.00
C212-C211-S21	118.(3)	C216-C211-S21	122.(3)
C211-C212-C213	120.00	C212-C213-C214	120.00
C213-C214-C215	120.00	C213-C214-C217	117.(4)
C215-C214-C217	123.(4)	C216-C215-C214	120.00
C215-C216-C211	120.00	C222-C221-C226	120.00
C222-C221-S22	123.(3)	C226-C221-S22	117.(3)
C221-C222-C223	120.00	C222-C223-C224	120.00
C225-C224-C223	120.00	C225-C224-C227	118.(4)
C223-C224-C227	122.(4)	C224-C225-C226	120.00
C225-C226-C221	120.00	C232-C231-C236	120.00
C232-C231-S23	119.(3)	C236-C231-S23	121.(3)
C233-C232-C231	120.00	C232-C233-C234	120.00
C235-C234-C233	120.00	C235-C234-C237	121.(4)
C233-C234-C237	118.(4)	C236-C235-C234	120.00
C235-C236-C231	120.00	C242-C241-C246	120.00
C242-C241-S24	113.(3)	C246-C241-S24	127.(3)
C243-C242-C241	120.00	C242-C243-C244	120.00
C245-C244-C243	120.00	C245-C244-C247	112.(5)
C243-C244-C247	127.(5)	C244-C245-C246	120.00
C245-C246-C241	120.00	C252-C251-C256	120.00
C252-C251-S25	112.(2)	C256-C251-S25	128.(2)
C253-C252-C251	120.00	C252-C253-C254	120.00
C253-C254-C255	120.00	C253-C254-C257	118.9(11)
C255-C254-C257	121.1(11)	C256-C255-C254	120.00
C255-C256-C251	120.00	C262-C261-C266	120.00
C262-C261-S26	121.(4)	C266-C261-S26	117.(4)
C263-C262-C261	120.00	C264-C263-C262	120.00
C263-C264-C265	120.00	C263-C264-C267	115.(6)
C265-C264-C267	123.(6)	C264-C265-C266	120.00
C265-C266-C261	120.00	C272-C271-C276	120.00
C272-C271-S27	117.(3)	C276-C271-S27	123.(3)
C271-C272-C273	120.00	C274-C273-C272	120.00
C275-C274-C273	120.00	C275-C274-C277	113.(5)
C273-C274-C277	127.(5)	C274-C275-C276	120.00
C275-C276-C271	120.00	C282-C281-C286	120.00
C282-C281-S28	121.(3)	C286-C281-S28	119.(3)
C283-C282-C281	120.00	C282-C283-C284	120.00
C285-C284-C283	120.00	C285-C284-C287	120.(5)
C283-C284-C287	120.(5)	C286-C285-C284	120.00
C285-C286-C281	120.00	C292-C291-C296	120.00
C292-C291-S29	128.(5)	C296-C291-S29	111.(5)
C291-C292-C293	120.00	C294-C293-C292	120.00
C295-C294-C293	120.00	C295-C294-C297	117.3(16)

C293-C294-C297	118.3(16)	C294-C295-C296	120.00
C295-C296-C291	120.00	C302-C301-C306	120.00
C302-C301-S30	105.(8)	C306-C301-S30	135.(8)
C303-C302-C301	120.00	C302-C303-C304	120.00(5)
C305-C304-C303	120.00(6)	C305-C304-C307	117.(9)
C303-C304-C307	122.(9)	C304-C305-C306	120.00
C305-C306-C301	120.00	C312-C311-C316	120.00
C312-C311-S31	125.(4)	C316-C311-S31	114.(4)
C311-C312-C313	120.00	C314-C313-C312	120.00
C313-C314-C315	120.00	C313-C314-C317	118.(5)
C315-C314-C317	122.(5)	C316-C315-C314	120.00
C315-C316-C311	120.00	C322-C321-C326	120.00
C322-C321-S32	118.(3)	C326-C321-S32	121.(3)
C321-C322-C323	120.00	C322-C323-C324	120.00
C325-C324-C323	120.00	C325-C324-C327	119.1(13)
C323-C324-C327	119.0(13)	C324-C325-C326	120.00
C325-C326-C321	120.00	C332-C331-C336	120.00
C332-C331-S33	117.(3)	C336-C331-S33	123.(3)
C331-C332-C333	120.00	C332-C333-C334	120.00
C335-C334-C333	120.00	C335-C334-C337	113.(6)
C333-C334-C337	127.(6)	C334-C335-C336	120.00
C335-C336-C331	120.00	C342-C341-C346	120.00
C342-C341-S34	118.(3)	C346-C341-S34	121.(3)
C343-C342-C341	120.00	C342-C343-C344	120.00
C345-C344-C343	120.00	C345-C344-C347	119.8(12)
C343-C344-C347	119.9(12)	C346-C345-C344	120.00
C345-C346-C341	120.00	C352-C351-C356	120.00
C352-C351-S35	121.(4)	C356-C351-S35	118.(4)
C353-C352-C351	120.00	C354-C353-C352	120.00
C353-C354-C355	120.00	C353-C354-C357	120.2(12)
C355-C354-C357	119.7(12)	C356-C355-C354	120.00
C355-C356-C351	120.00	C362-C361-C366	120.00
C362-C361-S36	126.(3)	C366-C361-S36	114.(3)
C363-C362-C361	120.00	C362-C363-C364	120.00
C365-C364-C363	120.00	C365-C364-C367	123.(5)
C363-C364-C367	116.(5)	C364-C365-C366	120.00
C365-C366-C361	120.00	C372-C371-C376	120.00
C372-C371-S37	127.(4)	C376-C371-S37	113.(4)
C373-C372-C371	120.00	C374-C373-C372	120.00
C373-C374-C375	120.00	C373-C374-C377	124.(6)
C375-C374-C377	116.(6)	C374-C375-C376	120.00
C375-C376-C371	120.00	C382-C381-C386	120.00
C382-C381-S38	119.3(10)	C386-C381-S38	120.2(10)
C383-C382-C381	120.00	C382-C383-C384	120.00
C385-C384-C383	120.00	C385-C384-C387	117.5(16)

C383-C384-C387	117.1(16)	C384-C385-C386	120.00
C385-C386-C381	120.00	C392-C391-C396	120.00
C392-C391-S39	119.4(12)	C396-C391-S39	119.5(12)
C391-C392-C393	120.00	C394-C393-C392	120.00
C393-C394-C395	120.00	C393-C394-C397	115.(6)
C395-C394-C397	125.(6)	C396-C395-C394	120.00(5)
C395-C396-C391	120.00	C402-C401-C406	120.00
C402-C401-S40	110.(5)	C406-C401-S40	129.(5)
C401-C402-C403	120.00	C404-C403-C402	120.00
C403-C404-C405	120.00	C403-C404-C407	129.(7)
C405-C404-C407	111.(7)	C406-C405-C404	120.00
C405-C406-C401	120.00	C412_1-C411_1-C416_1	120.00
C412_1-C411_1-S41	119.9(11)	C416_1-C411_1-S41	120.1(11)
C411_1-C412_1-C413_1	120.00	C414_1-C413_1-C412_1	120.00
C415_1-C414_1-C413_1	120.00	C415_1-C414_1-C417_1	119.9(12)
C413_1-C414_1-C417_1	120.1(12)	C414_1-C415_1-C416_1	120.00
C415_1-C416_1-C411_1	120.00	C412_2-C411_2-C416_2	120.00
C412_2-C411_2-S41	120.0(11)	C416_2-C411_2-S41	120.0(11)
C413_2-C412_2-C411_2	120.00	C412_2-C413_2-C414_2	120.00
C415_2-C414_2-C413_2	120.00	C415_2-C414_2-C417_2	120.1(12)
C413_2-C414_2-C417_2	119.8(12)	C414_2-C415_2-C416_2	120.00
C415_2-C416_2-C411_2	120.00	C422-C421-C426	120.00
C422-C421-S42	120.0(11)	C426-C421-S42	119.9(11)
C423-C422-C421	120.00	C422-C423-C424	120.00
C425-C424-C423	120.00	C425-C424-C427	109.(5)
C423-C424-C427	128.(5)	C424-C425-C426	120.00
C425-C426-C421	120.00		

Table S6. Anisotropic atomic displacement parameters (Å^2) for $Au_{76}(S\text{-}C_6H_4\text{-}p\text{-}Me)_{42}$						
The anisotropic atomic displacement factor exponent takes the form: $-2\pi^2[h^2 a^{*2} U_{11} + ... + 2 h k a^* b^* U_{12}]$						
	**U_{11}**	**U_{22}**	**U_{33}**	**U_{23}**	**U_{13}**	**U_{12}**
Au1	0.071(2)	0.0694(19)	0.0667(18)	-0.0015(15)	-0.0017(15)	0.0028(15)
Au2	0.076(2)	0.075(2)	0.073(2)	-0.0052(15)	-0.0030(16)	0.0042(17)
Au3	0.075(2)	0.0676(19)	0.0729(19)	-0.0036(15)	0.0017(16)	-0.0005(16)
Au4	0.071(2)	0.0692(19)	0.0702(19)	-0.0032(15)	0.0000(16)	-0.0005(15)
Au5	0.071(2)	0.074(2)	0.075(2)	-0.0057(15)	-0.0013(16)	0.0000(16)
Au6	0.077(2)	0.0644(19)	0.0692(19)	-0.0019(14)	-0.0037(15)	0.0001(16)
Au7	0.071(2)	0.075(2)	0.076(2)	-0.0026(16)	-0.0024(16)	-0.0021(17)
Au8	0.073(2)	0.0700(19)	0.0678(18)	-0.0037(15)	-0.0030(15)	0.0003(16)
Au9	0.079(2)	0.083(2)	0.072(2)	-0.0012(16)	-0.0009(16)	0.0026(17)
Au10	0.075(2)	0.0655(19)	0.0707(19)	-0.0022(15)	0.0001(15)	-0.0004(16)
Au11	0.076(2)	0.0689(19)	0.077(2)	-0.0017(16)	0.0008(16)	-0.0012(16)
Au12	0.072(2)	0.0671(18)	0.0697(18)	-0.0028(15)	-0.0001(15)	-0.0022(15)
Au13	0.080(2)	0.0659(19)	0.0703(19)	-0.0037(15)	0.0010(16)	-0.0017(16)
Au14	0.070(2)	0.070(2)	0.0742(19)	-0.0064(15)	0.0008(16)	-0.0004(15)
Au15	0.084(2)	0.086(2)	0.071(2)	-0.0066(16)	-0.0022(17)	-0.0039(18)
Au16	0.075(2)	0.072(2)	0.077(2)	-0.0053(15)	0.0036(16)	-0.0020(17)
Au17	0.084(2)	0.081(2)	0.083(2)	-0.0118(17)	0.0011(17)	-0.0039(17)
Au18	0.076(2)	0.076(2)	0.084(2)	-0.0082(16)	0.0061(17)	-0.0011(17)
Au19	0.076(2)	0.074(2)	0.089(2)	-0.0138(17)	0.0005(17)	-0.0070(16)
Au20	0.071(2)	0.067(2)	0.079(2)	-0.0048(15)	0.0003(16)	0.0003(16)
Au21	0.078(2)	0.082(2)	0.080(2)	-0.0090(16)	-0.0005(17)	-0.0093(17)
Au22	0.079(2)	0.067(2)	0.0728(19)	-0.0003(15)	-0.0027(16)	0.0001(16)
Au23	0.080(2)	0.080(2)	0.075(2)	0.0005(16)	0.0019(16)	-0.0054(17)
Au24	0.078(2)	0.067(2)	0.074(2)	0.0017(15)	-0.0018(16)	-0.0029(16)
Au25	0.075(2)	0.067(2)	0.078(2)	0.0000(15)	-0.0025(16)	-0.0022(16)
Au26	0.078(2)	0.077(2)	0.079(2)	-0.0010(16)	0.0012(17)	-0.0070(17)
Au27	0.092(2)	0.074(2)	0.084(2)	0.0009(17)	-0.0057(18)	-0.0112(17)
Au28	0.085(2)	0.088(2)	0.079(2)	0.0007(17)	0.0023(17)	-0.0070(18)
Au29	0.087(2)	0.075(2)	0.078(2)	0.0059(16)	-0.0079(17)	-0.0032(17)
Au30	0.084(2)	0.0683(19)	0.081(2)	-0.0057(16)	-0.0027(17)	-0.0013(17)
Au31	0.104(3)	0.078(2)	0.095(2)	0.0043(17)	-0.0070(19)	-0.0099(19)
Au32	0.094(2)	0.075(2)	0.110(3)	-0.0020(19)	-0.004(2)	-0.0097(18)
Au33	0.080(2)	0.082(2)	0.097(2)	-0.0092(17)	0.0031(18)	-0.0004(18)
Au34	0.085(2)	0.080(2)	0.084(2)	0.0002(17)	-0.0016(18)	0.0021(18)
Au35	0.086(2)	0.077(2)	0.073(2)	0.0036(16)	0.0016(17)	0.0052(17)
Au36	0.094(2)	0.075(2)	0.080(2)	0.0033(16)	-0.0061(18)	0.0073(17)
Au37	0.077(2)	0.075(2)	0.0720(19)	-0.0039(15)	-0.0016(16)	0.0088(17)
Au38	0.087(2)	0.076(2)	0.085(2)	0.0085(17)	-0.0040(17)	-0.0097(17)
Au39	0.081(2)	0.076(2)	0.082(2)	-0.0051(16)	-0.0028(17)	0.0041(17)
Au40	0.078(2)	0.0692(19)	0.0698(19)	0.0029(15)	0.0015(16)	0.0007(16)
Au41	0.095(2)	0.082(2)	0.098(2)	-0.0147(18)	0.0048(19)	-0.0101(18)

	U_{11}	U_{22}	U_{33}	U_{23}	U_{13}	U_{12}
Au42	0.090(2)	0.082(2)	0.074(2)	0.0029(16)	0.0022(17)	-0.0046(18)
Au43	0.107(3)	0.088(2)	0.094(2)	-0.0116(18)	-0.004(2)	0.009(2)
Au44	0.086(2)	0.086(2)	0.085(2)	0.0024(17)	0.0003(18)	0.0051(18)
Au45	0.080(2)	0.072(2)	0.0709(19)	-0.0038(15)	-0.0032(16)	-0.0004(16)
Au46	0.098(3)	0.083(2)	0.116(3)	-0.0059(19)	0.013(2)	-0.0092(19)
Au47	0.086(2)	0.087(2)	0.085(2)	-0.0056(17)	0.0130(17)	0.0004(18)
Au48	0.129(3)	0.113(3)	0.095(2)	-0.017(2)	0.009(2)	-0.011(2)
Au49	0.088(2)	0.079(2)	0.072(2)	-0.0051(16)	-0.0025(17)	-0.0005(18)
Au50	0.125(3)	0.084(2)	0.111(3)	0.000(2)	-0.002(2)	0.000(2)
Au51	0.083(2)	0.082(2)	0.074(2)	0.0018(16)	-0.0041(17)	-0.0033(17)
Au52	0.097(3)	0.138(3)	0.084(2)	0.001(2)	0.0024(19)	-0.014(2)
Au53	0.094(2)	0.083(2)	0.083(2)	-0.0005(17)	-0.0023(18)	0.0054(18)
Au54	0.085(2)	0.081(2)	0.084(2)	-0.0090(17)	-0.0019(17)	-0.0042(18)
Au55	0.118(3)	0.113(3)	0.093(2)	-0.003(2)	-0.015(2)	-0.011(2)
Au56	0.118(3)	0.088(2)	0.197(4)	-0.009(3)	0.007(3)	0.008(2)
Au57	0.076(2)	0.074(2)	0.077(2)	-0.0019(16)	0.0061(16)	-0.0016(17)
Au58	0.083(2)	0.082(2)	0.083(2)	0.0059(17)	-0.0062(17)	-0.0070(17)
Au59	0.079(2)	0.084(2)	0.080(2)	0.0024(17)	-0.0066(17)	-0.0041(17)
Au60	0.091(2)	0.083(2)	0.079(2)	0.0057(17)	-0.0093(17)	-0.0123(18)
Au61	0.115(3)	0.089(2)	0.092(2)	-0.0004(19)	-0.005(2)	-0.017(2)
Au62	0.078(2)	0.069(2)	0.078(2)	0.0003(16)	-0.0052(16)	-0.0033(16)
Au63	0.087(2)	0.0677(19)	0.083(2)	-0.0019(16)	0.0001(17)	-0.0077(17)
Au64	0.081(2)	0.069(2)	0.080(2)	0.0022(16)	-0.0051(17)	0.0011(16)
Au65	0.103(3)	0.086(2)	0.087(2)	-0.0073(18)	0.0095(19)	-0.0145(19)
Au66	0.100(3)	0.114(3)	0.093(2)	-0.002(2)	-0.011(2)	0.006(2)
Au67	0.073(2)	0.074(2)	0.080(2)	-0.0034(16)	-0.0024(16)	-0.0036(16)
Au68	0.083(2)	0.073(2)	0.081(2)	-0.0037(16)	0.0043(17)	-0.0030(17)
Au69	0.077(2)	0.071(2)	0.093(2)	-0.0026(17)	-0.0014(17)	0.0011(17)
Au70	0.090(2)	0.087(2)	0.086(2)	-0.0014(17)	0.0106(18)	-0.0056(18)
Au71	0.077(2)	0.073(2)	0.106(2)	0.0016(18)	-0.0037(18)	0.0040(17)
Au72	0.087(2)	0.091(2)	0.093(2)	-0.0065(18)	0.0076(18)	0.0000(19)
Au73	0.085(2)	0.083(2)	0.099(2)	-0.0037(18)	0.0019(19)	0.0084(18)
Au74	0.114(3)	0.089(2)	0.121(3)	0.000(2)	-0.001(2)	0.014(2)
Au75	0.089(2)	0.084(2)	0.078(2)	-0.0020(17)	-0.0031(17)	0.0064(18)
Au76	0.093(3)	0.131(3)	0.094(2)	0.004(2)	0.008(2)	-0.013(2)
S1	0.075(12)	0.075(12)	0.077(11)	0.005(9)	-0.010(10)	-0.007(9)
S2	0.095(14)	0.074(12)	0.083(12)	0.000(10)	0.013(10)	-0.016(10)
S3	0.115(16)	0.081(13)	0.117(16)	0.000(12)	0.003(13)	-0.001(11)
S4	0.086(13)	0.082(12)	0.090(13)	-0.009(10)	0.013(10)	-0.036(10)
S5	0.098(15)	0.076(13)	0.120(15)	0.005(11)	0.000(12)	0.022(11)
S6	0.084(14)	0.111(15)	0.107(15)	-0.001(12)	0.016(11)	-0.008(11)
S7	0.080(13)	0.093(14)	0.095(13)	0.004(11)	-0.008(11)	0.020(10)
S8	0.074(12)	0.104(14)	0.064(11)	0.002(9)	0.005(9)	-0.016(11)
S9	0.17(2)	0.137(19)	0.15(2)	0.008(16)	-0.034(17)	-0.062(17)

	U_{11}	U_{22}	U_{33}	U_{23}	U_{13}	U_{12}
S10	0.090(13)	0.061(11)	0.080(12)	-0.015(9)	-0.007(10)	0.000(9)
S11	0.114(15)	0.070(12)	0.090(13)	-0.029(10)	0.006(11)	-0.009(11)
S12	0.073(12)	0.072(12)	0.094(13)	-0.016(10)	0.018(10)	-0.005(9)
S13	0.087(13)	0.092(13)	0.067(11)	0.002(9)	0.012(10)	0.014(10)
S14	0.069(13)	0.111(15)	0.116(15)	0.001(12)	0.016(11)	0.001(11)
S15	0.060(11)	0.063(11)	0.089(12)	-0.001(9)	-0.023(9)	0.007(9)
S16	0.088(13)	0.102(14)	0.077(12)	0.003(10)	-0.009(10)	-0.025(11)
S17	0.070(12)	0.084(12)	0.075(11)	-0.007(9)	0.002(9)	0.008(10)
S18	0.115(16)	0.095(14)	0.102(14)	0.002(11)	-0.011(12)	0.013(12)
S19	0.080(13)	0.105(14)	0.067(11)	0.012(10)	-0.006(9)	0.003(11)
S20	0.094(14)	0.129(16)	0.075(12)	-0.013(11)	-0.002(11)	-0.004(12)
S21	0.062(11)	0.066(11)	0.097(13)	0.008(9)	0.000(10)	-0.010(9)
S22	0.054(11)	0.087(13)	0.096(13)	-0.002(10)	0.010(9)	0.011(10)
S23	0.095(14)	0.072(12)	0.085(12)	-0.016(10)	-0.014(10)	0.009(10)
S24	0.092(13)	0.081(12)	0.063(11)	0.001(9)	0.010(9)	0.009(10)
S25	0.110(15)	0.097(14)	0.070(12)	0.001(10)	-0.020(11)	0.010(11)
S26	0.105(16)	0.18(2)	0.107(16)	0.028(15)	-0.002(13)	-0.038(15)
S27	0.123(17)	0.142(18)	0.071(13)	-0.024(12)	0.031(11)	-0.004(14)
S28	0.125(16)	0.069(12)	0.090(13)	0.011(10)	-0.003(12)	-0.038(11)
S29	0.100(18)	0.35(4)	0.078(15)	-0.047(18)	0.013(13)	-0.02(2)
S30	0.115(16)	0.092(14)	0.109(15)	0.015(12)	0.020(12)	0.016(12)
S31	0.070(13)	0.174(19)	0.069(12)	-0.005(12)	-0.003(10)	0.006(13)
S32	0.081(13)	0.087(13)	0.117(15)	-0.004(11)	0.002(11)	0.009(11)
S33	0.131(16)	0.080(13)	0.078(12)	0.015(10)	0.003(11)	0.038(12)
S34	0.089(14)	0.106(15)	0.094(14)	0.002(11)	-0.012(11)	0.023(11)
S35	0.122(18)	0.136(18)	0.119(17)	-0.015(14)	0.015(14)	-0.032(14)
S36	0.087(13)	0.101(14)	0.077(12)	-0.011(10)	-0.009(10)	0.013(11)
S37	0.132(17)	0.086(13)	0.098(14)	0.000(11)	0.007(13)	-0.044(12)
S38	0.129(18)	0.099(15)	0.132(18)	-0.015(13)	0.022(14)	-0.040(13)
S39	0.129(18)	0.076(14)	0.22(2)	-0.032(15)	0.004(17)	-0.021(13)
S40	0.16(2)	0.097(16)	0.18(2)	0.000(16)	-0.009(18)	-0.001(15)
S41	0.18(2)	0.065(14)	0.25(3)	-0.011(15)	-0.06(2)	0.064(15)
S42	0.14(2)	0.16(2)	0.139(19)	0.036(16)	-0.067(16)	-0.044(16)

**Table S7. Hydrogen atomic coordinates and isotropic atomic displacement parameters (Å²) for $Au_{76}(S\text{-}C_6H_4\text{-}p\text{-}Me)_{42}$.**				
	**x/a**	**y/b**	**z/c**	**U(eq)**
H12	0.4861	0.1645	0.3921	0.12000
H13	0.4548	0.1601	0.3339	0.13300
H15	0.4792	0.2940	0.3035	0.13100
H16	0.5105	0.2984	0.3617	0.14800
H17A	0.4471	0.2074	0.2556	0.32600
H17B	0.4422	0.2621	0.2649	0.32600
H17C	0.4202	0.2239	0.2857	0.32600
H22	0.5042	0.0649	0.4737	0.17700
H23	0.4594	0.0543	0.4345	0.11600
H25	0.4314	0.1870	0.4634	0.09900
H26	0.4763	0.1976	0.5025	0.10100
H27A	0.4056	0.1349	0.4035	0.22100
H27B	0.4001	0.0861	0.4267	0.22100
H27C	0.4249	0.0892	0.3908	0.22100
H32	0.4719	0.1721	0.6011	0.18500
H33	0.4222	0.1663	0.5728	0.21500
H35	0.4194	0.3088	0.5598	0.32500
H36	0.4691	0.3146	0.5881	0.17100
H37A	0.3739	0.2610	0.5501	0.22400
H37B	0.3745	0.2066	0.5627	0.22400
H37C	0.3864	0.2224	0.5187	0.22400
H46	0.4519	0.1987	0.6909	0.13600
H44	0.4050	0.1918	0.7262	0.16000
H42	0.4387	0.0788	0.7880	0.27100
H43	0.4857	0.0856	0.7527	0.17000
H47A	0.3721	0.1587	0.7655	0.22800
H47B	0.3845	0.1359	0.8068	0.22800
H47C	0.3746	0.1028	0.7697	0.22800
H52	0.4465	0.2852	0.6956	0.25600
H53	0.4026	0.2782	0.7372	0.20800
H55	0.4186	0.4092	0.7830	0.17300
H56	0.4625	0.4162	0.7414	0.16800
H57A	0.3658	0.3056	0.7774	0.32500
H57B	0.3618	0.3611	0.7840	0.32500
H57C	0.3802	0.3303	0.8167	0.32500
H62	0.4385	0.1794	0.8162	0.26300
H63	0.4051	0.1850	0.8723	0.23700
H65	0.4436	0.3109	0.9028	0.33100
H66	0.4770	0.3054	0.8467	0.30700
H67A	0.3908	0.2191	0.9364	0.31900
H67B	0.3917	0.2753	0.9386	0.31900

	x/a	y/b	z/c	U(eq)
H67C	0.4195	0.2452	0.9571	0.31900
H72	0.4600	0.2835	0.4911	0.16900
H73	0.4283	0.2811	0.4331	0.12800
H75	0.4671	0.4027	0.3922	0.13700
H76	0.4989	0.4051	0.4501	0.15400
H77A	0.4369	0.3343	0.3405	0.35400
H77B	0.4060	0.3499	0.3632	0.35400
H77C	0.4173	0.2964	0.3653	0.35400
H82	0.7730	0.3922	0.6018	0.19900
H83	0.8085	0.4536	0.6121	0.20400
H85	0.7741	0.5221	0.5132	0.22500
H86	0.7386	0.4607	0.5029	0.12900
H87A	0.8331	0.5261	0.5594	0.38700
H87B	0.8093	0.5483	0.5913	0.38700
H87C	0.8039	0.5551	0.5436	0.38700
H92	0.6476	0.4920	0.4343	0.23300
H93	0.6764	0.5223	0.3797	0.25200
H95	0.7384	0.5776	0.4596	0.34500
H96	0.7095	0.5473	0.5142	0.39400
H97A	0.7398	0.5480	0.3644	0.33400
H97B	0.7442	0.5961	0.3891	0.33400
H97C	0.7151	0.5895	0.3596	0.33400
H102	0.7145	0.4683	0.7751	0.10400
H103	0.7555	0.5220	0.7810	0.15100
H105	0.8025	0.4514	0.6928	0.17500
H106	0.7615	0.3977	0.6869	0.18300
H10A	0.8264	0.5088	0.7523	0.35900
H10B	0.8030	0.5520	0.7557	0.35900
H10C	0.8145	0.5344	0.7120	0.35900
H112	0.6902	0.5467	0.7598	0.12500
H113	0.6994	0.5763	0.8255	0.13100
H115	0.6119	0.5415	0.8586	0.13500
H116	0.6027	0.5119	0.7929	0.13300
H11A	0.6779	0.6063	0.8922	0.18000
H11B	0.6695	0.5557	0.9106	0.18000
H11C	0.6430	0.5941	0.9040	0.18000
H122	0.6881	0.4303	0.8536	0.11900
H123	0.7028	0.4547	0.9192	0.13200
H125	0.6127	0.4555	0.9541	0.16200
H126	0.5980	0.4310	0.8886	0.13500
H12A	0.6912	0.4764	0.9879	0.25400
H12B	0.6652	0.4417	1.0048	0.25400
H12C	0.6574	0.4960	0.9974	0.25400
H132	0.4827	0.4077	0.8106	0.19000

	x/a	y/b	z/c	U(eq)
H133	0.4583	0.4811	0.8178	0.11000
H135	0.5418	0.5438	0.8343	0.11700
H136	0.5662	0.4704	0.8272	0.09800
H13A	0.4691	0.5727	0.8018	0.25500
H13B	0.4890	0.5914	0.8395	0.25500
H13C	0.4593	0.5592	0.8472	0.25500
H142	0.4827	0.5087	0.7105	0.13500
H143	0.4531	0.5769	0.7213	0.18700
H145	0.5312	0.6505	0.7473	0.14500
H146	0.5608	0.5823	0.7365	0.11900
H14A	0.4674	0.6783	0.7135	0.21700
H14B	0.4824	0.6864	0.7573	0.21700
H14C	0.4513	0.6568	0.7530	0.21700
H152	0.6103	0.4556	0.3998	0.13300
H153	0.6322	0.4755	0.3367	0.20500
H155	0.5980	0.3547	0.2875	0.14800
H156	0.5760	0.3348	0.3506	0.08400
H15A	0.6537	0.4428	0.2690	0.28500
H15B	0.6209	0.4480	0.2473	0.28500
H15C	0.6373	0.3977	0.2494	0.28500
H162	0.7223	0.4374	0.4250	0.15200
H163	0.7561	0.4617	0.3731	0.18000
H165	0.7063	0.3868	0.2904	0.16200
H166	0.6725	0.3625	0.3422	0.13900
H16A	0.7472	0.4219	0.2694	0.30300
H16B	0.7732	0.4429	0.2988	0.30300
H16C	0.7444	0.4747	0.2857	0.30300
H172	0.7743	0.1341	0.4998	0.11300
H173	0.8208	0.0923	0.5060	0.30800
H175	0.8275	0.1332	0.6244	0.15600
H176	0.7810	0.1750	0.6182	0.13600
H17D	0.8609	0.0531	0.5717	0.23600
H17E	0.8745	0.1022	0.5873	0.23600
H17F	0.8719	0.0919	0.5396	0.23600
H182	0.6712	-0.0703	0.5578	0.13100
H183	0.6945	-0.0883	0.4952	0.16800
H185	0.7791	-0.0815	0.5486	0.12900
H186	0.7558	-0.0635	0.6113	0.14500
H18A	0.7446	-0.0711	0.4550	0.27400
H18B	0.7524	-0.1248	0.4654	0.27400
H18C	0.7764	-0.0842	0.4769	0.27400
H192	0.7900	0.0392	0.5937	0.13200
H193	0.8273	-0.0209	0.5956	0.16300
H195	0.7932	-0.0655	0.7044	0.16100

	x/a	y/b	z/c	U(eq)
H196	0.7559	-0.0054	0.7025	0.13300
H19A	0.8279	-0.1167	0.6398	0.19300
H19B	0.8310	-0.1014	0.6864	0.19300
H19C	0.8554	-0.0829	0.6536	0.19300
H202	0.7496	0.2966	0.7739	0.17300
H203	0.7909	0.3503	0.7740	0.18800
H205	0.7587	0.4078	0.8792	0.14900
H206	0.7174	0.3541	0.8791	0.17400
H20A	0.8240	0.4000	0.8063	0.24300
H20B	0.8170	0.4221	0.8501	0.24300
H20C	0.8031	0.4461	0.8101	0.24300
H212	0.6261	-0.0580	0.4989	0.12000
H213	0.6060	-0.1341	0.4897	0.12100
H215	0.5198	-0.0851	0.5146	0.15900
H216	0.5399	-0.0089	0.5238	0.10500
H21A	0.5267	-0.1690	0.4956	0.25300
H21B	0.5585	-0.1898	0.5124	0.25300
H21C	0.5550	-0.1775	0.4652	0.25300
H222	0.7474	0.0564	0.5120	0.13200
H223	0.7740	0.0467	0.4502	0.13100
H225	0.6946	0.0031	0.3958	0.11900
H226	0.6680	0.0129	0.4575	0.11100
H22A	0.7483	-0.0106	0.3623	0.20500
H22B	0.7756	0.0222	0.3783	0.20500
H22C	0.7462	0.0453	0.3568	0.20500
H232	0.6366	0.0490	0.4104	0.12300
H233	0.6239	-0.0304	0.3992	0.21900
H235	0.5326	0.0020	0.4077	0.12800
H236	0.5453	0.0815	0.4189	0.09000
H23A	0.5779	-0.0818	0.3653	0.13700
H23B	0.5471	-0.0785	0.3919	0.13700
H23C	0.5787	-0.0946	0.4126	0.13700
H242	0.6718	0.1198	0.3640	0.14800
H243	0.6976	0.1196	0.3011	0.14100
H245	0.7757	0.1661	0.3568	0.14600
H246	0.7499	0.1663	0.4197	0.08500
H24A	0.7707	0.1794	0.2891	0.22800
H24B	0.7439	0.1566	0.2622	0.22800
H24Z	0.7709	0.1242	0.2798	0.22800
H252	0.5445	0.1875	0.3202	0.10300
H253	0.5338	0.1081	0.3057	0.10700
H255	0.6259	0.0800	0.3022	0.12100
H256	0.6366	0.1594	0.3167	0.10700
H25A	0.5701	0.0282	0.2649	0.21700

	x/a	y/b	z/c	U(eq)
H25B	0.5510	0.0260	0.3065	0.21700
H25C	0.5870	0.0137	0.3063	0.21700
H262	0.6771	0.2932	0.2544	0.22000
H263	0.7024	0.3201	0.1956	0.19300
H265	0.7848	0.3122	0.2546	0.18500
H266	0.7595	0.2853	0.3134	0.17100
H26A	0.7627	0.3784	0.1962	0.34100
H26B	0.7427	0.3483	0.1646	0.34100
H26C	0.7769	0.3320	0.1760	0.34100
H272	0.4932	0.1959	0.8981	0.11200
H273	0.4877	0.1149	0.9111	0.15600
H275	0.5766	0.1093	0.9507	0.16600
H276	0.5821	0.1903	0.9377	0.17800
H27D	0.5330	0.0352	0.9218	0.23000
H27E	0.5116	0.0496	0.9594	0.23000
H27F	0.5482	0.0504	0.9641	0.23000
H282	0.5891	-0.0647	0.7158	0.12100
H283	0.5578	-0.1321	0.7118	0.12900
H285	0.4814	-0.0507	0.7280	0.22200
H286	0.5128	0.0167	0.7320	0.12700
H28A	0.4728	-0.1386	0.7074	0.30400
H28B	0.4927	-0.1635	0.7422	0.30400
H28C	0.5024	-0.1692	0.6956	0.30400
H292	0.7900	0.2693	0.5146	0.27600
H293	0.8244	0.3303	0.5315	0.32600
H295	0.7874	0.4121	0.4422	0.30400
H296	0.7530	0.3511	0.4253	0.30000
H29A	0.8567	0.3899	0.4769	0.39900
H29B	0.8444	0.4192	0.5151	0.39900
H29C	0.8361	0.4359	0.4700	0.39900
H302	0.7381	0.5492	0.6936	0.31500
H303	0.7571	0.6257	0.7045	0.31800
H305	0.7430	0.6567	0.5850	0.35600
H306	0.7240	0.5802	0.5741	0.36800
H30A	0.7778	0.7071	0.6222	0.30500
H30B	0.7887	0.6931	0.6670	0.30500
H30C	0.7575	0.7220	0.6606	0.30500
H312	0.7711	0.2858	0.5836	0.17900
H313	0.8219	0.2597	0.5945	0.16600
H315	0.8076	0.2631	0.7169	0.18500
H316	0.7568	0.2891	0.7060	0.17900
H31A	0.8577	0.2094	0.6763	0.32600
H31B	0.8686	0.2630	0.6799	0.32600
H31C	0.8675	0.2380	0.6364	0.32600

	x/a	y/b	z/c	U(eq)
H322	0.7308	0.1092	0.7861	0.19500
H323	0.7711	0.0567	0.8017	0.18500
H325	0.8244	0.1173	0.7142	0.19300
H326	0.7842	0.1697	0.6986	0.15400
H32A	0.8390	0.0442	0.7848	0.35200
H32B	0.8365	0.0371	0.7367	0.35200
H32C	0.8132	0.0101	0.7663	0.35200
H332	0.6916	-0.0277	0.7217	0.13800
H333	0.6752	-0.1061	0.7295	0.12500
H335	0.6054	-0.0621	0.8032	0.34500
H336	0.6219	0.0162	0.7954	0.22600
H33A	0.6063	-0.1339	0.7908	0.34300
H33B	0.6400	-0.1570	0.7900	0.34300
H33C	0.6204	-0.1530	0.7490	0.34300
H342	0.6323	0.1923	0.9126	0.20200
H343	0.6573	0.2007	0.9756	0.23800
H345	0.7387	0.1627	0.9232	0.16500
H346	0.7136	0.1543	0.8601	0.17800
H34A	0.7114	0.2122	1.0121	0.26700
H34B	0.7386	0.1801	0.9947	0.26700
H34C	0.7087	0.1561	1.0141	0.26700
H352	0.6982	0.3222	0.9656	0.24300
H353	0.7170	0.3148	1.0324	0.29800
H355	0.6296	0.2934	1.0735	0.29700
H356	0.6107	0.3007	1.0067	0.22900
H35A	0.7098	0.3030	1.1040	0.42700
H35B	0.6804	0.2724	1.1170	0.42700
H35C	0.6790	0.3286	1.1193	0.42700
H362	0.5028	0.1008	0.8181	0.10500
H363	0.4869	0.0226	0.8291	0.15100
H365	0.5764	-0.0165	0.8456	0.11200
H366	0.5922	0.0617	0.8345	0.14400
H36A	0.5316	-0.0771	0.8267	0.40800
H36B	0.4971	-0.0577	0.8300	0.40800
H36C	0.5171	-0.0654	0.8703	0.40800
H372	0.5017	-0.0484	0.5862	0.16900
H373	0.4577	-0.0547	0.5448	0.23100
H375	0.4432	0.0864	0.5555	0.23900
H376	0.4872	0.0927	0.5970	0.16500
H37F	0.4140	0.0450	0.5029	0.30100
H37D	0.3974	0.0091	0.5333	0.30100
H37E	0.4180	-0.0104	0.4966	0.30100
H382	0.5334	-0.1452	0.5993	0.16800
H383	0.5158	-0.2179	0.6245	0.20600

	x/a	y/b	z/c	U(eq)
H385	0.5968	-0.2353	0.6854	0.21200
H386	0.6143	-0.1626	0.6603	0.14900
H38A	0.5491	-0.2985	0.6891	0.39600
H38B	0.5167	-0.2742	0.6791	0.39600
H38C	0.5375	-0.2545	0.7154	0.39600
H392	0.6071	0.6014	0.6958	0.48000
H393	0.6101	0.6424	0.7579	0.39400
H395	0.6886	0.7071	0.7194	0.47000
H396	0.6857	0.6661	0.6574	0.54900
H39A	0.6591	0.6718	0.8071	0.45000
H39B	0.6316	0.7079	0.7985	0.45000
H39C	0.6665	0.7235	0.7905	0.45000
H402	0.4759	0.6263	0.6095	0.26600
H403	0.4504	0.6865	0.5737	0.27100
H405	0.5328	0.7337	0.5307	0.19900
H406	0.5583	0.6735	0.5665	0.16700
H40A	0.4916	0.7548	0.4971	0.27800
H40B	0.4770	0.7829	0.5346	0.27800
H40C	0.4570	0.7442	0.5112	0.27800
H412_1	0.4491	0.4665	0.6640	0.31100
H413_1	0.4020	0.5063	0.6727	0.31400
H415_1	0.4109	0.5639	0.5598	0.37300
H416_1	0.4580	0.5241	0.5511	0.42200
H41A_1	0.3540	0.5470	0.6096	0.51700
H41B_1	0.3717	0.5964	0.6103	0.51700
H41C_1	0.3659	0.5683	0.6517	0.51700
H412_2	0.4785	0.5180	0.5305	0.43400
H413_2	0.4423	0.5128	0.4775	0.43900
H415_2	0.4125	0.3870	0.5221	0.44700
H416_2	0.4487	0.3922	0.5752	0.45700
H41A_2	0.3803	0.4380	0.4685	0.28200
H41B_2	0.4078	0.4193	0.4403	0.28200
H41C_2	0.4007	0.4744	0.4429	0.28200
H422	0.5476	0.5722	0.5285	0.32600
H423	0.5096	0.6249	0.5055	0.30500
H425	0.4905	0.5373	0.4107	0.24200
H426	0.5285	0.4846	0.4338	0.28500
H42A	0.4937	0.6432	0.4175	0.27600
H42B	0.4633	0.6362	0.4442	0.27600
H42C	0.4697	0.6026	0.4061	0.27600

Supporting references:

(1) M. J. Frisch, G. W. Trucks, H. B. Schlegel, G. E. Scuseria, M. A. Robb, J. R. Cheeseman, G. Scalmani, V. Barone, G. A. Petersson, H. Nakatsuji, X. Li, M. Caricato, A. V. Marenich, J. Bloino, B. G. Janesko, R. Gomperts, B. Mennucci, H. P. Hratchian, J. V. Ortiz, A. F. Izmaylov, J. L. Sonnenberg, D. Williams-Young, F. Ding, F. Lipparini, F. Egidi, J. Goings, B. Peng, A. Petrone, T. Henderson, D. Ranasinghe, V. G. Zakrzewski, J. Gao, N. Rega, G. Zheng, W. Liang, M. Hada, M. Ehara, K. Toyota, R. Fukuda, J. Hasegawa, M. Ishida, T. Nakajima, Y. Honda, O. Kitao, H. Nakai, T. Vreven, K. Throssell, J. A. Montgomery, Jr., J. E. Peralta, F. Ogliaro, M. J. Bearpark, J. J. Heyd, E. N. Brothers, K. N. Kudin, V. N. Staroverov, T. A. Keith, R. Kobayashi, J. Normand, K. Raghavachari, A. P. Rendell, J. C. Burant, S. S. Iyengar, J. Tomasi, M. Cossi, J. M. Millam, M. Klene, C. Adamo, R. Cammi, J. W. Ochterski, R. L. Martin, K. Morokuma, O. Farkas, J. B. Foresman, and D. J. Fox. Gaussian 16 Revision B.01. Gaussian Inc. Wallingford CT 2016.

(2) P. J. Hay, W. R. Wadt, Ab Initio Effective Core Potentials for Molecular Calculations. Potentials for the Transition Metal Atoms Sc to Hg. *J. Chem. Phys.* **1985**, *82*(1), 270–283.

(3) J. P. Perdew, K. Burke, M. Ernzerhof, Generalized Gradient Approximation Made Simple. *Phys. Rev. Lett*. **1996**, *77*, 3865–3868.

(4) D. B. Axel, Density‐Functional Thermochemistry. III. The Role of Exact Exchange. *J. Chem. Phys.* **1993**, *98*, 5648–5652.

(5) C. Lee, W. Yang, R. G. Parr, Development of the Colle–Salvetti Correlation-Energy Formula into a Functional of the Electron Density. *Phys. Rev. B* **1988**, *37*, 785.

(6) P. J. Stephens, F. J. Devlin, C. F. Chabalowski, M. J. Frisch, Ab Initio Calculation of Vibrational Absorption and Circular Dichroism Spectra Using Density Functional Force Fields. *J. Phys. Chem.* **1994**, *98*, 11623−11627.

(7) S. Grimme, S. Ehrlich, L. Goerigk, Effect of the Damping Function in Dispersion Corrected Density Functional Theory. *J. Comput. Chem.* **2011**, *32*, 1456–1465.
